# Microvascular Health as a Key Determinant of Organismal Aging

**DOI:** 10.1002/advs.202508659

**Published:** 2025-11-05

**Authors:** Mattia Cenciarini, Andrea Uccelli, Francesca Mangili, Myriam Grunewald, Simone Bersini

**Affiliations:** ^1^ Regenerative Medicine Division Institute for Translational Research (IRT) Faculty of Biomedical Sciences Università della Svizzera italiana (USI) and Ente Ospedaliero Cantonale (EOC) Bellinzona 6500 Ticino Switzerland; ^2^ Euler Institute Faculty of Biomedical Sciences Università della Svizzera italiana Lugano 6900 Ticino Switzerland; ^3^ Istituto Dalle Molle di Studi sull'Intelligenza Artificiale (IDSIA) SUPSI Lugano 6900 Ticino Switzerland; ^4^ Faculty of Medicine The Hebrew University of Jerusalem Jerusalem 9190500 Israel; ^5^ Servizio di Ortopedia e Traumatologia Ente Ospedaliero Cantonale Lugano 6900 Ticino Switzerland

**Keywords:** longevity medicine, Organ‐on‐a‐Chip, vascular aging mechanisms, vascular health, vascular rejuvenation

## Abstract

Aging is a complex, multifaceted process affecting all organ systems, with vascular aging playing a central role in organismal health decline. Beyond its role in circulation, the vascular system acts as a dynamic interface between tissues, influencing countless physiological functions such as tissue regeneration and repair, immune responses, and metabolic balance. Importantly, age‐related vascular impairment—characterized by a peculiar set of endothelial aging hallmarks—exacerbates age‐related diseases (ARDs) such as cardiovascular disorders, neurodegeneration, chronic kidney disease, sarcopenia, and osteoporosis. This review combines basic concepts of angioscience and aging biology with translational interventions to devise clinical strategies promoting a functional rejuvenation of old and compromised blood vessels, fostering the prevention, delay or treatment of ARDs. Starting from the description of the cellular and molecular mechanisms driving vascular aging, a cutting‐edge perspective on the organ‐specific vascular impairment and its impact on tissue function is offered. Given the central role of the vasculature in aging, how targeting vascular aging through pharmacological, genetic, and lifestyle interventions holds promise for mitigating its systemic consequences and improving healthspan is discussed. Finally, how the combination of animal models (e.g., parabiosis) and novel microphysiological systems, coupled with multi‐omics and artificial intelligence‐driven analyses, is advancing the field toward the identification of strategies that promote vascular resilience and extend healthspan, addressing one of the most pressing biomedical challenges of a worldwide aging population is highlighted.

## Abstract 2

This review spotlights the emerging field of vascular aging, providing a comprehensive and critical overview of the molecular processes and clinical manifestations of vascular dysfunction during aging. By combining basic angioscience concepts with a critical analysis of innovative pre‐clinical aging models and lifestyle interventions, it offers a cutting‐edge perspective fostering the translation of geroscience discoveries into geromedicine.

## 1 Introduction

Throughout history, humanity has been captivated by the concept of aging, inspiring legends and myths that revolve around the pursuit of eternal youth and immortality. Tales such as the quest for a “Fountain of Youth” exemplify the profound enchantment with the mysteries of aging and the connected desire to overcome this biological limitation. This fascination has occasionally spurred ambitious real‐world endeavors, such as Bryan Johnson's Project Blueprint, aimed at pushing the boundaries of science in the fight against the aging process.^[^ ^1^ ^]^

Amidst legends, debatable anti‐aging routines, and the constantly expanding field of aging research, a fundamental question remains: why do we age? Over the past century, several theories have been proposed, though only a few have survived. Human aging is a multifaceted phenomenon whose complexity is due to the convergence of biological, genetic, and environmental factors contributing to the gradual decline in the function of organisms during adulthood. This process impacts primarily cells, then tissues and the whole organism, exhibiting a peculiar pattern of hallmarks that has been extensively described ^[^ ^2^ ^]^ and leading to the progressive and irreversible loss of function and regenerative capacity across all organ systems. Indeed, age constitutes the primary risk factor for a range of diseases, including neurodegenerative diseases, cardiovascular diseases (CVDs), metabolic disorders, and musculoskeletal conditions – collectively known as age‐related diseases (ARDs). These ARDs account for the majority of healthcare expenditure in developed countries, a challenge exacerbated by significant demographic shifts.^[^ ^3^, ^4^ ^]^ Consequently, the ultimate aim of aging research is to promote healthy aging by compressing the period of morbidity—the time during which individuals experience chronic illness or diminished health—thereby enabling them to maintain vitality and quality of life for longer.

Aging affects various systems in the body at different rates and paces, with some systems experiencing earlier and more rapid decline than others. Additionally, the aging of one system often accelerates the decline of others due to their interconnected nature. In this review, we will focus on vascular aging and its central role in the general aging process, exploring the models used to study it. Furthermore, we will discuss how artificial intelligence (AI) can enhance our understanding of vascular aging and its potential to improve our health outcomes in the context of an aging global population.

## 2 Vascular Aging: Foundations and Implications Across Organs

The vascular system plays a central role in supporting the physiological functions of every tissue and organ. Beyond merely transporting vital components throughout the body, blood vessels also provide angiocrine signals (i.e., factors secreted by endothelial cells, ECs) that are essential for maintaining parenchymal cell homeostasis, promoting tissue repair and supporting the survival and function of stem and progenitor cells within tissues.^[^ ^5^, ^6^, ^7^ ^]^ Dysfunction of the vascular system, affecting both macrovascular and microvascular structures, is a common feature in many ARDs due to this pervasive role. Impaired vascular functions have been directly linked to the onset of diseases, such as cardiovascular and cerebrovascular diseases and age‐related sarcopenia.^[^ ^8^, ^9^, ^10^ ^]^ Furthermore, experimental anti‐aging therapies like metformin, caloric restriction, and nicotinamide mononucleotide (NMN) have demonstrated the ability to enhance vascular function, underscoring its importance in longevity and health span.

Despite these clear connections, the precise impact of aging on vascular structures across organs remains poorly understood. Gaining deeper insights into age‐related modifications in blood vessels and their niches could uncover therapeutic targets, potentially influencing the vascular aging trajectory and mitigating its adverse effects on tissue function and regeneration.

### 2.1 Defining Vascular Aging

The process of vascular aging encompasses structural and functional changes that occur in blood vessels as an individual grows older.^[^ ^11^, ^12^ ^]^ These changes typically consist in alterations of the structure and function of arteries, veins, and capillaries, including endothelial dysfunction,^[^ ^13^ ^]^ arterial stiffening,^[^ ^14^ ^]^ capillary density reduction,^[^ ^15^ ^]^ and loss of organ‐specific endothelial structures.^[^ ^16^ ^]^ At the cellular level, the vascular aging concept is tied to the peculiar characteristics of ECs, as they are long‐lived, terminally differentiated cells that are quiescent but still retain the ability to proliferate when needed.^[^ ^17^ ^]^ Unlike other cell types that possess high turnover and are therefore replaced rapidly in the body (e.g., the majority of epithelial cells), ECs have been shown to survive for up to 80% of the average human lifespan,^[^ ^18^ ^]^ during which they are vulnerable to accumulating primary aging hallmarks and becoming dysfunctional.

Vascular aging can be categorized into two subtypes: i) healthy vascular aging, which is characterized by gradual changes that minimally affect vascular function and, overall, health; and ii) unhealthy vascular aging, which is instead defined by an accelerated and severe modification of the vasculature that, in turn, increases the risk of CVDs.^[^ ^19^, ^20^ ^]^ Another classification based on the degree of divergence between chronological age and the detected age‐related vascular alterations in an individual distinguishes between early, normal, and supernormal vascular aging.^[^ ^8^ ^]^ Early Vascular Aging (EVA) is characterized by an accelerated aging of blood vessels, with structural and functional changes occurring more rapidly than in normal aging.^[^ ^21^, ^22^ ^]^ Conversely, the recently identified phenotype known as SUPERNOrmal Vascular Aging (SUPERNOVA) is instead marked by unusually low arterial stiffness compared to the average for a person of the same age and sex.^[^ ^23^ ^]^ Bruno et al. quantified vascular age using a regression model that incorporated factors such as sex, smoking, height, cardiovascular parameters (e.g., heart rate, and systolic and diastolic blood pressure), total cholesterol, and glycemia. Once calculated the difference between chronological age and vascular age (Δ‐age), patients were classified as having EVA when their vascular age exceeded their chronological age by more than 5.7 years, while those with a vascular age 6.2 years or more younger than their chronological age were identified as SUPERNOVA patients.^[^ ^23^ ^]^ This approach has significant implications in clinical practice, as evaluating whether a patient belongs to the EVA, normal, or SUPERNOVA category might help in identifying a premature risk of coronary events.^[^ ^23^ ^]^

An additional distinction can be made based on the caliber of the affected vessels, resulting in the classification of vascular aging into microvascular and macrovascular aging. As suggested by their names, while the former focuses on significant structural and functional changes in small blood vessels (i.e., capillaries, venules, and arterioles), the latter pertains to the structural changes in larger vessels (i.e., arteries and veins) that contribute to cardiovascular risks. Of note, while macrovascular aging processes have been extensively studied and described in the scientific literature—particularly in relation to serious clinical outcomes such as atherosclerosis—the aging of the microvascular compartment, which constitutes the major vascular component in terms of both surface area and distribution across body tissues, remains relatively understudied. Since large vessels, particularly arteries, have a multi‐layered structure with different cell types and depend on a precisely organized elastic extracellular matrix (ECM), macrovascular aging research primarily focuses on alterations in these cell populations and the surrounding ECM. The resulting conditions and clinical implications include arterial stiffening, hypertension, accelerated atherosclerosis, and the formation and rupture of aneurysms. For additional comprehensive overviews, see also Donato et al. and Bulbul et al.^[^ ^24^, ^25^ ^]^

The interplay between microvascular and macrovascular aging is significant and underscores the importance of addressing both in aging individuals to mitigate the risk of CVDs. First, the molecular and cellular aging hallmarks affecting endothelial and vascular supportive cells not only have a pronounced impact on the microvasculature but also greatly influence macrovascular biology. Conversely, certain age‐related changes occurring in large vessels can result in conditions that exacerbate aging hallmarks within the microcirculation. An example of this extended interplay is exemplified by hypertension. For an overview of macrovascular aging mechanisms in CVDs, including the interplay with microvascular dysfunction in hypertension, see the box: Macrovascular Aging in CVDs.

**Macrovascular Aging in CVDs**

Macrovascular aging is a central contributor to CVD development. This box provides an overview of the major age‐related macrovascular conditions—including atherosclerosis, aneurysms, clonal hematopoiesis of indeterminate potential (CHIP), and hypertension—highlighting the cellular and molecular mechanisms that underlie their pathophysiology.

Within CVDs, ischemic heart disease remains the first cause of death, and it is frequently characterized by atherosclerotic disease of the coronary arteries.^[^ ^26^ ^]^ The initial step in atherosclerotic plaque formation is constituted by the deposition of lipoproteins, including oxidized low‐density lipoprotein (oxLDL), in the arterial wall.^[^ ^27^ ^]^ This accumulation induces ECs to produce inflammatory cytokines and chemoattractant chemokines (e.g., monocyte chemotactic protein‐1, MCP‐1), which recruit monocytes to the site of inflammation.^[^ ^28^, ^29^ ^]^ Once at the atherosclerotic plaque, monocytes first differentiate into macrophages and, upon oxLDL ingestion, transform into foam cells, thereby contributing to plaque destabilization.^[^ ^30^ ^]^ Finally, together with mast cells and dendritic cells, they expose oxLDL‐derived neo‐epitopes, which in turn trigger the adaptive immune system,^[^ ^292^ ^]^ such as CD4‐positive T‐helper cells, natural killer cells, and regulatory T‐cells.^[^ ^31^, ^32^, ^33^ ^]^ B‐cells further modulate disease progression: B‐1 cell‐secreted IgMs against oxLDL are athero‐protective, whereas B‐2 cell‐derived auto‐IgGs promote plaque formation.^[^ ^31^, ^34^ ^]^ A robust body of evidence has linked multiple cellular and molecular aging hallmarks to the onset and progression of atherosclerosis. For instance, DSB, increased oxidative DNA damage, sirtuin 1 (SIRT1) downregulation, increased pro‐inflammatory phenotypes, mitochondrial DNA alterations, decreased nitric oxide (NO) bioactivity, and disrupted proteostasis were all found to characterize multiple artery cell populations in atherosclerotic settings,^[^ ^35^, ^36^, ^93^ ^]^ including ECs, vascular smooth muscle cells (vSMCs) and fibroblasts.^[^ ^38^, ^39^ ^]^ Moreover, aging also impacts this immune compartment, leading to dysfunctional immunosenescent populations that exacerbate the atherosclerotic process.^[^ ^40^ ^]^

Aneurysms are pathological outward bulgings of arteries and coronary vessels, caused by a weakened spot in the vessel wall. They develop with aging as a result of changes that compromise vascular integrity and often remain asymptomatic until rupture, making them unpredictable and potentially life‐threatening. Although clinical data often show comorbidity between atherosclerosis and aneurysm, studies report conflicting correlations, and various theories have been proposed to explain causality.^[^ ^41^, ^42^ ^]^ Nevertheless, aneurysms and atherosclerosis share numerous molecular and cellular hallmarks that compromise vascular integrity. These include ECM remodeling and deposition (i.e., increased collagen deposition and elastin fragmentation), vSMC dysfunction, upregulation of inflammatory genes (e.g., Interleukin (IL)‐6 and MCP‐1), increased oxidative stress, and infiltration by innate and adaptive immune cells,^[^ ^19^, ^43^ ^]^ emphasizing the interconnected nature of these macrovascular diseases.

CHIP has emerged in the last decades as a fundamental risk factor for the development of CVDs, in particular in atherogenic settings. This condition arises when somatic mutations—which do not block hematopoietic differentiation—accumulate in the hematopoietic stem cell compartment over the individual's lifespan, leading to expanded clonal populations of mutated leukocytes in the blood. While this age‐related process does not directly lead to the development of leukemia per se, it predisposes to hematological malignancies and was found to correlate with increased cardiovascular risk.^[^ ^44^ ^]^ Mechanistic studies investigating the causative role of CHIP in CVDs have shown that certain mutated genes linked to clonal expansion and myeloid skewing (e.g., Tet methylcytosine dioxygenase 2, TET2) induce a pro‐inflammatory phenotype, with increased inflammasome activation and upregulation of cytokines such as IL‐1β and IL‐6. Inhibition of the inflammasome via NLR family pyrin domain containing 3 (NLRP3) blockers counteracts the pro‐atherosclerotic effects of TET2 mutations by reducing IL‐1β–mediated endothelial P‐selectin expression and subsequent macrophage recruitment,^[^ ^45^ ^]^ thus providing a molecular link between CHIP and atherosclerosis. Although CHIP‐related mutations have also been linked to heart failure, thrombosis, and myocardial injury, increased inflammation remains the primary mechanism by which CHIP exacerbates CVD pathophysiology in macrovascular aging.^[^ ^46^ ^]^

Hypertension is clinically defined as a systolic blood pressure ≥130 mmHg and/or diastolic blood pressure ≥80 mmHg, a condition particularly common in the elderly.^[^ ^47^ ^]^ The cardinal role of aging in its development is tied to the dysfunction of both cellular (e.g., ECs, vSMCs, and fibroblasts) and ECM components of the aging arteries, as well as environmental and systemic stressors (e.g., activation of the renin–angiotensin–aldosterone system, RAAS, or of the sympathetic nervous system). This results in thickening of the arterial wall, progressive loss of elasticity, increased arterial stiffness, and changes in vascular smooth muscle tone, ultimately giving rise to hypertension.^[^ ^48^, ^49^ ^]^ This condition has been primarily studied in the context of macrovascular aging. Nevertheless, once established, it also promotes and exacerbates aging‐related changes in the microvascular compartment, including aberrant signal transduction, impaired NO bioavailability, increased reactive oxygen species (ROS) production, inflammation, and fibrosis.^[^ ^50^ ^]^ This results in microvascular injury, rarefaction, and microbleeds, which drive extensive end‐organ damage and are particularly detrimental to the brain.^[^ ^51^, ^52^ ^]^ Conversely, although direct studies linking this phenomenon to aging are lacking, a mounting body of evidence suggests that microvascular rarefaction may contribute to hypertension via increased vascular resistance and impaired blood flow regulation. For example, human studies have shown that structural loss of microvasculature precedes the development of primary hypertension,^[^ ^53^ ^]^ while other studies propose that this rarefaction mediates the hypertensive effects of tyrosine kinase inhibitors used in cancer treatment.^[^ ^54^ ^]^ In conclusion, although current evidence only indirectly supports it, a causative link between age‐related microvascular dysfunction—including endothelial and vascular cell alterations—and hypertension remains to be established.^[^ ^55^, ^56^ ^]^

### 2.2 Primary Implications of Vascular Aging

**Figure 1:**
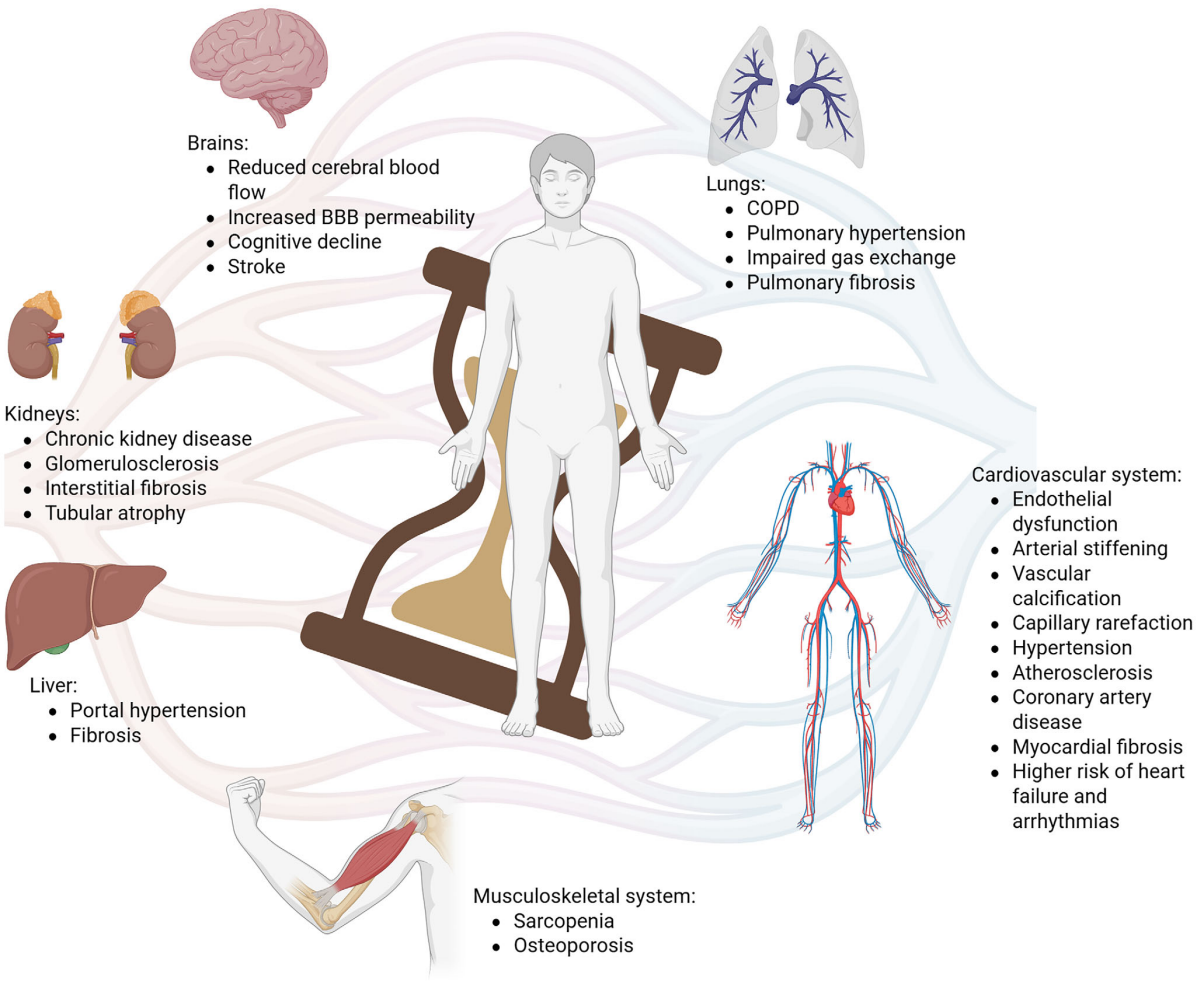
Main features and consequences of vascular aging. Endothelial dysfunction,^[^ ^13^ ^]^ arterial stiffening,^[^ ^14^ ^]^ vascular calcification,^[^ ^90^ ^]^ capillary rarefaction,^[^ ^15^ ^]^ and compromised barrier integrity ^[^ ^73^ ^]^ are key features of vascular aging. These players contribute to the development of important clinical consequences such as systemic hypertension,^[^ ^102^ ^]^ atherosclerosis,^[^ ^103^ ^]^ coronary artery disease (CAD), myocardial fibrosis,^[^ ^72^ ^]^ chronic kidney disease,^[^ ^98^ ^]^ glomerulosclerosis, interstitial fibrosis, and tubular atrophy,^[^ ^94^, ^96^ ^]^ sarcopenia,^[^ ^88^ ^]^ osteoporosis,^[^ ^90^ ^]^ chronic obstructive pulmonary disease (COPD), pulmonary hypertension and fibrosis,^[^ ^83^ ^]^ portal hypertension and liver fibrosis,^[^ ^93^ ^]^ cognitive decline,^[^ ^75^ ^]^ and stroke.^[^ ^104^ ^]^ Moreover, they increase the risk of cardiovascular events, such as heart failure and arrhythmias.^[^ ^72^ ^]^ Created in BioRender. Cenc, M. (2025) https://BioRender.com/ferxcdl.

The implications of vascular aging include various pathological conditions, affecting multiple organ systems throughout the body (**Figure** **1**). One of the most significant consequences is the progressive alteration of the dilation and constriction abilities of the vasculature.^[^ ^19^ ^]^ In particular, ECs play a crucial role in maintaining blood vessel homeostasis, regulating blood flow, and modulating inflammatory responses. However, as an individual ages, a decline of this endothelial functionality, termed endothelial dysfunction, is observed. A key manifestation of endothelial dysfunction is the altered production of vasoactive substances–such as NO, prostacyclin (PGI2), and endothelin‐1 ^[^ ^24^, ^57^, ^58^ ^]^–and the subsequent impairment of vasodilation and vasoconstriction abilities.^[^ ^57^ ^]^ Moreover, this impairment is further exacerbated by age‐related changes in systemic regulators, such as the RAAS,^[^ ^59^ ^]^ characterized by a higher production of angiotensin II (Ang II) due to the upregulation of the angiotensin‐converting enzyme (ACE) and increased expression of the Ang II type 1 receptors (AT1) in the vascular wall.^[^ ^57^ ^]^ The reduction of NO (which is a strong vasodilator) combined with increased Ang II (which is a potent vasoconstrictor) impairs vascular reactivity and elevates blood pressure, thus paving the ground for chronic hypertension and downstream CVDs.^[^ ^60^ ^]^

Another consequence of aging affecting multiple tissues is the alteration of the endothelium's barrier properties, defined as the semi‐permeable cellular barrier that regulates the transport of fluids, solutes, and cells between the circulation and surrounding tissues through tightly controlled paracellular and transcellular pathways. As further discussed in the following chapters, different endothelial aging processes integrate and contribute to impair the endothelial barrier function, resulting in deregulated trafficking of molecules and cells, and ultimately undermining the fine control over tissue homeostasis.

Further, aging is linked with the decline in the secretion of organ‐specific angiocrine factors, which are crucial for maintaining homeostasis and supporting stem cell niches, thus leading to a diminished regenerative capacity.^[^ ^6^, ^7^, ^17^, ^61^ ^]^ For instance, in the liver this age‐related loss of angiocrine signals, such as Wnt and bone morphogenic protein (BMP) signaling, has been linked to reduced liver size and impaired metabolic functions in aged mice.^[^ ^62^, ^63^ ^]^ Similarly, these alterations in the vascular niche in the bone marrow can result in decreased hematopoietic stem cell functionality.^[^ ^64^, ^65^, ^66^ ^]^ Moreover, the aging endothelium exhibits reduced expression of factors (e.g., stromal cell‐derived factor 1, SDF‐1), which are vital for tissue repair and regeneration, thereby contributing to a decline in the regenerative capacity of tissues.^[^ ^67^ ^]^

Aging individuals are often affected by a significant reduction in perfused microvessels and microvascular density. This so‐called microvascular rarefaction leads to the formation of large hypoperfused areas and is associated with various ARDs, including vascular cognitive impairment and heart failure.^[^ ^68^ ^]^ A primary consequence of this phenomenon is tissue hypoxia.^[^ ^69^ ^]^ Cells under hypoxic conditions react by upregulating the expression of proangiogenic factors, such as vascular endothelial growth factor (VEGF).^[^ ^69^, ^70^ ^]^ However, the imbalance between angiogenic and anti‐angiogenic mechanisms in aging tissues can impair the effect of this compensatory response,^[^ ^70^ ^]^ as further discussed below. The implications of microvascular rarefaction and tissue hypoxia extend beyond local tissue damage, affecting systemic health and contributing to conditions such as chronic kidney disease, heart failure, and neurodegenerative disorders.^[^ ^68^ ^]^

Altogether, these observations establish vascular aging as a central driver of multi‐organ functional decline and ARDs, rather than merely being a passive correlate of chronological age. This underscores the necessity of targeting vascular health in strategies aimed at preserving tissue function and extending healthspan.

### 2.3 Organ‐Specific Vascular Aging

ECs and blood vessels display distinct characteristics shaped by their unique microenvironments within various organs and tissues, reflecting specialized adaptations to meet diverse physiological demands.^[^ ^7^, ^71^ ^]^ This endothelial heterogeneity is also maintained in the aging process: not only aged ECs are phenotypically different from their younger counterparts, but also aging‐related changes to the structure and function of blood vessels vary amongst different organs.^[^ ^72^ ^]^ Chen et al. extensively explored this point.^[^ ^15^ ^]^ By analyzing both murine and human tissues, the authors observed that organs with low regenerative capacity (e.g., the kidney, brain, and lung) exhibit an age‐related reduction of vessel density and accentuated pericyte loss. In contrast, organs with moderate to high regeneration potential (e.g., the liver, skin, and gut) preserve vessel abundance and pericyte numbers during aging. The comparison between these two classes of tissues highlighted that ECs are characterized by an up‐regulation of inflammatory pathways in low‐ compared to high‐regenerative tissues, thus suggesting that ECs from tissues with high remodeling capacity may have evolved protective mechanisms against the phenomenon of aging‐related chronic low‐grade inflammation (often referred to as “inflammaging”) and its consequential damages.^[^ ^15^ ^]^

Furthermore, organ‐ and age‐dependent heterogeneity is not limited to differences in terms of vascular abundance. For example, aging in cerebral blood vessels often leads to a functional decline in terms of blood flow and impairment of the integrity of the blood‐brain barrier (BBB).^[^ ^73^ ^]^ This partly occurs as a consequence of a dysregulated communication between brain ECs and brain‐specific cellular populations, such as pericytes and astrocytes, which leads to increased BBB leakiness, neuroinflammation, and impaired nutrient delivery to brain cells,^[^ ^74^ ^]^ thus making individuals more susceptible to neurodegenerative pathologies,^[^ ^75^ ^]^ like dementia,^[^ ^76^ ^]^ Parkinson's, and Alzheimer's disease.^[^ ^13^, ^77^ ^]^ Transcriptomic analyses revealed an upregulation of genes associated with inflammation and senescence in aged brain capillary ECs compared to their younger counterpart, which results in tight junction impairment within the BBB. These changes at transcriptional level involve genes implicated in immune and cytokine signaling pathways (e.g., *Arhgap5*, *Pak2*, and *Cdkn1a*), in the maintenance of BBB integrity (e.g., *Ctnna1*, and *Iqgap1*), and in the alteration of energy metabolism (e.g., *Cox6c*, and *Hmgcs2*).^[^ ^78^ ^]^ Another age‐related phenomenon that characterizes brain vascular aging is the increase in the occurrence of cerebral microbleeds. These small, chronic hemorrhages–which are found in ≈18% of individuals aged 60–69 and in up to 38% of those over 80 ^[^ ^79^ ^]^–are closely linked to conditions such as cerebral amyloid angiopathy, in which the deposition of amyloid‐beta in the walls of cerebral blood vessels leads to an increased capillary fragility and a subsequent higher chance of microbleeds.^[^ ^80^, ^81^ ^]^ Moreover, the accumulation of amyloid‐beta increases the risk of micro‐thromboembolic events, as it promotes a pro‐inflammatory environment that facilitates clot formation.^[^ ^82^ ^]^ These changes in the cerebral microvasculature not only compromise the integrity of brain tissue, but also impair neurovascular coupling (NVC)—the dynamic regulation of blood flow in response to neuronal activity. Under normal conditions, NVC ensures that neuronal activation is matched by a rapid, proportional increase in local blood flow to meet metabolic demands (i.e., functional hyperemia). The neurovascular unit, comprising neurons, astrocytes, ECs, pericytes, and vSMCs, all embedded in the ECM, serves as the anatomical and functional framework for this process. In aging, several molecular and cellular hallmarks contribute to progressively disrupt this coordinated relationship, resulting in neurovascular uncoupling, a phenomenon characterized by a mismatch between neuronal activity and vascular response, with the detailed mechanisms described in other publications.^[^ ^52^ ^]^ This impairment has been identified as a key contributor to age‐related cognitive decline.

One of the most notable effects of aging on the lungs is the remodeling of the pulmonary vasculature, which can exacerbate conditions such as pulmonary hypertension and chronic obstructive pulmonary disease (COPD).^[^ ^83^ ^]^ Aging is indeed linked to a progressive deterioration in the structure and function of pulmonary circulation. Specifically, other than the already mentioned capillary rarefaction,^[^ ^15^ ^]^ there is an increase in pulmonary vascular stiffness, which can lead to elevated pulmonary arterial pressures.^[^ ^84^ ^]^ This is often accompanied by a reduction in pulmonary capillary blood volume, membrane diffusing capacity, and alveolar‐capillary surface area, thus affecting gas exchange capabilities.^[^ ^84^, ^85^ ^]^ Experiments performed on mice revealed an age‐dependent downregulation of low‐density lipoprotein receptor‐related protein 5 (LRP5, which stimulates angiogenesis) and other major angiogenic proteins (including Tie2 and VEGF receptor 2, VEGFR2) in ECs, suggesting an age‐related inhibition of angiogenesis in the lungs.^[^ ^83^ ^]^

Aging of cardiac blood vessels contributes to myocardial fibrosis, compromised heart contractility, and a higher risk of developing cardiovascular conditions, such as heart failure and arrhythmias.^[^ ^72^ ^]^ In this context, an important age‐dependent decline in the crosstalk between cardiac fibroblasts and cardiac ECs emerged through the comparison between the transcriptomes of cardiac cells obtained from either young or old mice. In particular, a higher expression of serine protease inhibitors (serpins) in aged fibroblasts showed anti‐angiogenic effects on cardiac ECs. Additionally, alongside diminished angiogenesis, aged fibroblasts were identified as responsible for impaired autophagy and proinflammatory activation in the aging cardiac endothelium.^[^ ^72^, ^86^ ^]^

In the context of the musculoskeletal system, age‐related changes in blood vessels can affect the delivery of nutrients and oxygen to muscles and bones, potentially contributing to conditions like sarcopenia and osteoporosis.^[^ ^87^ ^]^ Sarcopenia is characterized by the loss of skeletal muscle mass and strength, which can be a consequence of the endothelial dysfunction and capillary rarefaction observed in these organs during advanced vascular aging.^[^ ^15^, ^88^ ^]^ Osteoporosis, instead, is marked by excessive bone resorption, insufficient bone formation, and inadequate vascularization.^[^ ^89^ ^]^ Evidence supports the hypothesis of a close interplay between osteoporosis and vascular aging as age‐related changes in blood vessels (such as vascular calcification) were proven to contribute to the reduction of bone mineral density and strength, and individuals with osteoporosis often exhibit signs of early vascular aging.^[^ ^90^ ^]^ Additionally, the age‐dependent alteration of the vascular niches within the bone marrow was identified as a triggers for the loss of functional osteoprogenitors in the skeletal system.^[^ ^65^ ^]^

In the liver, aging is associated with changes in liver sinusoidal ECs, leading to the so‐called phenomenon of pseudocapillarization. This consists mainly in a morphological change of the hepatic endothelium, which results in the reduction of fenestrations, both in terms of number and diameter.^[^ ^91^ ^]^ This decline compromises the liver's ability to efficiently filter blood and metabolize substances, which can exacerbate conditions such as liver fibrosis and cirrhosis.^[^ ^92^, ^93^ ^]^

Renal aging is associated with a decline in glomerular filtration rate and renal blood flow, which are often attributed to alterations in the kidney microcirculation, including increased vascular resistance.^[^ ^94^, ^95^ ^]^ The structural alterations associated with vascular aging in these organs include glomerulosclerosis, interstitial fibrosis, and tubular atrophy, which collectively contribute to a decline in their function.^[^ ^94^, ^96^ ^]^ Furthermore, the renal vascular bed undergoes significant changes, including thickening of the basement membranes and increased stiffness of the renal arteries, which can lead to impaired renal hemodynamics, renal ischemia,^[^ ^97^ ^]^ and chronic kidney disease.^[^ ^98^ ^]^ To counteract these age‐related vascular alterations, recent studies have explored the systemic administration of SS‐31 (a mitochondria‐targeted tetrapeptide, also known as Elamipretide) in old animals, which was shown to limit glomerulosclerosis and improve podocyte and kidney EC integrity. Moreover, the treatment led to a higher glomerular EC density and an improved glomerular architecture.^[^ ^99^, ^100^, ^101^ ^]^

Altogether, the distinct vascular aging patterns observed in different tissues and organs highlight the importance of considering tissue‐specific mechanisms when studying the vascular aging process. In the next section, the role of the main cellular and molecular players involved in this process will be explored.

## 3 Endothelial Cellular and Molecular Mechanisms of Vascular Aging

**Figure 2:**
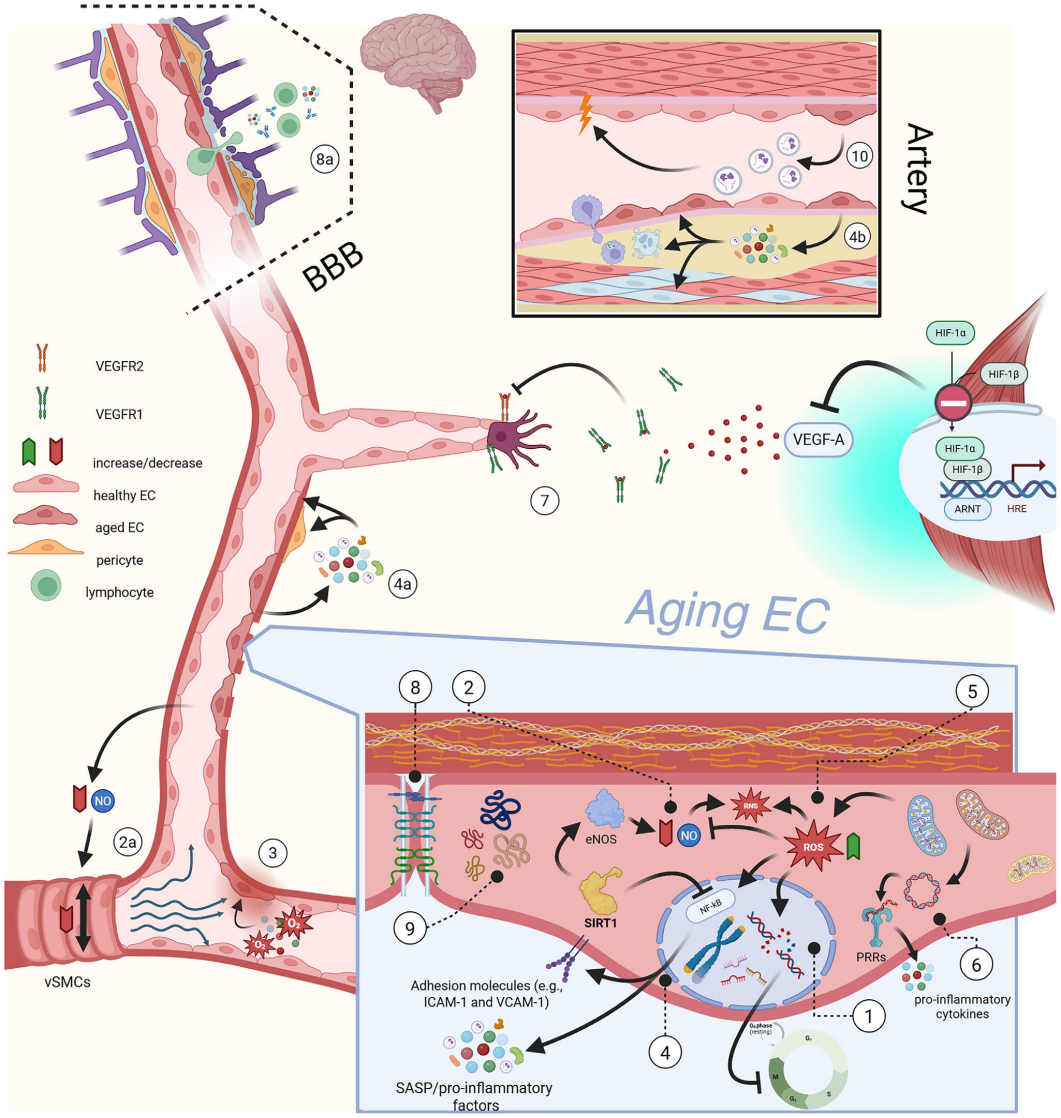
Graphical summary of the most relevant aging mechanisms impacting endothelial and vascular health.^[^ ^1^ ^]^ Genetic instability, telomere shortening, DNA damage, and epigenetic regulations are all primary hallmarks at the basis of EC aging and senescence.^[^ ^2^ ^]^ NO biology impairment and consequences at the level of ECs and (2a) vascular tissues.^[^ ^3^ ^]^ Environmental stressors such as oxidizing molecules and deleterious shear stress accelerate EC aging.^[^ ^4^ ^]^ The aging/senescent EC state is accompanied by a pro‐inflammatory SASP and upregulation of adhesion molecules. (4a) The SASP can enhance vascular senescence and leakiness or impact vascular mural cells in paracrine areas. (4b) SASP can also induce senescence and further phenotypic changes in cell types composing the vascular tissues in pathological settings (e.g., atherosclerosis).^[^ ^5^ ^]^ Oxidative and nitrative stresses are deeply intertwined with other endothelial molecular hallmarks as their causes and consequences.^[^ ^6^ ^]^ Mitochondrial dysfunction leads to increased ROS production and release of mtDNA, introducing additional chronic inflammatory stimuli.^[^ ^7^ ^]^ Aging is linked to reduced angiogenic capabilities. Proposed mechanisms to explain this dysfunction include a blunted response to hypoxia and diminished signal transduction in the target endothelium.^[^ ^8^ ^]^ Endothelial barrier impairment due loss of EC‐EC intercellular junction stability with aging underlies a vast number of ARDs. (8a) This dysfunction is detrimental for tissues such as the BBB and potentially destabilizes brain homeostasis.^[^ ^9^ ^]^ The disruption of proteostasis and accumulation of misfolded aggregates have been observed in several ARDs (e.g., Alzheimer's and Parkinson's disease) and CVDs.^[^ ^10^ ^]^ Intercellular communication through exosomes was observed to be significantly altered by aging. Created in BioRender. Cenc, M. (2025) https://BioRender.com/5huev2u.

The aging process of the macro‐ and micro‐vascular compartments is associated with both reversible and irreversible modifications of cellular components and molecular circuits (**Figure** **2**) ^[^ ^105^ ^]^. ECs are key players in vascular aging, regulating vascular function, integrity, and homeostasis.^[^ ^19^ ^]^ Endothelial dysfunction is responsible for impaired vasodilation, increased vascular leakiness, and altered angiogenic responses,^[^ ^19^ ^]^ while being characterized by increased oxidative stress, and low‐grade chronic inflammation. Here, we report the main endothelial cellular and molecular mechanisms that are altered in the aging organism, highlighting the interconnections between them and the overlap with known hallmarks of aging.

### 3.1 Genetic Instability, Telomere Shortening, and DNA Damage

Several lines of research have demonstrated that the accumulation of both nuclear and mitochondrial DNA (mtDNA) lesions, coupled with the loss of efficiency of the cellular DNA repair systems, accumulate along the cellular life span, hence compromising genomic integrity and resulting in molecular malfunctions related to pathological aging.^[^ ^2^ ^]^ ECs are particularly sensitive to DNA damage (due to their repair systems with limited efficiency) and show extended phenotypic and functional alterations as soon as their DNA is compromised.^[^ ^106^ ^]^ Endothelial aging and related vascular phenotypes (i.e., EC senescence, increased vascular stiffness, and hypertension) are directly caused by endothelial DNA damage and non‐functional repair mechanisms.^[^ ^107^ ^]^ Moreover, multiple biomarkers of double‐strand break (DSB) and increased oxidative DNA damage were associated with atherosclerotic plaques, typical pathogenetic structures found in cardiovascular ARDs.^[^ ^35^ ^]^

In somatic cells, including ECs, each cell division shortens the telomeres.^[^ ^108^ ^]^ It is well‐documented that ECs undergo age‐dependent replicative senescence and that this process is related to telomere shortening and uncapping.^[^ ^109^ ^]^ In this setting, following oxidative or replicative stress, telomeres function as cellular stress sensors and, once uncapped, initiate a persistent DNA damage response that contributes to the establishment of an endothelial senescent state.^[^ ^110^ ^]^ Accumulation of endothelial senescence is then at the basis of vascular dysfunction through multiple molecular mechanisms described in the subsequent paragraphs. Telomerase Reverse Transcriptase (TERT) is the catalytic subunit of an enzyme complex responsible for the synthesis of telomeric repeats and the regeneration of the telomeres. Although human TERT is active during early development and in stem cells, its activity is low in somatic cells, including ECs.^[^ ^108^ ^]^ The catalytic effect of TERT was found to counteract capillary rarefaction and other aging hallmarks, in the context of vascular aging, when p21‐positive ECs were induced to overexpress active or inactive TERT. Interestingly the inactive TERT was also shown to exert some positive effects, suggesting the existence of non‐canonical functions.^[^ ^111^ ^]^

Vasa et al. showed that in vitro EC replicative senescence is preceded by diminished TERT activity. Interestingly, supplementation with a NO donor delayed this loss of activity and subsequent development of senescence. Conversely, the inhibition of endogenous NO synthesis, exacerbated the decline in TERT functionality and EC senescence, suggesting a direct connection between NO activity and TERT functionality.^[^ ^112^ ^]^ Recent research employing endothelial‐specific telomerase deficient mouse models found that endothelial and vascular senescence are caused by the lack of TERT function in part through a telomere length‐independent mechanism compromising mitochondrial function.^[^ ^113^ ^]^ This is in line with previous publications showing a mitochondrial localization of TERT, coupled with mitigation of oxidative stress.^[^ ^114^ ^]^ This highlights how TERT functionality not only protects against telomere uncapping but also plays an important role in other endothelial molecular mechanisms affected by the aging process.

### 3.2 Epigenetic Modifications

Epigenetic alterations are central to EC aging biology. DNA methylation is a key factor in the regulation of biological processes in ECs, including inflammation, senescence, and apoptosis, and is reportedly compromised in the aging vasculature.^[^ ^115^ ^]^ For example, studies suggest that altered DNA methylation states of gene promoters pivotal for EC functionality (i.e., endothelial Nitric Oxide Synthase, eNOS; Activator Protein 1, AP‐1; and Superoxide Dismutase 2, SOD2) lead to aged phenotypes linked with vascular dysfunction.^[^ ^116^, ^117^ ^]^ Histone post‐translational modifications (PTMs) also play a pivotal role in regulating chromatin structure and gene expression. Several investigations have implicated a disrupted equilibrium between activating and repressive histone modifications in the aging vasculature, overall characterized by reduced repressive marks and partly overlapping with changes found in senescence.^[^ ^118^ ^]^ Histone acetyltransferase (HAT) and histone deacetylase (HDAC) are responsible for adding or removing acetyl groups from histones, thereby altering DNA accessibility. HDAC6, HDAC7 and HDAC9 were found to be involved in angiogenic processes such as EC migration and tube formation, and other activities reduced in the aging vasculature.^[^ ^119^, ^120^ ^]^ Sirtuins are HDACs extensively studied for their role in regulating cellular aging processes in humans.^[^ ^121^ ^]^ Among them, SIRT1, SIRT3, and SIRT6 were all found to regulate important endothelial functions compromised during aging, such as reduction of oxidative stress and inflammation in ECs, increasing the expression of eNOS, and exerting anti‐atherogenic effects.^[^ ^19^ ^]^ Importantly, SIRT1 expression in arterial ECs is reduced with aging,^[^ ^37^, ^122^ ^]^ thus contributing to the pathogenesis of atherosclerosis.^[^ ^93^ ^]^ Similarly, the downregulation of SIRT6 is one of the main drivers of endothelial phenotype alteration in this CVD, and results in an increased vascular leakiness and expression of adhesion molecules, such as vascular cell adhesion molecule‐1 (VCAM‐1).^[^ ^123^ ^]^ Supporting these findings, recent studies have shown that SIRT6 overexpression in mice can delay critical aspects of the aging process,^[^ ^124^ ^]^ protecting ECs from DNA damage, telomere dysfunction, and senescence.^[^ ^125^ ^]^ Collectively, these observations establish SIRT6 as a key regulator of EC aging, highlighting its potential as a promising therapeutic target for addressing ARDs and mitigating vascular aging.

The role of non‐coding RNA in the regulation of endothelial aging biology is also emerging. These molecules can be small non‐coding RNAs (< 200 nucleotides) or long non‐coding RNAs (> 200 nucleotides), with micro‐RNAs (miRNAs) being the most studied in the context of aging. Several miRNAs are involved in the regulation of processes strictly related to endothelial homeostasis ^[^ ^126^ ^]^ and aging, such as senescence and inflammation.^[^ ^127^, ^128^ ^]^ Examples are miR‐21, miR‐181b, miR‐34a, and the miR‐17‐92 cluster.^[^ ^129^, ^130^ ^]^ Moreover, some miRNAs can exert detrimental effects on different cell types, such as circulating endothelial progenitor cells (EPCs) and vSMCs, thereby compromising the vascular microenvironment and regenerative potential, contributing to the onset of CVDs.^[^ ^131^, ^132^ ^]^ Multiple connections between miRNAs and endothelial aging hallmarks have been observed. For instance, miR‐221, miR‐222, and miR‐34a were shown to suppress eNOS and SIRT1 activity,^[^ ^130^, ^132^, ^133^ ^]^ while the increased or reduced expression of miR‐21, miR‐92a, and miR‐126 was found to promote endothelial senescence, apoptosis, and inflammation.^[^ ^133^ ^]^ Interestingly, miRNAs involved in the aging process were also found to be released in the extracellular environment and in the systemic circulation, thus representing an important set of biomarkers.^[^ ^134^ ^]^

### 3.3 NO Biology and Vasoactive Control Impairment

NO is considered a master molecular regulator determining longevity and health span.^[^ ^135^ ^]^ Indeed, one of the main consequences of a non‐functional endothelium is a reduced bioavailability of NO, which is essential for the maintenance of vascular health.^[^ ^19^ ^]^ This is partly due to alterations in the functionality of eNOS in aged ECs: with aging, not only is this enzyme downregulated, but also its activity is decreased.^[^ ^136^ ^]^ This drop in the catalytic performance occurs as a result of a shortage of substrates and cofactors, changes in terms of protein‐protein interactions, and inhibitory PTMs, such as acylation, nitrosylation, O‐GlcNAcylation, and phosphorylation.^[^ ^137^, ^138^ ^]^ It is now established that the age‐related NO biology impairment and oxidative overload of the endothelial compartment are deeply interconnected. In fact, dysfunctional nitric oxide synthase (NOS) enzymes establish a vicious cycle characterized by reagent/cofactor‐depleted NOS reactions (i.e., NOS uncoupling) that generate superoxide in aged ECs already carrying increased ROS content. Superoxide then oxidizes the NOS cofactor tetrahydrobiopterin (BH4), further exacerbating the diminished NO production. This uncoupling is aggravated by increased downstream NO consumption, as NO reacts with superoxide to produce peroxynitrite (ONOO^−^).^[^ ^139^ ^]^ Decreasing eNOS and NO levels have profound deleterious effects on the vascular system, such as dysfunctional neovascularization following reduced mobilization of EPCs.^[^ ^140^ ^]^ In fact, impaired biogenesis and signaling of NO is known to contribute to cardiovascular risk associated with hypertension, hyperlipidemia, and diabetes,^[^ ^141^ ^]^ while the dietary supplementation of NO precursors can maintain cardiovascular health, blood flow, and cognitive performance.^[^ ^135^ ^]^ Moreover, superoxide and ONOO^−^ increase oxidative endothelial stress and establish a pro‐inflammatory state that primes leukocyte adhesion and sets the stage for CVD onset and progression.^[^ ^142^ ^]^ Finally, the age‐related disruption of NO dynamics in ECs is deeply interconnected with other aging hallmarks such as increased vascular leakiness and senescence, demonstrating—as discussed further below—that NO could be proposed as a pleiotropic factor in vascular aging.

Although NO is the most studied vasoactive molecule impaired in aging‐driven endothelial and microvascular dysfunction, other factors contribute to the loss of vasoactive control. The vasculoprotective factor PGI2 is a cyclooxygenase (COX)–1 and –2–derived eicosanoid primarily produced by ECs through its terminal specific enzyme prostacyclin synthase (PGIS). It acts as a potent autocrine/paracrine vasodilator and inhibitor of platelet aggregation, counterbalancing the effects of vasoconstrictor and pro‐thrombotic eicosanoids such as thromboxane. Aging shifts the eicosanoid profile toward proconstriction, correlating with hypertension, atherosclerosis progression, and thrombotic risk. Human blood PGI2 levels decline with age, and human ECs show reduced PGI_2_ production when serially passaged.^[^ ^143^ ^]^ Importantly, Nicolson et al. demonstrated that in aged humans the blunted vasodilatory effects of exogenous PGI2 are due to a decreased contribution of endothelial‐derived NO,^[^ ^144^ ^]^ potentially explaining why PGI2 receptor downregulation was not observed in aged animals.^[^ ^145^ ^]^ This finding raises important questions about whether interventions aimed at restoring PGI2 levels can effectively reverse this impairment. Hydrogen sulfide (H_2_S), a gasotransmitter produced from L‐cysteine by cystathionine‐γ‐lyase (CSE) in the cardiovascular system, exerts vascular protective and vasoactive effects and has been shown to ameliorate endothelial dysfunction by modulating inflammation in senescent ECs ^[^ ^146^ ^]^. However, CSE deficiency and reduced H_2_S levels with age impair endothelium‐dependent vasodilation and have been linked to various ARDs.^[^ ^147^ ^]^ Although microvascular studies are limited, inhibition of ferroptosis, enhanced CSE expression, and reduced lipid peroxidation have been identified as protective mechanisms of H_2_S supplementation in the aging endothelium.^[^ ^148^ ^]^ Furthermore, endothelial CSE knockout causes premature vascular aging, reversed by H_2_S through SIRT2‐mediated inflammasome modulation, reducing inflammatory cytokines and improving vascular function.^[^ ^149^ ^]^ These results align with other findings indicating that exogenous H_2_S promotes SIRT1‐mediated positive effects on endothelial migration, proliferation, and sprouting in vitro and—when combined with a nicotinamide adenine dinucleotide (NAD+) precursor—ameliorates capillary density in aged mice while protecting against oxidative damage.^[^ ^150^ ^]^

Epoxyeicosatrienoic acids (EETs) are another class of vasoactive compounds that act as endothelium‐derived hyperpolarizing factors and arise from cytochrome P450–mediated metabolism of arachidonic acid.^[^ ^151^ ^]^ EETs possess vasodilatory, anti‐inflammatory, and overall cardiovascular protective effects, and have been shown to promote angiogenesis and enhance the function of EPCs.^[^ ^152^ ^]^ Aging was shown to impair the availability of EETs, as evidenced by progressively reduced EET levels during heat‐induced vasodilation and diminished EET‐mediated dilatory responses in human peripheral conduit arteries of aged individuals.^[^ ^153^ ^]^ Conversely, the action of soluble epoxide hydrolase (sEH)—which rapidly converts and inactivates EETs—contributes to the onset and progression of CVDs and age‐related endothelial dysfunction, supported by observations that sEH expression is elevated in the microvascular endothelium of postmortem human brain tissue from individuals with dementia.^[^ ^152^, ^154^ ^]^

An intriguing link with oxidative stress emerges when considering another key vasoactive modulator in the endothelium: hydrogen peroxide (H_2_O_2_). Produced by dismutation of superoxide generated during mitochondrial respiration, as well as by membrane‐associated oxidases and uncoupling of NOS in vascular cells, H_2_O_2_ at low concentrations acts as an endothelium‐derived hyperpolarizing factor,^[^ ^155^, ^156^ ^]^ in particular when the bioavailability of NO is reduced. However, the function of H_2_O_2_ rapidly becomes detrimental in vascular aging contexts. As further discussed in this manuscript, although ECs show increasing resistance to these oxidative species during aging,^[^ ^156^ ^]^ the antioxidant defense system progressively fails to counterbalance escalating oxidative stress, resulting in excessive ROS, irreversible cellular damage, and downstream impairment of endothelial function.

### 3.4 Senescence Associated Secretory Phenotype (SASP)

Genomic instability, epigenetic alterations, sublethal DNA damage, and telomere shortening are at the basis of endothelial senescence. ECs are among the primary cell types to undergo senescence as individuals age, and the accumulation of senescent ECs in multiple vascular beds is linked to an increased risk of developing CVDs,^[^ ^157^ ^]^ including stroke, atherosclerosis, vascular dementia, peripheral artery disease (PAD), and erectile dysfunction.^[^ ^158^ ^]^ This state can be triggered in ECs by multiple factors, including their location in the vascular tree, the surrounding hemodynamic environment, and the presence of peculiar shear stress patterns.^[^ ^159^ ^]^ Moreover, blood itself exposes ECs to a variety of substances, including potential harmful stimuli, thus making them prone to chronic injury and senescence. Lastly, oxidative stress and inflammation drive endothelial senescence through DNA damage and activation of DNA damage response pathways.^[^ ^160^, ^161^, ^162^ ^]^ It must be remembered that, although frequently and mistakenly used interchangeably, aging and senescence are very distinct biological states/processes, with the latter being only one of the multiple hallmarks of aging. As extensively reviewed by López‐Otín et al., senescence is a cell state and an “antagonistic” aging hallmark. This is in line with the theory of antagonistic pleiotropy: while senescence can be functionally beneficial in the early stages of an organism's lifespan, it gradually assumes disruptive and dysfunctional features as the organism ages and the pool of senescent cells in different tissues increases, also due to the accumulation of primary aging hallmarks.^[^ ^2^ ^]^

Senescent ECs display impaired response to shear stress, flattened‐enlarged morphology, and increased expression of senescence markers (e.g., senescence associated‐β‐galactosidase, SA‐β‐Gal; cell cycle inhibitors, such as p16 and p21; and adhesion molecules, such as VCAM‐1).^[^ ^163^, ^164^ ^]^ Moreover, they exhibit the so‐called SASP, which is characterized by an altered and detrimental shift in the secretome with an increased secretion of pro‐inflammatory cytokines (e.g., tumor necrosis factor alpha, TNF‐α; interleukin 6, IL‐6; and plasminogen activator inhibitor 1, PAI‐1) and chemokines (e.g., C‐X‐C Motif Chemokine Ligand 1, CXCL1; and C‐C Motif Chemokine Ligand 2, CCL2), thus supporting inflammaging.^[^ ^164^, ^165^, ^166^ ^]^ By increasing vessel inflammation, these components further boost endothelial senescence, finally promoting vascular damage through decreased barrier function and reduced cell proliferation and motility.^[^ ^19^, ^157^ ^]^ Importantly, the accumulation of senescent ECs—together with senescent vSMCs and senescent macrophages—in the intima layer is a hallmark of atherosclerosis. By secreting SASP proteins, these cells promote plaque growth and rupture, leading to acute cardiovascular events such as myocardial infarction or stroke.^[^ ^104^ ^]^ Moreover, the increasing endothelial senescent population is known to reduce the expression of adherens junction proteins in surrounding non‐senescent ECs, thus affecting vascular barrier function and the ability of blood vessels to undergo vascular remodeling.^[^ ^167^ ^]^

In this scenario, evidence suggests that NO could be the main vasoactive protective molecule in the prevention of endothelial senescence.^[^ ^168^ ^]^ However, its bioavailability is frequently impaired in the aging vessels due to several malfunctioning pathways and to the action of oxidative stress. Moreover, senescent ECs have an impaired production of NO, which further reinforces this detrimental effect.^[^ ^18^ ^]^ This strengthens the concept that aging undermines endothelial biology by synergistically disrupting cellular and molecular pathways through mechanisms that mutually reinforce each other.

### 3.5 Oxidative and Nitrative Stress

Another hallmark of vascular aging is oxidative stress, a condition of imbalance between the cellular antioxidant defense system (whose functionality declines with aging) and the production of ROS and reactive nitrogen species (RNS). ROS are generated through different processes in mitochondria, lysosomes, peroxisomes, and endoplasmic reticulum.^[^ ^169^ ^]^ To counteract the detrimental effects of these molecules, the endothelial compartment relies on the erythroid 2‐related factor 2 (Nrf2)‐driven antioxidant defense pathway. However, the activity of Nrf2 decreases with aging,^[^ ^167^ ^]^ thus allowing ROS‐mediated damage to cellular components (including DNA, lipids, and proteins).^[^ ^142^ ^]^ In addition, oxidative stress is responsible for accelerated telomere loss, impaired endothelium‐dependent vasodilation, induction of the SASP, and shift in ECM production.^[^ ^18^, ^170^ ^]^ Abundant ROS can also cause the constant activation of cellular sensors (such as NF‐kB and AP‐1), thus promoting a low‐level, chronic inflammatory vascular phenotype accompanied by the release of pro‐inflammatory cytokines (e.g., IL‐1β, IL‐6, and IL‐8) and overexpression of adhesion molecules.^[^ ^18^, ^142^ ^]^

The increased endothelial oxidative stress has been frequently reported as both the causative factor and consequence of the imbalance between vasodilators and vasoconstrictors (e.g., eicosanoids and NO), a condition strictly related to endothelial dysfunction that precedes by years the clinical manifestation of CVDs.^[^ ^142^, ^171^ ^]^ In the context of a dysfunctional EC with critical NO bioavailability and consistent oxidative stress, the rapid reaction between NO and dysregulated ROS further quenches the NO bioactivity (consuming the available NO) and generates toxic RNS (e.g., ONOO^−^). These RNS contribute to cellular damage and accelerated aging through multiple mechanisms: protein nitration, disruption of signaling cascades, and activation of poly(ADP‐ribose) polymerase (PARP‐1). The resulting PARP‐1 activation leads to NAD+ depletion in ECs, which subsequently impairs the function of SIRT1.^[^ ^172^ ^]^ Importantly, nitrative stress has been observed in the aging endothelium and promotes endothelial dysfunction,^[^ ^173^ ^]^ as covered in detail in other reviews.^[^ ^173^, ^174^ ^]^

### 3.6 (Chronic) Inflammation

Endothelial oxidative stress and vessel inflammation are strictly connected and are key events in the development of ARDs.^[^ ^175^ ^]^ It has been shown that the aging organism is defined by inflammaging, a condition characterized by an increased basal expression of inflammatory markers such as IL‐1, IL‐8, and C‐reactive protein (CRP).^[^ ^176^ ^]^ It is now becoming clear that the oxidative stress‐driven activation of redox‐sensitive transcriptional factors such as AP‐1 and NF‐κB is at the basis of vascular inflammation and causes the increased expression of inflammatory cytokines (e.g., TNF‐α), interleukins (e.g., IL‐1 and IL‐6), chemokines, adhesion molecules (e.g., Intercellular Adhesion Molecule 1, ICAM; and VCAM), and enzymes producing vasoactive compounds (e.g., inducible NOS, iNOS; and COX‐2),^[^ ^177^ ^]^ and potentially contributing to a dysregulated vascular tone. Furthermore, inflammation of the endothelium is affected by pleiotropic factors, including sirtuins. Given their NAD+‐dependent activity, sirtuins are directly linked to the metabolic and redox state of the cell and their changes during aging. Among the most studied, SIRT1 was found to greatly suppress the activity of NF‐κB and regulate eNOS activity, providing an additional connection between oxidative stress, inflammation, and aging in the vasculature.^[^ ^178^, ^179^ ^]^ Moreover, an important repressive function of SIRT3 and SIRT6 on Hypoxia‐Inducible Factor 1‐alpha (HIF‐1α) highlights the importance of these sirtuins in the regulation of hypoxia‐induced inflammation and angiogenesis during endothelial aging.^[^ ^171^ ^]^

As previously discussed, the chronic inflammatory state of the aged vasculature is also a direct consequence of other cellular hallmarks of aging. Pro‐inflammatory cytokines contained in the SASP and released by senescent cells accumulate in the entire organism and could contribute to the establishment of a pro‐inflammatory systemic environment in the vascular system.^[^ ^180^ ^]^ Furthermore, senescent cells release debris, including a wide range of damage‐associated molecular patterns (DAMPs) that stimulate cell surface and intracellular pattern recognition receptors (PRRs), which then trigger pro‐inflammatory responses.^[^ ^181^ ^]^

Finally, being the endothelium an interface between tissues and blood‐circulating immune cells, it is not surprising that inflamed activated ECs are more prone to the adhesion and recruitment of immune cells such as monocytes, lymphocytes, and neutrophils.^[^ ^182^, ^183^ ^]^

### 3.7 Mitochondrial Dysfunction

Mitochondria are one important source of ROS (mtROS) in ECs, and their dysfunction contributes to the increasing oxidative stress in the aging vasculature.^[^ ^184^ ^]^ In fact, mtROS generated from the electron transport chain increasingly accumulate during aging, contributing to age‐related cardiovascular damage.^[^ ^185^ ^]^ Furthermore, antioxidant enzymes, including SOD2, mitochondrial aldehyde dehydrogenase (ALDH‐2), mitochondrial catalase, and glutathione (GSH) have diminished mtROS inactivation ability in aged individuals, thus negatively affecting endothelial functionality.^[^ ^185^ ^]^ This mtROS accumulation has, in turn, some serious downstream effects, such as increasing the already high mutation rate of mtDNA.^[^ ^186^ ^]^

Aging is correlated with increasing levels of mtDNA in cytoplasmic compartments and in blood. As mtDNA originates from eubacteria, it contains DAMPs that can be readily recognized as foreign entities by PRRs in the cytoplasm. This recognition process is in part exerted by cyclic guanosine monophosphate adenosine monophosphate synthase (cGAS)‐stimulator of interferon genes (STING) pathway, Toll‐like receptor (TLR), and Nod‐like receptor family pyrin domain containing 3 (NLRP3) inflammasome, and leads to the initiation of low‐grade inflammation and innate immune response with production of cytokines.^[^ ^187^, ^188^ ^]^ Indeed, this constitutes an additional potential mechanism connecting aging‐driven mitochondrial dysfunction and the chronic inflammation of the aging vasculature.

Among the mitochondrial processes affecting cell function and aging are fusion and fission. These mechanisms are important for maintaining the quality of the mitochondrial population and replacing damaged mitochondrial components.^[^ ^189^ ^]^ In senescent ECs, both mechanisms decline and are imbalanced, thus promoting mtROS production and impaired endothelial function.^[^ ^190^, ^191^ ^]^ Mitophagy, a selective autophagy that scavenges mitochondria, also has a protective effect against age‐related illnesses and slows down the process of premature vascular aging. This is achieved through the removal of unnecessary or dysfunctional mitochondria and the elimination of toxic metabolites. However, with aging the efficiency of this process is reduced, thus positively contributing to the aging process itself.^[^ ^185^ ^]^

### 3.8 Angiogenesis Impairment

As a response to ischemic insults and physiological needs, the vascular system initiates angiogenesis to restore optimal blood perfusion and prevent tissue damage.^[^ ^120^ ^]^ Nevertheless, aging is linked to reduced angiogenic abilities, leading to higher rates of cardiovascular complications and a decreased potential for tissue preservation and regeneration.^[^ ^192^ ^]^ In fact, elderly patients have reported reduced capillary density and impaired angiogenesis in response to ischemia or infarction while experiencing increased incidence of stroke, claudication, and myocardial ischemia.^[^ ^120^ ^]^

Various stimuli, including hypoxia, affect the process of angiogenesis through multiple mechanisms. One key factor is the regulation of secreted angiogenic peptides, such as VEGF, whose transcription is regulated by specific transcription factors able to sense the concentration of oxygen and nutrients in the cell. The most characterized pathway is the HIF‐1α pathway, ^[^ ^102^ ^]^ and a defective expression or activation of HIF‐1α protein was detected in studies of aged animal models.^[^ ^193^, ^194^ ^]^ Intriguingly, multiple types of interaction between Sirtuins and different HIF isoforms were also reported, suggesting that the formers might have a role in controlling endothelial response to hypoxia.^[^ ^195^ ^]^

Another molecular mechanism potentially explaining the loss of angiogenic potential and microvascular rarefaction observed in older individuals was provided by Grunewald et al. The group found that VEGF signaling is impaired in aging mice and that this loss was associated with an increased production of soluble VEGF receptor 1 (sVEGFR1), a VEGF receptor that binds to VEGF, but does not initiate downstream signal transduction, hence effectively functioning as a VEGF decoy. Furthermore, when comparing a transgenic mouse model designed to elevate systemic VEGF levels with an age‐matched control, the group was able to demonstrate protection against age‐related capillary rarefaction, reduced tissue oxygenation and diminished aging hallmarks such as mitochondrial dysfunction, endothelial cell senescence, and inflammaging.^[^ ^70^ ^]^ Of note, while VEGF levels were found to significantly change (i.e., decrease with age in only two of the five organs examined in this study), a large‐scale screening performed by the Wyss‐Coray group on human plasma from young and old donors revealed a trend toward upregulation of VEGF plasma levels with increasing age,^[^ ^196^ ^]^ potentially suggesting that age‐related VEGF dynamics may vary depending on the tissue, experimental context, or species.

The impairment of angiogenesis is also linked with other hallmarks of vascular aging. For instance, angiogenesis relies on the ability of ECs to replicate and migrate. As already discussed, vascular aging is frequently associated with EC senescence and therefore blunted replicative capacity. Moreover, existing evidence indicates a clear connection between endothelial telomere attrition (which is at the basis of replicative senescence in human cells), the impairment of angiogenesis in the aging vasculature, and the onset of CVDs such as atherosclerosis.^[^ ^120^ ^]^ Finally, as frequently reported in literature, vascular aging is connected to a severe reduction in the bioavailability of NO, a molecule that plays a vital role in mediating the angiogenic effects of VEGF and in the mobilization and effectiveness of EPCs.^[^ ^197^ ^]^ In addition to the already mentioned involvement in oxidative stress, the reduced expression and activation of eNOS in aged ECs can lead to a decreased production of NO ^[^ ^198^ ^]^ and to an impaired angiogenic potential.

### 3.9 Vascular Barrier Function Impairment

Because of the loss of EC‐EC intercellular junction stability with aging,^[^ ^199^ ^]^ the progressively impaired endothelial barrier function is now considered one major pathogenic mechanism underlying a vast number of ARDs.^[^ ^200^ ^]^ This phenomenon is particularly detrimental where a complex structural barrier (e.g., BBB) preserves tissue functions by finely controlling which solutes and cell populations can migrate from blood to tissues and vice versa. The likelihood of microvascular dysfunction increases with the natural process of aging and, even in those individuals who are overall healthy, an older age is correlated with a higher rate of BBB leakage in both white and grey matter. This fact indicates that BBB disruption is a common phenomenon during the aging process.^[^ ^201^ ^]^

Numerous adhesion molecules play a role in regulating and maintaining the strength and permeability of junctions between ECs. Adherens junctions are responsible for mediating structural cell‐to‐cell adhesion between neighboring cells and for controlling permeability of large plasma components. Tight junctions are additionally present where a strict compartmentalization between blood and the surrounding tissues is required.^[^ ^202^ ^]^ Among the proteins constituting cell‐cell adherens junctions, vascular endothelial (VE)‐cadherin is essential and links neighboring ECs by using calcium‐dependent homophilic binding in its external domain, while its internal domain interacts with the actin cytoskeleton. Tight junctions are instead mainly built using claudins, such as Claudin‐5 (Cldn‐5). Moreover, Occludin, Nestin, junctional adhesion molecules (JAMs), and platelet endothelial cell adhesion molecule (PECAM)‐1, are also important for the maintenance of functional junctions and endothelial functions in the organism.^[^ ^203^ ^]^ As years go by, these intercellular connections undergo a process of progressive disruption, resulting in endothelial hyperpermeability.^[^ ^204^ ^]^

Oxidative stress, senescence, and inflammaging are at the basis of a profound change in the expression pattern and functionality of endothelial junctions, potentially resulting in altered paracellular leakiness.^[^ ^205^ ^]^ First, oxidative stress and senescence are responsible for the release of several pro‐inflammatory factors such as histamine, thrombin, VEGF, and interleukins. The endothelial wall reacts promptly to some inflammatory stimuli (e.g., histamine or IL‐1) by increasing its permeability and promoting edema to cope with tissue insult. Among these molecules, VEGF (frequently contained in the SASP) is able to alter EC junction composition, thereby causing extensive vascular leakage during angiogenesis.^[^ ^206^, ^207^ ^]^ Moreover, increased oxidative stress was demonstrated to stimulate endothelial dysfunction by triggering matrix metalloproteinase (MMP)‐ and γ‐secretase‐mediated endoproteolysis of VE‐cadherin.^[^ ^208^ ^]^ Finally, the declining bioavailability of NO during aging severely affects endothelial junction integrity.^[^ ^209^ ^]^

Another important factor is the increase with age of advanced glycation end products (AGEs).^[^ ^210^ ^]^ AGEs are known for inducing the phosphorylation of β‐catenin at specific residues, which results in the dissociation of VE‐cadherin/β‐catenin complex and in the subsequent impairment of adherens junctions. Moreover, AGEs‐mediated β‐catenin nuclear translocation promotes the transcription of A Disintegrin and Metalloproteinase domain‐containing protein 10 (ADAM10) which, in turns, mediates VE‐cadherin shedding and leads to further impairment of EC‐EC intercellular connections.^[^ ^211^ ^]^

An example of how aging affects EC barrier function was reported by Yang et al. and involves the mechanism that allows plasma uptake by the BBB and its impairment with age.^[^ ^73^ ^]^ In fact, the authors observed that the vast majority of genes downregulated with age correlated with plasma uptake–such as transferrin receptor (*Tfrc*), clathrins (e.g., *Clta*, *Cltb*, and *Cltc*) and clathrin adaptors (e.g., *ap2a1*, *Picalm*, and *Epn1*)–suggesting a defective receptor‐mediated transcytosis in old mice. Differently, the expression of genes involved in caveolae formation (e.g., *Cav1*, *Cav2*, and *Cavin1*) were upregulated with age, indicating that aging promotes a shift from ligand‐specific, receptor‐mediated transcytosis to nonspecific caveolae‐mediated transcytosis. This shift changes the identity of the proteins allowed to transmigrate from the plasma to the brain parenchyma. This way, neurotoxic proteins such as albumin, fibrinogen and autoantibodies have access to the aged parenchyma where they can trigger neuroinflammation.^[^ ^73^ ^]^

### 3.10 Exosomes and Exosomal miRNAs in Vascular Aging

Altered intercellular communication is considered a major hallmark of aging.^[^ ^2^ ^]^ Exosomes, which are small extracellular vesicles (EV), are released by nearly all eukaryotic cells. They serve as carriers of distinctive biological information (e.g., proteins, small molecules, and genetic information) from donor cells, functioning to regulate the local cellular environment, facilitate signal transfer among neighboring or distant cells, and influence the expression of specific genes in recipient cells.^[^ ^212^ ^]^ A proof of concept regarding the impact of exosomes on vascular aging was given by Feng et al. who showed that these vesicles from induced pluripotent stem cell (iPSC)‐derived mesenchymal stem cells were able to mitigate arterial aging in old mice and cultured human ECs, ameliorating endothelial dysfunction, arterial stiffness, and hypertension, likely through regulation of the SIRT1‐AMP‐activated protein kinase (AMPK)‐eNOS pathway, MMPs, and elastase.^[^ ^213^ ^]^

Enclosed within EVs, circulating miRNAs are shielded from degradation by RNase present in body fluids, thereby enabling their involvement in intercellular communication through the transfer of genetic material.^[^ ^214^ ^]^ A large body of evidence suggests that exosomal miRNAs may have an impact on endothelial dysfunction and vascular cell senescence. In this regard, it was shown that exosomes derived from human adipose‐derived mesenchymal stem cells and iPSCs are respectively enriched in miR‐125a, miR‐181b, and miR‐1995‐5p, and promote angiogenesis, endothelial proliferation, and vascular repair through mechanisms potentially impaired by senescence.^[^ ^215^, ^216^ ^]^ On the other hand, exosomes from senile HUVECs exert detrimental effects on endothelial adhesion molecules, thereby facilitating a dysfunctional barrier behavior in younger ECs.^[^ ^217^, ^218^ ^]^ Importantly, induced senescent ECs are known for secreting EVs enriched with miR‐31, more present in elderly donors and able to inhibit osteogenic differentiation of mesenchymal stem cells (MSCs), thus supporting the hypothesis that these EVs in vivo could contribute to an aged systemic environment.^[^ ^219^ ^]^ Similarly, exosomal miR‐767 derived from senescent ECs was found to affect skin fibroblast behavior by promoting their senescence.^[^ ^220^ ^]^

Another example is described in the process of vascular calcification, where senescent ECs might be responsible for producing EVs carrying proteins (such as bone morphogenetic protein 2, BMP2; annexin A2; and annexin A6) which are normally involved in bone formation. These microvesicles are significantly present in plasma of old donors and were proven to promote human aortic smooth muscle cells calcification in vitro, thus suggesting their involvement in promoting the same process in vivo.^[^ ^221^ ^]^

### 3.11 Loss of Proteostasis and Disabled Macroautophagy

Disequilibrium between protein synthesis, maintenance, quality control, and degradation (i.e., loss of proteostasis) has long been known to be involved with ARDs such as Alzheimer's and Parkinson's disease.^[^ ^222^ ^]^ The disruption of protein homeostasis and subsequent accumulation of misfolded aggregates have also been observed in several CVDs.^[^ ^223^ ^]^ Multiple cellular components taking part in the proteostatic process are also affected in the aging endothelial compartment. First, chaperones that assist in protein folding, assembly, and disassembly, as well as in preventing misfolding, were found to have decreased expression or activity in aged or senescent ECs across multiple studies. Hwang et al. reported a diminished heat shock response in senescent ECs, followed by an increased protein aggregation.^[^ ^224^ ^]^ In addition, 70 kDa heat shock proteins (HSP70) were downregulated in aged vascular tissues from rats.^[^ ^225^ ^]^ Further, the activity of proteasome 20S, important for maintaining protein turnover, was diminished, and the ubiquitin levels increased in human atherosclerotic vascular tissues from old patients, suggesting an additional level of dysfunction in the endothelial protein homeostasis.^[^ ^36^ ^]^

Macroautophagy is a cellular process promoting the sequestration, digestion and recycling of proteins, cytosolic DNA, lipid vesicles, and also entire dysfunctional organelles. This task is performed through the formation of an autophagosome and its subsequent fusion with a lysosome for complete digestion of its content. A decrease in autophagy due to aging is a significant factor in decreased organelle renewal, which is a prominent characteristic of aging, along with the decline in proteostasis.^[^ ^226^ ^]^ La Rocca et al. showed that vascular markers of autophagy were reduced in old mice also having diminished endothelial‐dependent dilation (EDD), a condition frequently linked to aging. Additionally, it was shown that treatments with an autophagy enhancer were able to partially restore NO‐mediated EDD, suggesting a link between aging, autophagic dysfunction, endothelial dysfunction, and CVDs.^[^ ^227^ ^]^

Microvascular aging is exquisitely complex and exhibits a multitude of cellular and molecular hallmarks that deeply interconnect and influence one another. Although some of these hallmarks have been observed and described in other cell types and systems, a subset uniquely characterizes endothelial and vascular aging. Given the highly reactive and instructive role of the vascular system in maintaining tissue homeostasis, it is crucial to focus on those hallmarks that underlie these physiological functions rather than treating them all as equally important. In this context, phenotypes such as barrier integrity, vasoactive behavior, and tissue vascularization—and their underlying causal mechanisms—should be prioritized when studying how aging impairs endothelial biology and when seeking therapeutic strategies. Ultimately, targeting these key endothelial hallmarks offers the greatest promise for preserving vascular health and counteracting age‐related functional decline.

## 4 Other Cellular and Molecular Mechanisms of Vascular Aging

Vascular aging is characterized by structural and functional changes in blood vessels that occur with advancing age. It is well established that understanding the role of the cellular players involved in this process is crucial for elucidating the pathophysiology of age‐related vascular diseases and for the identification of potential therapeutic targets.

### 4.1 Pericytes

Pericytes are capillary‐associated mural cells located within the endothelial basement membrane. They interact with ECs by means of long processes, which allow the interconnection of a pericyte with several ECs simultaneously.^[^ ^228^ ^]^ Pericytes collaborate with ECs in the formation and maintenance of the endothelial barrier by means of tight‐, gap‐, and adherens junctions.^[^ ^229^ ^]^ A classic example is described in the brain, where they are crucial players within the neurovascular unit as they give a fundamental contribution to the functionality of the BBB.^[^ ^230^ ^]^

Pericytes are emerging as key regulators of vascular aging.^[^ ^231^ ^]^ With aging, the number of these cells decreases in various tissues, such as kidneys, heart, and brain,^[^ ^231^, ^232^ ^]^ resulting in increased vascular leakiness, and impaired capillary flow and structure.^[^ ^233^ ^]^ Additionally, aging may reduce the structural complexity of pericytes, as both their cell body size and the length of their cell processes are decreased in the brains of aged mice. This leads to a dramatic decrease in capillary pericyte coverage, which falls by about 50%.^[^ ^234^ ^]^ Similar findings were observed in the human brain, where the dimension of pericyte somata in frontal cortical capillaries in healthy old (>78 years) individuals was estimated to be 45% less compared to healthy younger (46–65 years) donors.^[^ ^232^, ^235^ ^]^

Liu et al. found that age‐related elevation of PDGF‐BB in serum and hippocampus promotes pericyte loss and BBB impairment through persistent stimulation that induces MMP14 and PDGFRβ shedding.^[^ ^236^ ^]^ Importantly, knockout of PDGF‐BB in pre‐osteoclasts reduced serum PDGF‐BB and limited age‐related hippocampal pericyte loss.^[^ ^236^ ^]^

Additionally, pericytes can undergo a phenotypic switch to fibroblasts (and myofibroblasts), which can contribute to ARD development.^[^ ^15^, ^237^ ^]^ This switch, which involves pathways such as transforming growth factor beta (TGF‐β),^[^ ^238^ ^]^ PDGF‐BB/PDGFRβ,^[^ ^239^ ^]^ and SIRT3,^[^ ^240^ ^]^ is responsible for the age‐dependent fibrotic tissue remodeling in several organs,^[^ ^237^ ^]^ including kidneys.^[^ ^237^ ^]^ Additionally, regulator of G‐protein signaling 5 (Rgs5) emerged as an important gene involved in pericyte‐mediated fibrosis. In fact, *Rgs5* is downregulated in pericytes within the heart of old mice. Deletion of this gene resulted in the expression of a profibrotic signature, which led to excessive ECM deposition and secretion of TGF‐β and PDGF‐BB.^[^ ^231^ ^]^

### 4.2 vSMCs

vSMCs are a type of smooth muscle cells specifically found in the walls of blood vessels, especially in arteries.^[^ ^241^ ^]^ They are characterized by the ability to acquire different phenotypes in a context‐specific manner, as exemplified by the transition from a contractile to a synthetic phenotype, which occurs as a consequence of vessel damage.^[^ ^242^ ^]^ In healthy individuals, the vast majority of vSMCs display a contractile phenotype distinguished by a state of quiescence and by the expression of contractile proteins, such as alpha smooth muscle actin (αSMA).^[^ ^243^ ^]^ When an injury affects the vessel, the switch to a highly synthetic phenotype is immediately triggered, resulting in a downregulation of contractile gene expression, upregulation of specific genes (e.g., osteopontin, epiregulin, and vimentin), increased proliferation, and augmented secretion of collagen, elastin, and MMPs. This way, the repair process is facilitated and, once the injury is fixed, vSMCs reacquire their contractile phenotype.^[^ ^242^ ^]^ However, evidence clearly demonstrates that the dysregulation of this finely controlled transition plays a pivotal role in atherosclerosis,^[^ ^39^ ^]^ vascular calcification,^[^ ^244^ ^]^ and pulmonary hypertension.^[^ ^245^ ^]^ Among the pathways involved in this switch, the TGF‐β pathway is known for its ability to stimulate vSMCs to adopt a synthetic phenotype through Smad3 signaling.^[^ ^246^ ^]^ Conversely, proteins like Klotho and the activation of peroxisome proliferator‐activated receptor‐γ (PPARγ) act as inhibitors of this phenotypic change, thus supporting the maintenance of a contractile state.^[^ ^247^, ^248^ ^]^ It is important to note that the senescent phenotype of vSMCs is similar to an irreversible synthetic phenotype as it is characterized by a decrease in terms of contractile markers and an increase in osteopontin expression.^[^ ^242^ ^]^ However, differently than their synthetic counterpart, senescent vSMCs show an increase of integrins, cytoskeleton proteins, and osteogenic genes (e.g., Runt‐related transcription factor 2, RUNX2; and BMP2).^[^ ^249^ ^]^ This kind of vSMCs is important in the context of vascular stiffness, ECM remodeling, and arterial aging. In particular, by releasing MMPs and stimulating elastin degradation, senescent vSMCs in the arterial intima promote inflammatory cell infiltration and vSMCs migration. Moreover, by secreting collagen, they affect the mechanical properties of the ECM. These changes are perceived by non‐senescent vSMCs through their integrin receptors and the resulting signaling culminates with the acquisition of a synthetic phenotype, hence contributing to the thickening of the arterial wall.^[^ ^242^ ^]^

### 4.3 Fibroblasts

Fibroblasts are responsible for the synthesis of essential molecules of the ECM (e.g., collagen type I, fibronectin, laminin, and proteoglycans)^[^ ^250^ ^]^ and for the secretion of endothelial basement membrane components (especially laminin and collagen type IV).^[^ ^251^ ^]^ Moreover, they are directly involved in the process of new blood vessel formation as they express pro‐angiogenic factors (such as fibroblast growth factor, FGF; MCP‐1; and VEGF)^[^ ^252^, ^253^ ^]^ and secrete fibronectin, which facilitates the elongation of new developing microvessels.^[^ ^254^ ^]^ They also secrete SDF‐1 which is important for EPC recruitment.^[^ ^255^, ^256^ ^]^

Aging affects fibroblasts without exception, leading to modifications at the transcriptomic, metabolic, and phenotypic level.^[^ ^129^ ^]^ In skin fibroblasts from old donors, for example, the basal production of IL‐6 was found to be slightly higher than in fibroblasts from young donors, suggesting that this cell type may also contribute to inflammaging.^[^ ^258^ ^]^ Another phenomenon that occurs in aging fibroblasts is senescence. In the aging skin, for instance, while the total number of fibroblasts is reduced, the population of senescent fibroblasts is increased.^[^ ^259^ ^]^ This accumulation is linked to an increased release of SASP‐associated factors that lead to imbalanced ECM homeostasis, chronic inflammation, and loss of cell identity.^[^ ^260^ ^]^

“Mid‐old” fibroblasts, recently identified as cells occupying an intermediate state between proliferating and senescent phenotypes, represent major contributors to aging‐related organ decline alongside senescent fibroblasts, due to their reduced ECM formation, proliferation, and regenerative capacity compared to young cells.^[^ ^261^ ^]^ Studies of skin fibroblasts from elderly donors further revealed a distinct aging‐associated secretome, different from the SASP profile, which may influence skin aging processes.^[^ ^262^ ^]^

The importance of fibroblasts in vascular aging is highlighted by their contribution to the onset of various age‐related CVDs.^[^ ^38^ ^]^ As mentioned, they are involved in all stages of atherosclerosis: in the initial phase, adventitial fibroblasts are among the first cells to respond to injury and become activated, thus participating in neointima and initial fibrous plaque formation.^[^ ^38^ ^]^ In advanced stages, they regulate the inflammatory response and maintain the structural integrity of the atherosclerotic plaque.^[^ ^38^ ^]^ Similarly, during cardiac fibrosis, their pathological activation results in the excessive deposition of ECM, thus leading to detrimental consequences in cardiac health.^[^ ^263^, ^264^ ^]^ Fibroblast aging can negatively affect microcirculation, as shown by 3D human skin microvasculature models: fibroblasts from individuals of various ages differentially modulate EC function. Notably, young fibroblasts were able to reverse multiple features of vascular degeneration observed in aged microenvironments—such as basal membrane damage, inflammation, and impaired vessel permeability—when incorporated into vascular networks with ECs from an old donor.^[^ ^265^ ^]^

### 4.4 Immune Cells and Immunosenescence

Immunosenescence is an age‐related condition characterized by progressive deterioration of the immune system with impaired adaptive and innate immune responses, contributing to ARD onset, poor vaccination outcomes, and increased susceptibility to infections and malignancies. Although the majority of previously described aging hallmarks (e.g., senescence, mitochondrial dysfunction) also affect immune cells, immunosenescence includes additional specific manifestations such as hematopoietic stem cell dysfunction, disrupted naïve/memory ratios in T and B cells, impaired new antigen responses, shifts in T lymphocyte subpopulations with increased CD4/CD8 ratios, increased secretion of inflammatory cytokines, and overall immunodeficiency. For a more complete description of this vast array of hallmarks, we invite the reader to consider some recent comprehensive reviews.^[^ ^266^, ^267^ ^]^ The intricate link between immune cells and vascular aging involves both the innate and the adaptive branch of the immune system.^[^ ^28^, ^268^, ^269^, ^270^ ^]^ The best example of this connection is represented by the vast array of immune cells involved in atherosclerosis as previously discussed in the Macrovascular Aging in CVDs box. Other than atherosclerosis, the immune system takes part in other phenomena linked with vascular aging. Macrophages, for example, are involved in the process of vascular calcification. Indeed, macrophages tend to shift from an anti‐inflammatory (M2) to a pro‐inflammatory (M1) phenotype with aging.^[^ ^271^ ^]^ By releasing TNF‐α, M1 macrophages promote the upregulation of carbonic anhydrase I (CA1) and carbonic anhydrase II (CA2) in vSMCs, thus contributing to the mineralization process.^[^ ^272^ ^]^ In turn, calcified vSMCs induce the differentiation of macrophages into osteoblasts through the RANKL/RANK/OPG system, thus activating a positive feedback loop that promotes vascular calcification.^[^ ^271^ ^]^ Additionally, aging is correlated with elevated serum levels of complement component C3 and a higher expression of C3aR1 in ECs ^[^ ^273^ ^]^. This increase might result in an over‐activation of the C3a‐C3aR1 signaling in the endothelium, thus promoting vascular inflammation, increased BBB leakiness, lymphocyte infiltration,^[^ ^274^ ^]^ and neurodegeneration.^[^ ^275^ ^]^ Furthermore, in addition to their role in atherosclerosis progression, AT1 Agonistic Autoantibodies were revealed to contribute to the progression of vascular aging by stimulating the induction of senescence in ECs through the activation of AT1 receptors.^[^ ^276^ ^]^

Lastly, as reported for CHIP, distinct mutated clonal immune cell populations profoundly influence vascular aging, primarily by promoting an elevated pro‐inflammatory phenotype that may contribute to the inflammaging process. Although a substantial body of evidence has established CHIP as a causative factor in macrovascular conditions, direct evidence for its involvement in microvascular‐driven diseases is still lacking, highlighting the need for further research in this area.^[^ ^277^ ^]^ Nevertheless, existing reports indicate that ECs are significantly affected by the pro‐inflammatory profile of upregulated cytokines in CHIP, suggesting this may be at least one key hallmark impacting microvascular health and associated tissues.

In conclusion, the evidence reported in this chapter highlights how multiple cell types belonging to or interacting with the vascular niche actively contribute to vascular aging. Beyond the cell‐autonomous changes described, increasing data point to the importance of intercellular communication and to the recognition that vascular aging follows organ‐specific trajectories, consistent with the well‐documented heterogeneity of endothelial and mural cells across tissues.^[^ ^7^, ^15^, ^71^, ^72^, ^278^ ^]^ Embracing this level of complexity and integrating these dimensions—cellular cross‐talk, aging mechanisms, and phenotypic plasticity within organ‐specific vascular contexts—represents a promising avenue to refine our mechanistic understanding of vascular aging and to guide the design of targeted, tissue‐adapted therapeutic strategies aimed at preserving vascular health throughout life.

## 5 Modeling Vascular Aging

Aging is an exceedingly complex process, and its study requires the use of a variety of model systems in order to answer the wide range of questions that arise in the field. In this context, the selection of an appropriate model is of paramount importance and should be strictly related to the specific research question being addressed. Over the past decades, aging models have largely been categorized within a dichotomous framework, distinguishing between reductionist in vitro models and non‐reductionist in vivo models. While the formers offer a simplified view of biological phenomena, they often fall short in addressing the complexity of an entire organism. In contrast, the latter provide a more holistic perspective but present significant challenges due to confounding factors, making these studies time‐consuming, costly, and at times difficult to interpret.^[^ ^279^ ^]^ In this section, we will critically present the most commonly used model systems employed in vascular aging research, discuss their strengths and weaknesses, and elaborate on ideal requirements and parameters that should be considered to increase the relevance of the next‐generation of in vitro models to promote a smoother integration with results achieved in vivo.

### 5.1 Animal Models to Study Aging Biology and Vascular Aging

**Figure 3:**
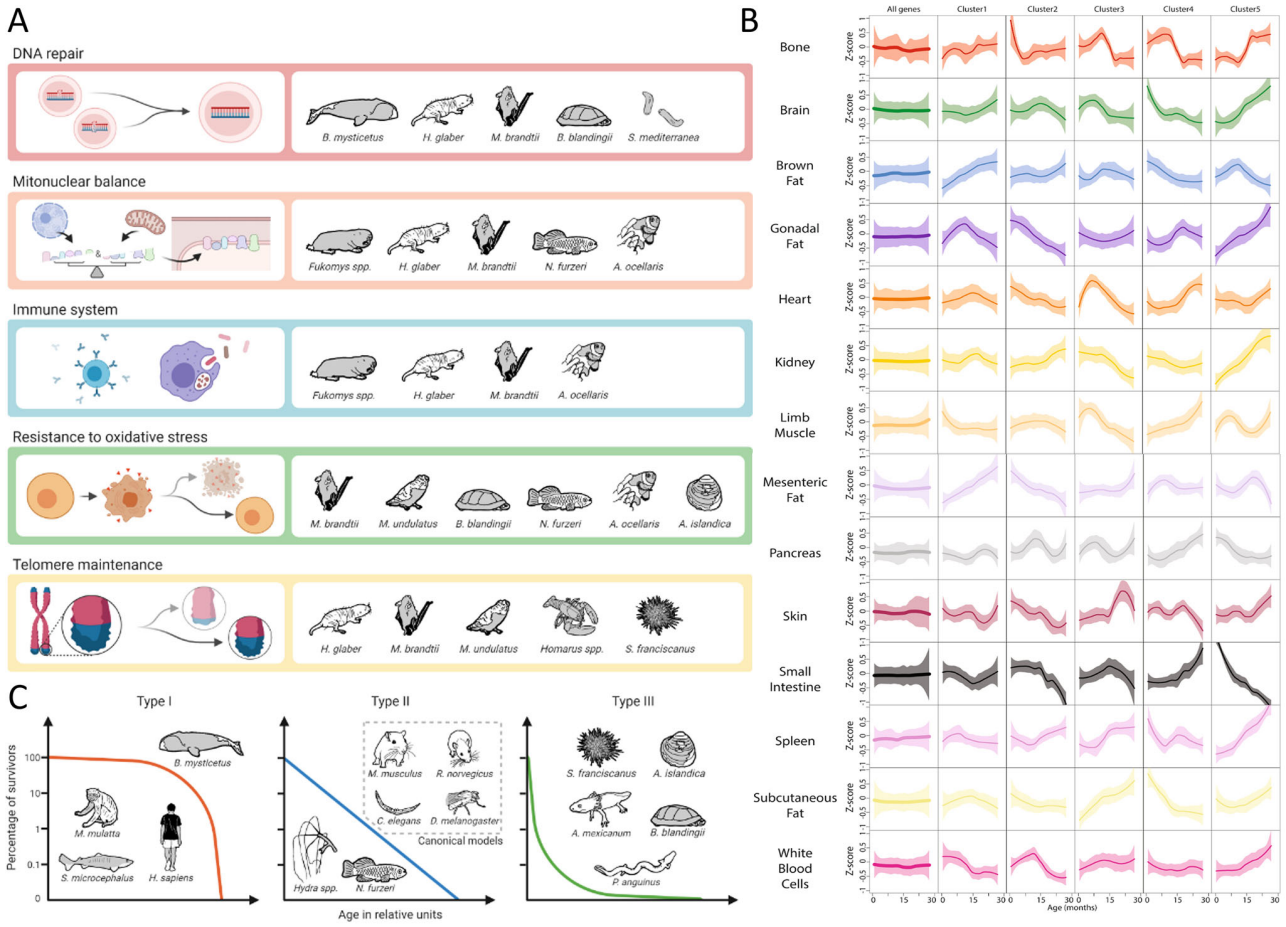
Capturing features of aging through in vivo model systems. A) Alternative in vivo models of aging are pivotal in understanding how biological pathways and molecular mechanisms of aging impact lifespan in living organisms. C) Survival curve types highlight how different organisms show peculiar aging patterns, suggesting the existence of species‐specific aging mechanisms. Reproduced (Adapted) under the terms of the CC BY 4.0 license.^[^ ^280^ ^]^ Copyright 2021 Holtze, Gorshkova, Braude, Cellerino, Dammann, Hildebrandt, Hoeflich, Hoffmann, Koch, Terzibasi Tozzini, Skulachev, Skulachev and Sahm. B) Aging‐driven expression trajectories of organ‐specific genes. Using mouse models, the authors were able to observe gene clusters with expression trajectories that behave differently depending on the specific organ. Reproduced (Adapted) with permission. ^[^ ^278^ ^]^ Copyright 2020, The Author(s), under exclusive license to Springer Nature Limited.

Traditionally, aging research aimed at understanding the basic aging pathways shared with humans has been performed in animal models, such as *Caenorhabditis elegans*, *Drosophila melanogaster*, *Mus musculus* or the shortest‐lived vertebrate *Nothobranchius furzeri* (**Figure** **3**).^[^ ^280^ ^]^ Using these models, the importance of molecular players (e.g., sirtuins) and the life‐extending properties of some molecules (e.g., resveratrol) and interventions (e.g., caloric restriction) was established.^[^ ^281^, ^282^ ^]^ In fact, these organisms present a convenient life span that makes them suitable for aging studies while having some basic biological and genetic characteristics in common with humans.^[^ ^283^ ^]^

Importantly, animal models present some interesting advantages. First, they allow for the study of aging in the context of a whole organism, thus enabling the extraction of information at the level of tissues and organs, and capturing the complete array of possible interactions between them. This concept could be showcased in the line of work started by Kenyon et al., who demonstrated that the insulin/insulin‐like growth factor 1 (IGF‐1) signaling pathway regulates longevity in different model organisms.^[^ ^284^ ^]^ It was only by studying this phenomenon in mouse that the role of the growth hormone (GH)/IGF‐1 axis in mammalian aging was confirmed. This axis is the result of integration of information between different tissues, being IGF‐1 a pleiotropic factor. However, even though several long‐lived mouse strains with reduced activity of the GH/IGF‐1 axis were observed, human data still seem to be contradictory.^[^ ^285^ ^]^

Another example was displayed by Schaum et al., who performed bulk RNA sequencing of 17 organs and plasma proteomics across the lifespan of mice. This study identified converging and deviating aging transcriptomic signatures across tissues, their shift during aging, as well as the influence of sex on these signatures. This powerful approach confirmed that common hallmarks of aging (e.g., inflammaging and mitochondrial dysfunction) do exist and have distinct temporal dynamic patterns. Furthermore, it showed that organ‐specific patterns are also present and that the plasma composition is correlated with some of the transcriptomic changes detected in different organs.^[^ ^278^ ^]^

Regarding the vascular compartment, single‐cell RNA sequencing (scRNAseq) analyses using aged mice showed that ECs from vastly different and distant tissues share overlapping transcriptional programs and tend to cluster together, suggesting the existence of a more broad and common endothelial aging program.^[^ ^278^, ^286^ ^]^ Finally, animal models allow for the study of the effect of a complex set of alterations that some interventions have on the aging process at the level of different tissues, such as exercise and caloric restriction.^[^ ^283^ ^]^ These two candidate life‐extending interventions were initially tested on mice and subsequently moved to human trials due to the deep biological impact that they exert on the organism.

#### 5.1.1 Heterochronic Parabiosis – A key In Vivo Model for Aging Research

Heterochronic parabiosis is a condition resulting from the surgical connection of the circulatory systems of two animals of different chronological ages. It was first described by Paul Bert in 1864 and has been extensively used to study the effect of a young systemic environment on an old organism and vice versa.^[^ ^287^ ^]^ Conboy et al. were the first to demonstrate through heterochronic parabiosis that the exposure of aged mice to factors from a young animal was able to activate hepatic and muscle stem cells, thereby counteracting major aging hallmarks (i.e., stem cell exhaustion and reduced regenerative capacity) and finally rejuvenating the aged tissues.^[^ ^288^ ^]^ Moreover, Villeda et al. showed that blood‐borne factors are sufficient to negatively regulate adult neurogenesis in brains of young heterochronic parabionts and decrease synaptic plasticity.^[^ ^289^ ^]^ Coupling this experimental set‐up with metabolomic and proteomic analyses has enabled the identification of factors and molecular pathways mediating multiple aging processes across the organism, together with potential therapeutic targets able to slow or reverse specific aspects of aging. Examples are growth differentiation factor 11 (GDF11), oxytocin, apelin, and β2‐microglobulin.^[^ ^290^ ^]^

Circulating factors identified through heterochronic parabiosis are important in the context of vascular aging and have the potential to accelerate or revert some aging processes. In this context, a great example was reported by Katsimpardi et al., who demonstrated that GDF11 from young blood improved the vasculature of the aging mouse brain.^[^ ^291^ ^]^ Using this model, Pàlovics et al. performed scRNAseq analysis on endothelial subpopulations in different tissues and organs. Collected data showed that ECs are transcriptionally susceptible to an aged/young systemic environment, but their response displays only a discrete tissue‐specificity, suggesting that this effect relies more on the direct contact between vessels and blood‐borne factors.^[^ ^286^ ^]^ In a similar experimental set‐up, Ximerakis et al. focused on ECs from parabiotic mice brains. By performing scRNAseq, they detected transcriptionally different EC populations within this organ, with expression patterns heavily influenced by parabiosis, emphasizing their vulnerability to the effects of aging and the parabiosis‐mediated rejuvenation or accelerated aging.^[^ ^292^ ^]^

Non‐cell‐autonomous pro‐ and anti‐geronic factors affecting the cerebral microvasculature were also investigated by Gulej et al., who performed heterochronic parabiosis experiments to study the impact of systemic factors on brain vascular rarefaction and blood–brain barrier (BBB) leakiness. Using this setup, coupled with intravital two‐photon microscopy, the group observed a significant decrease in brain microvascular length density and network complexity in old isochronic parabionts compared with young isochronic parabionts. These phenotypes improved in old heterochronic parabionts and were worse in young heterochronic parabionts relative to their young isochronic controls. Moreover, fluorescent dextran perfusions revealed an age‐related increase in BBB leakiness that was partially reversed by young systemic factors and worsened by an old systemic environment.^[^ ^293^ ^]^ Another interesting study based on parabiosis from the same research group, highlighted that NVC and cerebral blood flow are not only impaired with age, but are also partially restored by young systemic circulating factors. This suggests that age‐related vascular cognitive impairment could, at least in part, be counteracted by bloodborne anti‐geronic factors.^[^ ^294^ ^]^

Overall, heterochronic parabiosis remains an unparalleled in vivo model to probe the systemic regulation of vascular aging, having revealed critical circulating factors and molecular pathways. Yet, it represents a highly non‐physiological condition, imposes significant stress on the animals, and poses challenges for direct translational relevance. Moreover, in the context of aging research, most parabiosis studies have primarily focused on soluble blood components, while the contribution of circulating cells has remained comparatively underexplored. Acknowledging these limitations, the development of in vitro models that can faithfully recapitulate key aspects of this experimental setup would represent a powerful avenue to further dissect systemic regulation of vascular aging.

#### 5.1.2 Genetic Models of Aging

Despite the convenience of studying systemic and organ‐related aging using mice, genetic models of accelerated aging are generally preferred due to the faster onset of ARDs and hallmarks. In these experimental setups, molecular mechanisms underlying the biological aging of the animal are disrupted through genetic engineering, resulting in so‐called progeroid or premature aging phenotypes. For example, given the importance of DNA and nuclear integrity to cellular and organismal aging, multiple pathways can be targeted to mimic progeroid syndromes that are also rarely observed in humans. These include mice bearing the LmnaG609G mutation, which causes nuclear lamina defects and develops the “murine analog” of the classical human Hutchinson‐Gilford Progeria Syndrome (HGPS), and hypomorphs for the mitotic spindle checkpoint protein BubR1, which display chromosomal instability and premature aging. Extensive descriptions of these and other genetic models obtained through genetic manipulations of several aging hallmarks, along with their related phenotypes, can be found in another review.^[^ ^295^ ^]^ The seminal work of Weeda et al. generated mutant mouse strains carrying null and hypomorphic alleles of ERCC1, an endonuclease complex subunit pivotal for both nucleotide excision repair and homologous recombination repair.^[^ ^296^ ^]^ While the initial investigation of this model revealed a greatly reduced lifespan (≈6 months) accompanied by multiple age‐related phenotypes, such as premature senescence and other cellular defects across several organs, subsequent studies confirmed that these mice also exhibited increased signs of CVDs, including hypertension, vascular stiffening, and loss of vasomotor control, compared to age‐matched wild‐type mice. This underscores, once again, the contribution of accelerated aging to the onset of CVDs. Notably, the involvement of different aging hallmarks and cell types in the development of cardiovascular conditions has been reported: dysfunctional ECs, impaired ECM remodeling and deposition, as well as detrimental phenotypic switching of vSMCs have all been described in these mice.^[^ ^107^, ^297^ ^]^

Although these models represent a convenient alternative to normal chronological aging animals, elucidating precise cell‐specific aging mechanisms to disentangle their contribution to systemic aging is a current priority. Traditionally, research on macrovascular aging has mainly aimed at preventing major cardiovascular events. However, the current trend shifts attention to how dysfunction in the endothelium and microvasculature contributes to, and shapes, the aging process in specific tissues. In this context, models carrying endothelial specific removal of ERCC1 showed not only endothelial dysfunction characterized by abolished NO‐mediated endothelium‐dependent vasodilation, increased senescence and superoxide production, but also reduced skin microvascular dilation, increased renal microvascular leakiness, decreased pulmonary perfusion, and BBB disruption in the brain.^[^ ^298^, ^299^ ^]^ Gao et al. generated and analyzed an EC‐specific *Tert* knockout model to investigate how accelerated telomere attrition—a well‐established hallmark of aging—in ECs impacts tissue pathophysiology. Among the observed effects, this genetic alteration promoted an endothelial glycolytic shift, impaired capillary network formation ex vivo, increased tissue hypoxia, and induced secondary senescence‐like changes in non‐EC populations. Notably, while loss of *Tert* accelerated telomere shortening and senescence in ECs residing in adipose tissue, the same genetic background led to increased brain vascular leakiness, impaired cognitive function, and reduced muscle fatigue resistance. These latter effects are likely mediated through telomere‐independent mechanisms, suggesting the existence of aging mechanisms that still have to be completely understood.^[^ ^113^ ^]^ Another recent publication reported the use of an HGPS‐like model in which ECs selectively express progerin. These mice exhibited enhanced endothelial DNA damage and senescence, along with a pro‐inflammatory state characterized by increased VCAM1 and ICAM1 mRNA expression. Using this model, Rolase et al. demonstrated that endothelial aging, including senescence, drive excessive inflammation, as evidenced by elevated neutrophil adhesion and crawling along the vessel wall, as well as neutrophil‐dependent microvascular leakage.^[^ ^300^ ^]^ This cell type–restricted genetic modeling therefore, represents a highly promising approach to dissect endothelial specific aging mechanisms and identify potential effects of microvascular dysfunction in driving tissue‐specific aging phenotypes. At the same time, it is important to recognize a critical limitation: accelerated aging models cannot fully recapitulate the complexity and temporal dynamics of natural aging. As a result, these experimental settings may bias outcomes toward features of artificially induced aging, in which the relative contribution of individual aging hallmarks, and their integration within the broader physiological aging process, may not be faithfully represented.

#### 5.1.3 Weaknesses of Animal Models

Although the study of human progeroid syndromes and the analysis of centenarian genomes have provided valuable information regarding the genetic determinants of human aging and longevity, the use of humans in studies related to aging poses certain obstacles due to factors such as the extensive lifespan.^[^ ^283^ ^]^ Therefore, animal models are the most promising substitute to approximate human aging and geroprotection mechanisms. However, it must be noted that there are some key limitations. In fact, several crucial aspects of aging biology are known to be significantly different between animal models and humans. *M. musculus* is probably the most relevant and used model for the study of aging, due to its close resemblance to humans in terms of genetic background and physiological structure, the possibility to easily manipulate its genes, its short lifespan, and simple breeding process.^[^ ^301^ ^]^ However, it is important to remember that the genetic background and the physiological structure of mice are not identical to humans, and aging is an extremely multifaceted process that arises from the interactions between components at the level of molecules, cells, tissues, and organs. Not surprisingly, several publications reported differences in how the aging biology is regulated in humans and mice, and the existence of species‐specific aging pathways. In 2007, Zahn et al. created the AGEMAP, a mouse aging gene expression database across different tissues. Thanks to this tool, the authors were able to compare transcriptional profiles for aging in mice and humans and found no overall correlation between the age‐driven gene expression changes in the two species, hence suggesting a fundamental difference in the aging processes.^[^ ^302^ ^]^ Years later, Yang et al. compared the aging genes from the AGEMAP database with human aging transcriptional patterns derived from GTEx (Genotype‐Tissue Expression) consortium data and, consistently with previous studies, suggested a very large expression difference in aging genes between the human and mouse organisms. However, the authors also highlighted that this lack of correlation could also be due to differences in terms of sample size, gender distribution, and age range between the two datasets.^[^ ^303^ ^]^ In 2019, using GTEx and Gene Expression Omnibus (GEO) data coupled with more sensitive algorithms than the previous studies, Zhuang et al. found that 9 out of 18 tissue aging signatures were correlated between humans and mice, showing that there is a balance between transcriptional similarity and species‐specificity between the two species.^[^ ^103^ ^]^ This aspect was further confirmed when more accurate analyses were performed at the level of single tissues.^[^ ^304^ ^]^

From all these studies, the divergence concerning aging biology across different animal models and humans arises. Such discrepancies also limit the translation of knowledge gathered through animal experiments in the context of human aging. Important examples are the clinical trials aimed at testing the effect of different interventions on human ARDs, health, and biological age/lifespan extension, which have been partly disappointing despite their efficacy in animal models.^[^ ^305^ ^]^

### 5.2 In Vitro Models to Study Aging Biology: Strengths and Weaknesses Through Representative Systems

**Figure 4:**
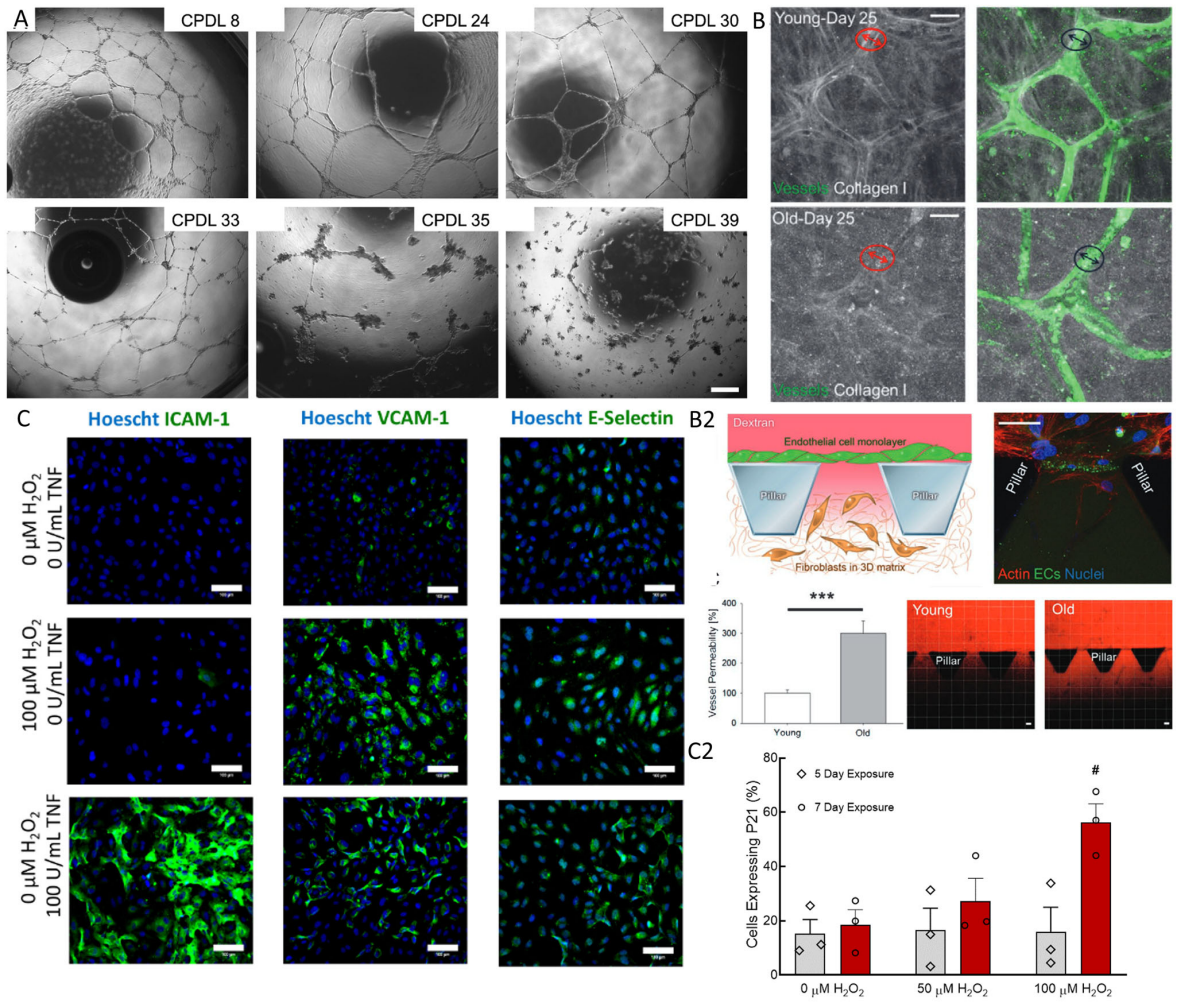
Capturing features of aging through in vitro model systems. Human primary ECs strains to be used in in vitro models of aging recapitulate aspects of organismal aging biology. A) Human microvascular ECs lose the ability to organize in tubes as they age while increasing cumulative population doubling levels (CPDL) in vitro. Reproduced (Adapted) with permission. ^[^ ^307^ ^]^ Copyright 2010, Elsevier Inc. B) 3D in vitro models of microvascular networks recapitulate aging features (i.e., changes in basement membrane deposition) when put in contact with young/aged microenvironments (i.e., young/aged fibroblasts). (B2) The impact of aging on endothelial biology (i.e., endothelial permeability) can be studied with simple in vitro models employing EC monolayers and fluorescent dextran diffusion gradients. Reproduced (Adapted) under the terms of the CC BY‐NC‐ND 4.0 license. ^[^ ^265^ ^]^ Copyright 2020, Copyright by Simone Bersini, Rafael Arrojo e Drigo, Ling Huang, Maxim N. Shokhirev, Martin W. Hetzer. C) Primary ECs show aging features such as expression of adhesion molecules (C) or p21 upregulation (C2) when oxidative stress is induced artificially in culture by treating with H_2_O_2_. Reproduced (Adapted) under the terms of the CC BY 4.0 license. ^[^ ^311^ ^]^ Copyright 2020, Copyright by Ellen E. Salmon, Jason J. Breithaupt and George A. Truskey.

In vitro models are a valuable substitute for animal models due to several reasons. First, they are easier to manipulate and treat, and can be created using human cells, thus presenting a more direct relevance to human aging biology. Second, they allow for the exclusion of several confounding factors decoupling the influence of other cell types and systemic factors. Nonetheless, different levels of complexity can be reproduced in vitro, harnessing technologies such as 3D co‐cultures with supportive biomaterials mimicking different ECMs, organoids and microphysiological systems. Third, they have a high level of reproducibility, present easier scalability and enable the extraction of a wide range of information in a relatively short time when coupled with high‐throughput techniques and assays, therefore representing a powerful tool for the study of human aging biology and the screening of potential geroprotective interventions.^[^ ^306^ ^]^ **Table** **1** and **Figure** **4** summarize representative in vitro models that have been developed and employed to investigate endothelial and vascular aging mechanisms, including their characteristic aging phenotypes, methodological advantages, and key findings that contribute to our understanding of vascular aging pathways.

**Table 1:** Examples of key endothelial aging models, including their aging‐related phenotypes, research advantages, major findings, and corresponding references, illustrating mechanisms underlying endothelial and vascular aging across various experimental approaches.

Model	Aging phenotypes	Advantages	Findings
Serial passaging of ECs in vitro (2D culture) ^[^ ^307^ ^]^	Decreased proliferation, increased expression of senescence markers, impaired angiogenic potential and tube formation.	Allows for the study of aging impact on basic endothelial aspects (e.g., cell morphology, β‐galactosidase activity, replication).	In vitro aging recapitulates in vivo phenotypes of endothelial aging. Beta gal staining is not suitable for assessing senescence in tube‐forming ECs. Suitable for high throughput assays.
Arterial ECs model in early (young) and late (old) stages of replication ^[^ ^312^ ^]^	Morphological changes and increased expression of genes with the aging phenotype, including β‐galacosidase, p65 subunit of NFκB and TGF‐β.	Complex cell behaviors (e.g., EndoMT) are reproducible in vitro and can be studied in aging contexts.	Role of inflammation in inducing EndoMT in the context of endothelial aging.
Simple BBB model using trans wells to evaluate the effect of senescence on barrier breakdown ^[^ ^310^ ^]^	Induced endothelial senescence in vitro, decreased proliferation and increased p16 and p21 expression.	Uses combined cell types to recapitulate complex phenotypes and dissect the effects of single cell type in age‐related dysfunction of the BBB.	Impaired barrier integrity in the BBB model, altered tight junction structures with increased senescence.
Tissue‐engineered blood vessels recapitulating arterial phenotypes to study the relative influence of endothelial cell and vSMCs senescence on vasoreactivity ^[^ ^311^ ^]^	Induced senescence with increased p21 expression and reduced NOS3. Increased inflammation was also detected.	Study individual contribution of aged/senescent cell types to vascular reactivity and potentially microenvironment (e.g., ECM deposition).	Endothelium‐Dependent Vasoreactivity is diminished in senescent settings. Vascular inflammation is present with senescent ECs.
Arteriole‐scale tissue engineered blood vessel using iPSC‐derived SMCs from an HGPS patient ^[^ ^313^ ^]^	Reduced vasoactivity, increased vascular wall thickness, calcification and apoptosis.	Isolate the effect of the HGPS iSMCs on vascular function.	HGPS iSMCs impact in vitro vasculature by conferring aged phenotypes.
Long‐living human microvascular networks to study how the cellular microenvironment and serum from healthy donors of different ages modulate microvascular functionality ^[^ ^265^ ^]^	Impaired vascular basal membrane deposition, altered microvascular density.	Study the individual contribution of cell types to overall aged vascular phenotypes, presence of 3D EMC, and physiological vascular structures.	Fibroblasts from young donors restore the functionality of aged ECs, an effect also achieved by serum from young donors.

Early studies demonstrated that ECs passaged in vitro recapitulate aging phenotypes observed in vivo, including decreased proliferation, senescence marker expression, and impaired angiogenic capacity. However, traditional 2D models present significant limitations, being oversimplified versions of biological phenomena that lack tissue‐scale complexity and mechanical cues.^[^ ^307^, ^308^, ^309^ ^]^

Advanced models have addressed these limitations through increasingly sophisticated approaches. Co‐culture systems introduced cellular heterogeneity, with BBB models demonstrating how EC senescence contributes to barrier dysfunction.^[^ ^310^ ^]^ Organotypic microphysiological models incorporate human cell types within engineered ECMs, enabling precise control over mechanical and biochemical stimuli to better recapitulate native tissue complexity. Three‐dimensional organoids and microfluidic devices have further advanced the field, with tissue‐engineered blood vessels successfully modeling endothelial aging hallmarks, including senescence, reduced eNOS expression, and impaired vasodilation.^[^ ^311^ ^]^

Despite these advances, current models struggle to integrate sufficient biological complexity with the scalability required for high‐throughput screening of geroprotective compounds. The field lacks comprehensive models that combine 3D multicellular populations, hemodynamic stimuli, and organ‐specific interactions essential for vascular aging research.

## 6 Future Directions in Vascular Aging Research

Future vascular aging research is expected to prioritize several concrete and impactful directions. First, the field is moving toward a more holistic assessment of aging by employing marker panels that capture the multifaceted aspects of the aging spectrum. Too often, single hallmarks of aging have been used to define the aging state of a cell, with terms such as “senescent” and “aged” applied interchangeably, as if senescence alone could fully recapitulate the molecular aging state. Second, the integration of multi‐omics approaches with AI is anticipated to advance beyond simple correlation, enabling the identification of causal pathways that drive vascular decline. Third, substantial progress will come from the development and validation of vascular‐specific aging clocks—leveraging cell‐free DNA (cfDNA) methylation, vascular‐derived miRNAs, and proteomic markers—to provide accurate and minimally invasive measures of vascular biological age. Fourth, longitudinal cohort studies and interventional trials will be crucial to standardize biomarker panels and to assess whether modifying biological aging markers translates to improved vascular outcomes. Finally, as highlighted in the modeling section, there is a need for advanced models to mimic complex human aging mechanisms, thereby improving the translation of anti‐aging interventions into clinical practice. Together, these directions will not only deepen mechanistic understanding but also accelerate the path toward precision vascular medicine and longevity therapies.

### 6.1 Targeting Vascular Aging for Intervention

As discussed, vascular aging involves both structural and functional changes in blood vessels and increases susceptibility to ARDs. For this reason, targeting vascular aging holds promise for mitigating the burden of associated pathologies. Recent research has identified several approaches for delaying vascular aging, focusing on molecular mechanisms, lifestyle modifications, and pharmacological approaches. **Table** **2** provides a comprehensive overview of anti‐aging interventions aimed at mitigating vascular and endothelial aging, detailing each strategy's direct and indirect mechanisms of action, the resulting vascular phenotypes, and the strength of the supporting evidence.

**Table 2:** Overview of some anti‐aging interventions targeting vascular aging, detailing their mechanisms of action, observed cardiovascular outcomes, levels of supporting evidence, and relevant references, encompassing pharmacological, biotechnological, dietary, lifestyle, and genetic approaches.

Intervention	Mechanisms of action	Cardiovascular outcome
Rapamycin ^[^ ^314^, ^315^ ^]^	Inhibition of the mechanistic target of rapamycin (mTOR) pathway (i.e., nutrient sensing).	Improved cardiovascular function, attenuated heart pathologies.
Metformin ^[^ ^316^, ^317^, ^318^ ^]^	AMPK activation (i.e., metabolic activity regulation), suppresses SASP, reduces inflammation and oxidative stress.	Improved endothelial function, prevention of endothelial inflammatory responses in the arterial wall, and reduces early progression of coronary plaques.
Irisin ^[^ ^319^, ^320^ ^]^	Pleiotropic effects on endothelial signaling pathways: AMPK and ERK, PI3K/Akt axis stimulation, SIRT6 activation, Nrf2 and antioxidant response, NO bioactivity increase and others.	Reduces vascular inflammation, slows atherosclerotic plaque development/ progression, alleviates ischemia/reperfusion injury, and inhibits vascular calcification.
Senolysis: Dasatinib + quercetin (D + Q) ^[^ ^321^, ^322^ ^]^	Selective elimination of senescent ECs within the vascular wall.	Reduces vascular senescence, vasomotor dysfunction and arterial stiffness.
Navitoclax (ABT‐263) ^[^ ^323^, ^324^ ^]^	Selective elimination of senescent ECs within the vascular wall	Reduces vascular senescence, improves arterial function, ameliorates NO bioavailability, and reduces oxidative stress and vascular stiffness.
PAI‐1 antagonists ^[^ ^325^, ^326^ ^]^	Inhibition of PAI‐1, telomere preservation, suppression of p16 expression, prevents PAI‐1 binding to eNOS, restores NO production.	Protective against the development of hypertension, cardiac hypertrophy, and periaortic fibrosis, enhances endothelium‐dependent vasodilation.
Endothelin receptor antagonists (ERAs), e.g., Bosentan, Ambrisentan, Macitentan ^[^ ^327^, ^328^, ^329^ ^]^	Antagonize endothelin mediated vasoconstriction, promoting vasodilation and improving blood flow, counteracts senescence induction, inflammation and SASP modulation.	Improves arterial stiffness, decreases blood pressure and counteracts hypertension, ameliorates cerebral vasospasm.
Statins ^[^ ^330^, ^331^, ^332^, ^333^, ^334^ ^]^	Pleiotropic effects, including chronic inflammation reduction, upregulation of eNOS expression, oxidative stress decrease, and EPC mobilization, reduces AGEs.	Reduces vascular stiffness, myocardial infarction, stroke and general CVDs incidence.
Platelet rich plasma (PRP) ^[^ ^335^, ^336^, ^337^, ^338^ ^]^	Enhance ECs proliferation and migration, reduce inflammation, modulates SASP, promotes angiogenesis.	Vascular function improvement, microcirculation remodeling, enhances blood perfusion in ischemic myocardium, prevents vascular leakage and fibrosis, induces tissue regeneration.
Long‐term, regular physical exercise (in particular endurance training) ^[^ ^339^, ^340^, ^341^, ^342^, ^343^, ^344^, ^345^, ^346^, ^347^ ^]^	Improves Endothelial function, reduces vascular inflammation, decreases oxidative stress, increases the reparative capacity of EPCs, improves flow‐mediated vascular dilation.	Reduced arterial stiffness, improved cardiovascular health metrics (Fuster‐BEWAT score), extends cognitive function.
Specific diets (e.g., Mediterranian style diet) ^[^ ^348^, ^349^, ^350^ ^]^	Diminishes ROS production and endothelial senescence, protects against accelerated telomere shortening, suppress NF‐κB signaling, reducing inflammation and modulating the SASP, increases circulating EPCs.	Improves systolic blood pressure and arterial stiffness, increases flow‐mediated dilation and promotes general reduced cardiovascular risk.^[^ ^357^ ^]^
Caloric restriction regimens ^[^ ^24^, ^351^ ^]^	mTOR inhibition, SIRT1 activation, AMPK phosphorylation, increased NO bioavailability and reduced arterial collagen accumulation and elastin remodeling.	Attenuates arterial stiffening, restores EDD, and reduces CVD risk factor.
Dietary L‐arginine, nitrate, and nitrite supplementation ^[^ ^352^, ^353^ ^]^	Enhances NO bioavailability.	Maintains vascular function, improves blood pressure, arterial stiffness, and cardiovascular health.
Antioxidant supplementation (α‐tocopherol, γ‐tocopherol, coenzyme Q10, vitamin C, carotenoids) ^[^ ^354^, ^355^, ^356^ ^]^	Enhanced endothelial function, reduced oxidative stress and inflammatory damage, and preserves DNA integrity.	Delayed CVD progression, reduced vascular stiffness, improved exercise induced vasodilation.
Taurine ^[^ ^357^, ^358^, ^359^, ^360^ ^]^	Suppression of RAAS, promotion of NO release, potent antioxidant effects, and enhanced H_2_S generation.	Increased vascular relaxation and flow‐mediated dilation.
NMN ^[^ ^150^, ^361^, ^362^, ^363^ ^]^	Activation of sirtuins, improved endothelial function, and reduced oxidative stress, enhances NO bioavailability and others.	Mitigates vascular dysfunction, improves vascular dilation, reduces blood pressure, restores neurovascular coupling and cognitive functions.
Transient expression of a cocktail of mRNAs encoding Oct4, Sox2, Klf4, c‐Myc, Lin28, and NANOG ^[^ ^364^ ^]^	Epigenetic reset.	Reversed cellular aging.

### 6.2 Biomarkers for Vascular Aging Prediction

Biomarkers provide insights into individual risk factors and guide personalized treatment approaches. As described by The American Federation for Aging Research, a successful biomarker of aging should predict the rate of aging of a person and determine the position of an individual in their lifespan more accurately than chronological age. Moreover, the assay used to measure such biomarker should be minimally invasive, and the biomarker should be applicable in both model organisms and humans, as drug candidates testing is conducted on animals. In the context of vascular aging, various kinds of biological parameters were suggested as possible biomarker candidates, including telomere length,^[^ ^365^ ^]^ inflammatory cytokines (e.g., IL‐6),^[^ ^366^ ^]^ Klotho,^[^ ^367^ ^]^ soluble VCAM‐1 (sVCAM‐1),^[^ ^368^ ^]^ circulating miR‐29a‐3p, miR‐574‐3p and miR‐574‐5p,^[^ ^369^ ^]^ and levels of soluble von Willebrand Factor (vWF).^[^ ^370^ ^]^ Among additional candidates, red blood cell distribution width (RDW) has also been identified, together with endothelial‐specific cfDNA. In fact, elevated RDW has been linked to increased cardiovascular risk,^[^ ^371^ ^]^ whereas high levels of circulating cfDNA, which also reflect vascular injury and aging processes, have been associated with various ARDs, such as CVDs, diabetes, and chronic kidney disease.^[^ ^372^, ^373^, ^374^ ^]^ Moreover, the methylation patterns of cfDNA have been shown to correlate with age‐related changes in vascular function, providing a non‐invasive means to assess biological age and vascular health.^[^ ^375^, ^376^ ^]^ Altogether, these biomarkers provide valuable insights into the mechanisms of vascular aging and can help in the development of targeted interventions to improve cardiovascular health in aging populations. However, despite these promising candidates, most vascular aging biomarkers remain in early validation phases and lack standardized measurement protocols or cross‐population validation in large, ethnically diverse cohorts. The field requires systematic validation studies that demonstrate not only predictive accuracy but also clinical utility—whether modifying these biomarkers through interventions translates to improved vascular outcomes and healthspan extension. Until such comprehensive validation is achieved, their translation from research tools to clinical decision‐making remains limited.^[^ ^377^ ^]^

Omics have the potential to transform the field of vascular aging by providing insights into the underlying molecular mechanisms and opening the path for personalized treatment strategies. Genomic data can help to identify genetic variants that represent risk factors for accelerated vascular aging. For example, genome‐wide association studies have been instrumental in this regard, revealing specific polymorphisms of apolipoprotein E that heighten the risk of developing age‐related vascular conditions by influencing lipid metabolism.^[^ ^378^ ^]^ Analyses of proteomic profiles of vascular tissues and biofluids can help to identify biomarkers, elucidate molecular mechanisms, and discover potential therapeutic targets for improving vascular aging. For instance, proteomic analysis of symptomatic carotid plaques revealed specific molecular signatures that could serve as biomarkers for atherosclerosis, such as MMP9, cathepsin D, and galectin‐3‐binding protein.^[^ ^379^ ^]^ Similarly, the proteomic signature of human plasma can be used to study how different organs are aging and whether organ‐specific accelerated aging confers increased risk for the development of organ‐specific diseases.^[^ ^380^ ^]^ Epigenomic analyses instead can allow for the identification of epigenetic modifications that are linked with vascular function and aging. For example, differentially methylated DNA regions in the BRCA1 and CRISP2 genes have been observed in aortic tissues from individuals with atherosclerosis, suggesting that epigenetic drift may contribute to the progression of CVDs with age.^[^ ^381^ ^]^ Despite these promising advances, the multidimensional nature of vascular aging requires fundamental integration within a comprehensive multi‐omics framework. Current single‐omics approaches provide only partial insights. However, multi‐omics integration faces significant technical challenges, including data heterogeneity across platforms, variable measurement scales, and a difficult connection between datasets. Furthermore, achieving systems‐level understanding of vascular aging mechanisms requires advanced computational and in vitro approaches that can identify causal relationships rather than mere correlations across genomic, epigenomic, transcriptomic, proteomic, and metabolomic data.

### 6.3 AI and Machine Learning in Aging Research

The large amount of data produced by modern omics technologies (e.g., genomics, transcriptomics, and proteomics), also in the context of aging, requires advanced computational methods to be processed and interpreted. In this context, AI appears to be a promising tool. AI, and particularly machine learning (ML), is commonly used to extract knowledge from high‐throughput data. For instance, ML can identify complex and nonlinear associations between omics data and phenotypes, allowing for the identification of new biomarkers through the joint analysis of a huge number of covariates.^[^ ^382^ ^]^ This approach has been employed, for example, to correlate methylation data with age or age‐related processes (i.e., aging clocks), revealing that a predicted age surpassing chronological age is often predictive of age‐related disorders.^[^ ^383^ ^]^ In the context of aging, a Bayesian Network‐augmented naïve Bayes algorithm was used to classify whether a gene of *C. elegans* is related to longevity based on a list of gene ontology terms describing each gene. Several other studies used ML to prioritize aging‐associated genes based on different sets of features, often extracted from the analysis of biological networks, such as functional networks.^[^ ^384^ ^]^

In biomedical research, it is important to interpret ML models to extract insights that can enable novel discoveries and allow for prospective validations (e.g., by in vitro experiments), a crucial step that has often shown how ML can pinpoint molecules that later prove to be active in lab tests.^[^ ^385^ ^]^ Sayed et al. identified CXCL9 as a key predictor of a deep learning model for chronological age and found that silencing it reversed early senescence. Hence, by improving the understanding of aging mechanisms, AI helps identify new therapeutic targets and discovering new anti‐aging drugs.^[^ ^386^ ^]^ Additionally, ML can also support virtual screening of compounds (e.g., by predicting their behavior and interactions from 3D images).^[^ ^385^ ^]^ In this context, Yu et al. implemented a full pipeline of bioinformatics exploiting both supervised and unsupervised ML to identify cardiac age‐related genes and possible anti‐aging drugs.^[^ ^387^ ^]^ Finally, ML can serve aging research also by automatizing time‐consuming manual processes, such as using deep learning to accelerate DNA damage quantification from imaging flow cytometry ^[^ ^388^ ^]^ or processing wearable device data used in elderly care services.^[^ ^389^ ^]^

Overall, AI technologies hold great promise for uncovering insights that could promote healthy aging. An important limitation is, however, the availability of large and unbiased datasets. This motivates the ongoing effort to collect comprehensive collections housing information about aging‐specific biomarkers or curated aging‐related multi‐omic data, such as the Digital Aging Atlas, HAGR, AgeFactDB, AGEMAP, and the Aging Atlas.^[^ ^390^ ^]^

### 6.4 Longevity Medicine and Aging Clocks

Longevity medicine is a branch of precision medicine promoting long and healthy lives.^[^ ^391^ ^]^ AI applications contributing to the field of longevity research include, for instance, deep learning‐based imaging for the diagnosis of ARDs,^[^ ^392^ ^]^ the so called Internet of Things for monitoring and supporting elderly people,^[^ ^389^ ^]^ and AI‐based coaching to support healthy lifestyle,^[^ ^393^ ^]^ anti‐aging drug development,^[^ ^394^ ^]^ and precision medicine for ARDs.^[^ ^395^ ^]^ Several specialized companies and most biotech industries are engaged in this field.^[^ ^396^ ^]^

Aging clocks are a fundamental tool of longevity medicine, as biomarkers of biological aging are necessary to assess the effectiveness of health span extending interventions.^[^ ^391^, ^397^ ^]^ They are based on ML models trained to predict the chronological age, but are expected to track also biological aging. Therefore, they are tested as predictor of aging‐related outcomes, such as all‐cause mortality, health span, physical functioning, and various ARDs, generally showing a significant correlation with these outcomes.^[^ ^398^ ^]^ A plethora of aging clocks has been developed using different training datasets, inputs, targets, and training algorithms. Several have been trained on methylation data,^[^ ^383^ ^]^ while others on cytokine data,^[^ ^386^ ^]^ metabolomic profiling,^[^ ^399^ ^]^ transcriptomics,^[^ ^400^ ^]^ proteomics,^[^ ^401^ ^]^ imaging,^[^ ^402^ ^]^ and clinical parameters.^[^ ^403^ ^]^ Some researchers have also considered developing aging clocks trained on healthspan indicators different from chronological age. GrimAge ^[^ ^404^ ^]^ uses time‐to‐death due to all‐cause mortality data; PhenoAge^[^ ^405^ ^]^ relies on a composite clinical measure of phenotypic age; and DunedinPACE ^[^ ^406^ ^]^ combines the longitudinal changes in 19 biomarkers. These clocks improve the predictive accuracy for their respective training outcomes and can disclose biomolecules or pathways more specifically associated with them. According to Rutledge et al., they are also likely to be more robustly associated with physiological aging.^[^ ^407^ ^]^ Aging clocks also present fundamental limitations. Faul et al. report that they often have less predictive power than other phenotypic traits that are usually much easier and cheaper to ascertain, such as demographics and healthy behaviors.^[^ ^398^ ^]^ Rutledge et al. highlight that aging clocks correlate only mildly with each other, suggesting that each captures only some aspects of aging. They also point out how often clocks fail to accurately estimate biological aging, and argue that moving beyond the traditional training on chronological age is necessary. However, there is currently no agreement on which measure of biological age should be used. In this context, more attention could be devoted to multi‐task ML approaches, which model multiple correlated outcomes simultaneously.^[^ ^407^ ^]^ For example, Huang et al. implemented a two‐task deep learning model that simultaneously extracts distinct identity‐ and age‐related features from face features,^[^ ^408^ ^]^ while Pan et al. showed that joint learning can improve both age and cognitive decline predictions.^[^ ^409^ ^]^ Another limitation of current research on aging clocks is that most studies focus on a single data type, such as methylation or transcriptomics. Integrative approaches combining multi‐omic data ^[^ ^410^ ^]^ remain rare, despite their potential to reveal novel biomarkers and provide deeper insights into biological aging.^[^ ^411^ ^]^

## 7 Conclusion

Vascular aging plays a central role in the onset and progression of ARDs, including CVDs and neurodegenerative conditions. This process, which is characterized by a combination of cellular and molecular changes, leads to the deterioration of vascular integrity, including increased arterial stiffness, impaired angiogenesis, and altered vascular permeability. For this reason, understanding vascular aging and developing strategies to counteract it is critical for enhancing the quality of life in the aging population, although the translation from bench to bedside shows substantial challenges that demand critical evaluation.

The selection of appropriate experimental models continues to present crucial obstacles in vascular aging research. While animal models have provided mechanistic insights into systemic aging processes, their limited translational validity to human vascular pathophysiology has become increasingly apparent. In vitro systems, though offering controlled environments for studying human‐specific processes, often fail to recapitulate the complex multicellular interactions and hemodynamic forces that characterize aging vasculature in vivo. This modeling gap has contributed to the disappointing translation rate of promising preclinical findings to clinical efficacy.

In recent years, candidate therapeutic strategies to mitigate vascular aging have been identified. Pharmacological approaches, such as senolytic administration and the modulation of nutrient‐sensing pathways (e.g., mTOR and AMPK), have demonstrated promising results in preclinical studies. Similarly, lifestyle interventions, including regular exercise and dietary changes, have been shown to enhance vascular health by reducing oxidative stress, improving endothelial function, and promoting vascular repair. Despite promising preclinical evidence, some of these interventions face critical challenges, including unclear mechanisms of action, lack of standardized biomarkers for monitoring therapeutic efficacy, inter‐individual variability and concerns regarding off‐target effects and long‐term safety profiles. Overall, these emerging pharmacological and lifestyle interventions offer a promising foundation for translating vascular rejuvenation into effective, personalized therapies.

Despite their promise, vascular‐aging interventions must overcome key hurdles: refining multi‐omics integration to reveal causality beyond correlations; advancing aging clocks through cross‐population validation and clinical outcome linkage; and standardizing vascular‐specific biomarkers for reliable monitoring.

## Conflict of Interest

The authors declare no conflict of interest.

## References

[1] Blueprint – Blueprint Bryan Johnson [Internet]. Available from: https://blueprint.bryanjohnson.com/.

[2] C. López‐Otín , M. A. Blasco , L. Partridge , M. Serrano , G. Kroemer , Cell 2023, 186, 243. 36599349 10.1016/j.cell.2022.11.001

[3] J. Guo , X. Huang , L. Dou , M. Yan , T. Shen , W. Tang , et al., Signal Transduct. Target Ther. 2022, 7, 391. 36522308 10.1038/s41392-022-01251-0 PMC9755275

[4] Ageing and health [Internet]. Available from: https://www.who.int/news‐room/fact‐sheets/detail/ageing‐and‐health.

[5] J. Kalucka , L. P. M. H. de Rooij , J. Goveia , K. Rohlenova , S. J. Dumas , E. Meta , N. V. Conchinha , F. Taverna , L.‐A. Teuwen , K. Veys , M. García‐Caballero , S. Khan , V. Geldhof , L. Sokol , R. Chen , L. Treps , M. Borri , P. de Zeeuw , C. Dubois , T. K. Karakach , K. D. Falkenberg , M. Parys , X. Yin , S. Vinckier , Y. Du , R. A. Fenton , L. Schoonjans , M. Dewerchin , G. Eelen , B. Thienpont , et al., Cell 2020, 180, 764.e20. 32059779 10.1016/j.cell.2020.01.015

[6] S. Rafii , J. M. Butler , B. S. Ding , Nature 2016, 529, 316. 26791722 10.1038/nature17040 PMC4878406

[7] H. G. Augustin , G. Y. Koh , Science 2017, 357, aal2379. 10.1126/science.aal2379 28775214

[8] P. M. Nilsson , P. Boutouyrie , S. Laurent , Vasc. Aging. Hypertens. 2009, 54, 3. 10.1161/HYPERTENSIONAHA.109.129114 19487587

[9] S. Pinho , P. S. Frenette , Nat. Rev. Mol. Cell Biol. 2019, 20, 303. 30745579 10.1038/s41580-019-0103-9 PMC6483843

[10] L. Larsson , H. Degens , M. Li , L. Salviati , Y. I.l Lee , W. Thompson , J. L. Kirkland , M. Sandri , Physiol. Rev. 2019, 99, 427. 30427277 10.1152/physrev.00061.2017 PMC6442923

[11] S. Laurent , J. Hypertens. 2012, 30, S3. 23124102 10.1097/HJH.0b013e328353e501

[12] B. Jani , C. Rajkumar , Postgrad. Med. J. 2006, 82, 357. 16754702 10.1136/pgmj.2005.036053 PMC2563742

[13] M. D. Herrera , C. Mingorance , R. Rodríguez‐Rodríguez , M. Alvarez de Sotomayor , Ageing Res. Rev. 2010, 9, 142. 19619671 10.1016/j.arr.2009.07.002

[14] P. Lacolley , V. Regnault , S. Laurent , Arterioscler Thromb. Vasc. Biol. 2020, 40, 1055. 32075419 10.1161/ATVBAHA.119.313129

[15] J. Chen , U. Sivan , S. L. Tan , L. Lippo , J. De Angelis , R. Labella , A. Singh , A. Chatzis , S. Cheuk , M. Medhghalchi , J. Gil , G. Hollander , B. D. Marsden , R. Williams , S. K. Ramasamy , A. P. Kusumbe , Sci. Adv. 2021, 7, abd7819. 10.1126/sciadv.abd7819 PMC7857692 33536212

[16] D. G. Le Couteur , R. Fraser , V. C. Cogger , A. J. McLean , Lancet. Lond. Engl. 2002, 359, 1612. 10.1016/S0140-6736(02)08524-0 12047987

[17] H. G. Augustin , G. Y. Koh , Cell 2024, 5, 4833. 10.1016/j.cell.2024.07.012 39241746

[18] S. I. Bloom , M. T. Islam , L. A. Lesniewski , A. J. Donato , Nat. Rev. Cardiol. 2023, 20, 38. 35853997 10.1038/s41569-022-00739-0 PMC10026597

[19] Z. Ungvari , S. Tarantini , A. J. Donato , V. Galvan , A. Csiszar , Circ. Res. 2018, 123, 849. 30355080 10.1161/CIRCRESAHA.118.311378 PMC6248882

[20] R. Oakley , B. Tharakan , Aging Dis. 2014, 5, 114. 24729937 10.14336/AD.2014.0500114 PMC3966670

[21] P. Guimaraes Cunha , P. Boutouyrie , P. M Nilsson , S. Laurent , Curr. Hypertens. Rev. 2017, 13, 8. 28412914 10.2174/1573402113666170413094319

[22] X. Theodoridis , M. Chourdakis , A. Papaemmanouil , S. Chaloulakou , A. V. Georgakou , G. Chatzis , A. Triantafyllou , Life 2024, 14, 267. 38398776 10.3390/life14020267 PMC10890697

[23] R. M. Bruno , P. M. Nilsson , G. Engström , B. N. Wadström , J.‐P. Empana , P. Boutouyrie , S. Laurent , Hypertension 2020, 76, 1616. 32895017 10.1161/HYPERTENSIONAHA.120.14971

[24] A. J. Donato , D. R. Machin , L. A. Lesniewski , Circ. Res. 2018, 123, 825. 30355078 10.1161/CIRCRESAHA.118.312563 PMC6207260

[25] B. Ahmed , A. A. Rahman , S. Lee , R. Malhotra , Int. J. Mol. Sci. 2024, 25, 11188. 39456971 10.3390/ijms252011188 PMC11508873

[26] P. Libby , J. E. Buring , L. Badimon , G. K. Hansson , J. Deanfield , M. S. Bittencourt , L. Tokgözoglu , E. F. Lewis , Nat. Rev. Dis. Primer. 2019, 5, 56. 10.1038/s41572-019-0106-z 31420554

[27] K. J. Williams , I. Tabas , Arterioscler Thromb. Vasc. Biol. 1995, 15, 551. 7749869 10.1161/01.atv.15.5.551 PMC2924812

[28] P. Conti , Y. Shaik‐Dasthagirisaeb , Cent. Eur. J. Immunol. 2015, 40, 380. 26648785 10.5114/ceji.2015.54603 PMC4655391

[29] U. Ikeda , K. Matsui , Y. Murakami , K. Shimada , Clin. Cardiol. 2002, 25, 143. 12000070 10.1002/clc.4960250403 PMC6654294

[30] F. K. Swirski , R. Weissleder , M. J. Pittet , Arterioscler Thromb. Vasc. Biol. 2009, 29, 1424. 19372462 10.1161/ATVBAHA.108.180521 PMC2746262

[31] H. Ait‐Oufella , A. P. Sage , Z. Mallat , A. Tedgui , Circ. Res. 2014, 114, 1640. 24812352 10.1161/CIRCRESAHA.114.302761

[32] S. Lee , B. Bartlett , G. Dwivedi , Int. J. Mol. Sci. 2020, 21, 9322. 33297441 10.3390/ijms21239322 PMC7731312

[33] S. Brauner , X. Jiang , G. E. Thorlacius , A. M. Lundberg , T. Östberg , Z.‐Q. Yan , V. K. Kuchroo , G. K. Hansson , M. Wahren‐Herlenius , Cardiovasc. Res. 2018, 114, 158. 29016728 10.1093/cvr/cvx181

[34] W. Li , Y. Chen , S. Li , X. Guo , W. Zhou , Q. Zeng , Y. Liao , Y. Wei , Am. J. Transl. Res. 2014, 6, 678. 25628779 PMC4297336

[35] W. Martinet , M. W. M. Knaapen , G. R. Y. De Meyer , A. G. Herman , M. M. Kockx , Circulation 2002, 106, 927. 12186795 10.1161/01.cir.0000026393.47805.21

[36] R. Marfella , C. D. Filippo , M. T. Laieta , R. Vestini , M. Barbieri , P. Sangiulo , B. Crescenzi , F. Ferraraccio , F. Rossi , M. D'Amico , G. Paolisso , J. Gerontol. A Biol. Sci. Med. Sci. 2008, 63, 200. 18314458 10.1093/gerona/63.2.200

[37] M. Kitada , Y. Ogura , D. Koya , Aging 2016, 8, 2290. 27744418 10.18632/aging.101068 PMC5115889

[38] T. Kuret , S. Sodin‐Šemrl , T. Kuret , S. Sodin‐Šemrl , IntechOpen 2021.

[39] F.‐X. Guo , Q. Wu , P. Li , L. Zheng , S. Ye , X.‐Y. Dai , C.‐M. Kang , J.‐B. Lu , B.‐M. Xu , Y.‐J. Xu , L. Xiao , Z.‐F. Lu , H.‐L. Bai , Y.‐W. Hu , Q. Wang , Cell Death Differ. 2019, 26, 1670. 30683918 10.1038/s41418-018-0235-z PMC6748100

[40] R. P. M. Snijckers , A. C. Foks , Front. Immunol. 2024, 15, 1350471. 38686373 10.3389/fimmu.2024.1350471 PMC11056569

[41] A. N. Kucher , I. A. Koroleva , M. S. Nazarenko , Biomedicines 2025, 13, 593. 40149570 10.3390/biomedicines13030593 PMC11940622

[42] J. Golledge , P. Norman , Arterioscler Thromb. Vasc. Biol. 2010, 30, 1075. 20484703 10.1161/ATVBAHA.110.206573 PMC2874982

[43] M. J. Cho , M. R. Lee , J. G. Park , Exp. Mol. Med. 2023, 55, 2519. 38036736 10.1038/s12276-023-01130-w PMC10766996

[44] P. Libby , R. Sidlow , A. E. Lin , D. Gupta , L. W. Jones , J. Moslehi , et al., Clonal Hematopoiesis. JACC. 2019, 74, 567. 31345432 10.1016/j.jacc.2019.06.007 PMC6681657

[45] J. J. Fuster , S. MacLauchlan , M. A. Zuriaga , M. N. Polackal , A. C. Ostriker , R. Chakraborty , C.‐L. Wu , S. Sano , S. Muralidharan , C. Rius , J. Vuong , S. Jacob , V. Muralidhar , A. A. B. Robertson , M. A. Cooper , V. Andrés , K. K. Hirschi , K. A. Martin , K. Walsh , Science 2017, 355, 842. 28104796 10.1126/science.aag1381 PMC5542057

[46] J. Li , C. Wang , J. Liu , Y. Yu , Y. Liu , Q.i Peng , H. Liu , X. Guan , Mol. Biol. Rep. 2021, 48, 3785. 33987748 10.1007/s11033-021-06370-5 PMC8117808

[47] S. Wu , Y. Xu , R. Zheng , J. Lu , M. Li , L. Chen , et al., Lancet. Reg. Health West. Pac. 2022, 20, 100350. 35036974 10.1016/j.lanwpc.2021.100350 PMC8749453

[48] A. Harvey , A. C. Montezano , R. M. Touyz , J. Mol. Cell. Cardiol. 2015, 83, 112. 25896391 10.1016/j.yjmcc.2015.04.011 PMC4534766

[49] D. H. J. Thijssen , S. E. Carter , D. J. Green , J. Physiol. 2016, 594, 2275. 26140618 10.1113/JP270597 PMC4933112

[50] A. Durante , A. Mazzapicchi , M. Baiardo Redaelli , Int. J. Mol. Sci. 2024, 25, 13294. 39769057 10.3390/ijms252413294 PMC11677602

[51] C. E. Baggeroer , F. E. Cambronero , N. A. Savan , A. L. Jefferson , M. M. Santisteban , Hypertens. Dallas Tex 1979 2024, 81, 34. 10.1161/HYPERTENSIONAHA.123.19939 PMC10840624 37732479

[52] P. Toth , S. Tarantini , A. Csiszar , Z. Ungvari , Am J Physiol.‐Heart Circ. Physiol. 2017, 312, H1. 27793855 10.1152/ajpheart.00581.2016 PMC5283909

[53] J. Flores , C. Pena , K. Nugent , J. Bras. Nefrol. 2019, 47, 202440192. 10.1590/2175-8239-JBN-2024-0192en PMC12129482 40456017

[54] W. Wang , G. Li , J. Ma , X. Fan , J. Lu , Q. Sun , J. Yao , Q. He , Front. Pharmacol. 2024, 15, 1346905. 38405666 10.3389/fphar.2024.1346905 PMC10885812

[55] S. Y. Sinkler , S. S. Segal , Am. J. Physiol.‐Heart Circ. Physiol. 2014, 307, H830. 25015968 10.1152/ajpheart.00368.2014 PMC4166744

[56] M. G. Scioli , A. Bielli , G. Arcuri , A. Ferlosio , A. Orlandi , Vasc Cell 2014, 6, 19. 25243060 10.1186/2045-824X-6-19 PMC4169693

[57] P. M. Vanhoutte , H. Shimokawa , M. Feletou , E. H. C. Tang , Acta Physiol. 2017, 219, 22. 10.1111/apha.12646 26706498

[58] T. F. Lüscher , C. M. Boulanger , Y. Dohi , Z. H. Yang , Hypertension 1992, 19, 117. 1737645 10.1161/01.hyp.19.2.117

[59] M. S. D'Souza , A. N. Luu , T. C. Guisinger , S. L. Seeley , R. A. Waldschmidt , S. Chrissobolis , J. Cardiovasc. Pharmacol. 2022, 80, 305. 35384894 10.1097/FJC.0000000000001272

[60] C. Savoia , D. Burger , N. Nishigaki , A. Montezano , R. M. Touyz , Expert. Rev. Mol. Med. 2011, 13, 11. 10.1017/S1462399411001815 21450123

[61] S. S. Hasan , A. Fischer , Cold Spring Harb Perspect. Med. 2023, 13, a041166. 35667708 10.1101/cshperspect.a041166 PMC9899647

[62] T. Leibing , C. Géraud , I. Augustin , M. Boutros , H. G. Augustin , J. G. Okun , C.‐D. Langhans , J. Zierow , S. A. Wohlfeil , V. Olsavszky , K. Schledzewski , S. Goerdt , P.‐S. Koch , Hepatology 2018, 68, 707. 29059455 10.1002/hep.29613 PMC6099291

[63] P.‐S. Koch , V. Olsavszky , F. Ulbrich , C. Sticht , A. Demory , T. Leibing , T. Henzler , M. Meyer , J. Zierow , S. Schneider , K. Breitkopf‐Heinlein , H. Gaitantzi , B. Spencer‐Dene , B. Arnold , K. Klapproth , K. Schledzewski , S. Goerdt , C. Géraud , Blood 2017, 129, 415. 27903529 10.1182/blood-2016-07-729822 PMC5291964

[64] V. Fleischhacker , F. Milosic , M. Bricelj , K. Kührer , K. Wahl‐Figlash , P. Heimel , A. Diendorfer , E. Nardini , I. Fischer , H. Stangl , P. Pietschmann , M. Hackl , R. Foisner , J. Grillari , M. Hengstschläger , S. Osmanagic‐Myers , Aging Cell 2024, 23, 14139. 10.1111/acel.14139 PMC11166365 38578073

[65] A. P. Kusumbe , S. K. Ramasamy , T. Itkin , M. A. Mäe , U. H. Langen , C. Betsholtz , T. Lapidot , R. H. Adams , Nature 2016, 532, 380. 27074508 10.1038/nature17638 PMC5035541

[66] R. Farahzadi , B. Valipour , S. Montazersaheb , E. Fathi , Front. Cell Dev. Biol. 2023, 11, 1162136. 37274742 10.3389/fcell.2023.1162136 PMC10235764

[67] C. Tan , P. R. Norden , W. Yu , T. Liu , N. Ujiie , S. K. Lee , X. Yan , Y. Dyakiv , K. Aoto , S. Ortega , I. G. De Plaen , V. Sampath , T. Kume , EMBO Rep. 2023, 24, 56030. 10.15252/embr.202256030 PMC10328078 37154714

[68] M. van Dinther , J. Bennett , G. D. Thornton , P. H. M. Voorter , A. Ezponda Casajús , A. Hughes , G. Captur , R. J. Holtackers , J. Staals , W. H. Backes , G. Bastarika , E. A. V. Jones , A. González , R. J. van Oostenbrugge , T. A. Treibel , Cerebrovasc. Dis. Extra 2023, 13, 18. 36646051 10.1159/000529067 PMC9939919

[69] P. G. Tickle , P. W. Hendrickse , H. Degens , S. Egginton , J. Physiol. 2020, 598, 1187. 32012275 10.1113/JP278975 PMC7154729

[70] M. Grunewald , S. Kumar , H. Sharife , E. Volinsky , A. Gileles‐Hillel , T. Licht , A. Permyakova , L. Hinden , S. Azar , Y. Friedmann , P. Kupetz , R. Tzuberi , A. Anisimov , K. Alitalo , M. Horwitz , S. Leebhoff , O. Z. Khoma , R. Hlushchuk , V. Djonov , R. Abramovitch , J. Tam , E. Keshet , Science 2021, 373, abc8479. 10.1126/science.abc8479 34326210

[71] L. Gifre‐Renom , M. Daems , A. Luttun , E. A. V. Jones , Int. J Mol Sci. 2022, 23, 1477. 35163400 10.3390/ijms23031477 PMC8836165

[72] L. M. Becker , S. H. Chen , J. Rodor , L. P. M. H. de Rooij , A. H. Baker , P. Carmeliet , Cardiovasc. Res. 2023, 119, 6. 35179567 10.1093/cvr/cvac018 PMC10022871

[73] A. C. Yang , M. Y. Stevens , M. B. Chen , D. P. Lee , D. Stähli , D. Gate , K. Contrepois , W. Chen , T. Iram , L. Zhang , R. T. Vest , A. Chaney , B. Lehallier , N. Olsson , H. du Bois , R. Hsieh , H. C. Cropper , D. Berdnik , L. Li , E. Y. Wang , G. M. Traber , C. R. Bertozzi , J. Luo , M. P. Snyder , J. E. Elias , S. R. Quake , M. L. James , T. Wyss‐Coray , Nature 2020, 583, 425. 32612231 10.1038/s41586-020-2453-z PMC8331074

[74] M. W. Hetzer , S. Bersini , Cold Spring Harb Perspect. Med. 2022, 12, a041180. 35101902 10.1101/cshperspect.a041180 PMC9310951

[75] I. Georgieva , J. Tchekalarova , D. Iliev , R. Tzoneva , Int. J. Mol. Sci. 2023, 24, 11344. 37511104 10.3390/ijms241411344 PMC10379128

[76] C. Janus , C. Hernandez , V. deLelys , H. Roder , H. Welzl , Methods Mol. Biol. Clifton. NJ 2016, 1438, 311. 10.1007/978-1-4939-3661-8_18 27150098

[77] S. Al‐Bachari , J. H. Naish , G. J. M. Parker , H. C. A. Emsley , L. M. Parkes , Front. Physiol. 2020, 11, 593026. 33414722 10.3389/fphys.2020.593026 PMC7784911

[78] L. Zhao , Z. Li , J. S. L. Vong , X. Chen , H. M. Lai , L. Y. C. Yan , J. Huang , S. K. H. Sy , X. Tian , Y. Huang , H. Y. E. Chan , H. C. So , W. L. Ng , Y. Tang , W. J. Lin , V. C. T. Mok , H. Ko , Nat Commun. 2020, 4, 4413. 10.1038/s41467-020-18249-3 PMC7474063 32887883

[79] M. Fisher , S. French , P. Ji , R. C. Kim , Stroke 2010, 41, 2782. 21030702 10.1161/STROKEAHA.110.593657 PMC3079284

[80] P. A. Yates , V. L. Villemagne , K. A. Ellis , P. M. Desmond , C. L. Masters , C. C. Rowe , Front Neurol. 2014, 4, 205. 24432010 10.3389/fneur.2013.00205 PMC3881231

[81] Z. B. Bulwa , G. C. Ward , O. N. Kramer , B. Rao , M. Wichter , Am. J. Case Rep. 2016, 17, 860. 27853131 10.12659/AJCR.900498 PMC5115614

[82] M. Damodarasamy , R. B. Vernon , J. L. Pathan , C. D. Keene , A. J. Day , W. A. Banks , M. J. Reed , Fluids Barriers CNS 2020, 29, 60. 10.1186/s12987-020-00219-y PMC7525948 32993718

[83] A. Mammoto , M. Muyleart , T. Mammoto , Aging 2019, 11, 89. 30612120 10.18632/aging.101722 PMC6339783

[84] K. E. Coffman , M. G. Boeker , A. R. Carlson , B. D. Johnson , Physiol. Rep. 2018, 6, 13834. 10.14814/phy2.13834 PMC6119697 30175463

[85] B. J. Taylor , B. D. Johnson , Semin Respir. Crit. Care Med. 2010, 31, 528. 20941654 10.1055/s-0030-1265894 PMC3919503

[86] R. Vidal , J. U. G. Wagner , C. Braeuning , C. Fischer , R. Patrick , L. Tombor , M. Muhly‐Reinholz , D. John , M. Kliem , T. Conrad , N. Guimarães‐Camboa , R. Harvey , S. Dimmeler , S. Sauer , JCI Insight 2019, 4, 131092. 31723062 10.1172/jci.insight.131092 PMC6948853

[87] M. E. Pickering , Int. J. Mol. Sci. 2021, 22, 9102.34445808

[88] S. J. Prior , A. S. Ryan , J. B. Blumenthal , J. M. Watson , L. I. Katzel , A. P. Goldberg , J. Gerontol. Ser. A 2016, 71, 1096. 10.1093/gerona/glw017 PMC5007615 26888434

[89] L. Wang , P. Jia , Y. Shan , Y. Hao , X. Wang , Y. Jiang , Y. Yuan , Q. Du , H. Zhang , F. Yang , W. Zhang , M. Sheng , Y. Xu , Mol. Med. Rep. 2017, 16, 6642. 28901524 10.3892/mmr.2017.7451 PMC5865796

[90] A. Gaudio , A. Xourafa , L. Zanoli , R. Rapisarda , A. Catalano , S. S. Signorelli , P. Castellino , Sci. Rep. 2020, 10, 19421. 33173083 10.1038/s41598-020-76427-1 PMC7656252

[91] V. C. Cogger , D. Svistounov , A. Warren , S. Zykova , R. G. Melvin , S. M. Solon‐Biet , J. N. O'Reilly , A. C. McMahon , J. W. O. Ballard , R. De Cabo , D. G. Le Couteur , M. Lebel , J. Gerontol. A Biol. Sci. Med. Sci. 2014, 69, 1076. 24149428 10.1093/gerona/glt169 PMC4158411

[92] R. Maeso‐Díaz , M. Ortega‐Ribera , E. Lafoz , J. J. Lozano , A. Baiges , R. Francés , A. Albillos , C. Peralta , J. C. García‐Pagán , J. Bosch , V. C. Cogger , J. Gracia‐Sancho , Aging Dis. 2019, 10, 684. 31440376 10.14336/AD.2019.0127 PMC6675529

[93] L. Baiocchi , S. Glaser , H. Francis , L. Kennedy , E. Felli , G. Alpini , J. Gracia‐Sancho , Hepatol. Commun. 2021, 5, 1125. 34278165 10.1002/hep4.1725 PMC8279468

[94] A. Denic , R. J. Glassock , Adv. Chronic Kidney Dis. 2016, 23, 19. 26709059 10.1053/j.ackd.2015.08.004 PMC4693148

[95] A. R. Chade , Hypertension 2017, 69, 551. 28193706 10.1161/HYPERTENSIONAHA.116.08319 PMC5344725

[96] M. S. Hommos , R. J. Glassock , J. Am. Soc. Nephrol. 2017, 28, 2838. 28790143 10.1681/ASN.2017040421 PMC5619977

[97] A. J. Dixon , P. Osei‐Owusu , Front. Physiol. 2023, 14, 1141094. 37179824 10.3389/fphys.2023.1141094 PMC10167050

[98] H. Ren , Z. Guo , Y. Liu , C. Song , Aging Dis 2022, 13, 852. 35656114 10.14336/AD.2021.1110 PMC9116915

[99] M. T. Sweetwyne , J. W. Pippin , D. G. Eng , K. L. Hudkins , Y. A. Chiao , M. D. Campbell , D. J. Marcinek , C. E. Alpers , H. H. Szeto , P. S. Rabinovitch , S. J. Shankland , Kidney Int. 2017, 91, 1126. 28063595 10.1016/j.kint.2016.10.036 PMC5392164

[100] H. H. Szeto , S. Liu , Y. Soong , D. Wu , S. F. Darrah , F.‐Y. Cheng , Z. Zhao , M. Ganger , C. Y. Tow , S. V. Seshan , J. Am. Soc. Nephrol. 2011, 22, 1041. 21546574 10.1681/ASN.2010080808 PMC3103724

[101] H. H. Szeto , S. Liu , Y. Soong , N. Alam , G. T. Prusky , S. V. Seshan , Kidney Int. 2016, 90, 997. 27519664 10.1016/j.kint.2016.06.013

[102] T. J. Guzik , R. M. Touyz , Hypertension 2017, 70, 660. 28784646 10.1161/HYPERTENSIONAHA.117.07802

[103] J. Zhuang , L. Zhang , S. Dai , L. Cui , C. Guo , L. Sloofman , J. Yang , Sci. Rep. 2019, 9, 6220. 30996271 10.1038/s41598-019-42485-3 PMC6470208

[104] B. G. Childs , D. J. Baker , T. Wijshake , C. A. Conover , J. Campisi , J. M. van Deursen , Science 2016, 354, 472. 27789842 10.1126/science.aaf6659 PMC5112585

[105] V. N. Gladyshev , S. B. Kritchevsky , S. G. Clarke , A. M. Cuervo , O. Fiehn , J. P. de Magalhães , T. Mau , M. Maes , R. L. Moritz , L. J. Niedernhofer , E. Van Schaftingen , G. J. Tranah , K. Walsh , Y. Yura , B. Zhang , S. R. Cummings , Nat. Aging 2021, 1, 1096. 36846190 10.1038/s43587-021-00150-3 PMC9957516

[106] Z. Ungvari , A. Podlutsky , D. Sosnowska , Z. Tucsek , P. Toth , F. Deak , T. Gautam , A. Csiszar , W. E. Sonntag , J. Gerontol. A Biol. Sci. Med. Sci. 2013, 68, 1443. 23689827 10.1093/gerona/glt057 PMC3814240

[107] M. Durik , M. Kavousi , I. van der Pluijm , A. Isaacs , C. Cheng , K. Verdonk , A. E. Loot , H. Oeseburg , U. M. Bhaggoe , F. Leijten , R. van Veghel , R. de Vries , G. Rudez , R. Brandt , Y. R. Ridwan , E. D. van Deel , M. de Boer , D. Tempel , I. Fleming , G. F. Mitchell , G. C. Verwoert , K. V. Tarasov , A. G. Uitterlinden , A. Hofman , H. J. Duckers , C. M. van Duijn , B. A. Oostra , J. C. M. Witteman , D. J. Duncker , A. H. J. Danser , et al., Circulation 2012, 126, 468. 22705887 10.1161/CIRCULATIONAHA.112.104380 PMC3430727

[108] J. W. Shay , W. E. Wright , FEBS Lett. 2010, 584, 3819. 20493857 10.1016/j.febslet.2010.05.026 PMC3370416

[109] R. G. Morgan , A. J. Donato , A. E. Walker , Am. J. Physiol.‐Heart Circ. Physiol. 2018, 315, H1. 29547021 10.1152/ajpheart.00008.2018 PMC6087771

[110] S. I. Bloom , Y. Liu , J. R. Tucker , M. D. T. Islam , D. R. Machin , H. Abdeahad , T. G. Thomas , R. C. Bramwell , L. A. Lesniewski , A. J. Donato , Aging Cell 2023, 22, 13875. 10.1111/acel.13875 PMC10410008 37259606

[111] L. Lipskaia , M. Breau , C. Cayrou , D. Churikov , L. Braud , J. Jacquet , E. Born , C. Fouillade , S. Curras‐Alonso , S. Bauwens , F. Jourquin , F. Fiore , R. Castellano , E. Josselin , C. Sánchez‐Ferrer , G. Giovinazzo , C. Lachaud , E. Gilson , I. Flores , A. Londono‐Vallejo , S. Adnot , V. Géli , EMBO Rep. 2024, 25, 1650. 38424230 10.1038/s44319-023-00041-1 PMC10933469

[112] M. Vasa , K. Breitschopf , A. M. Zeiher , S. Dimmeler , Circ Res. 2000, 87, 540. 11009557 10.1161/01.res.87.7.540

[113] Z. Gao , R. B. Santos , J. Rupert , R. Van Drunen , Y. Yu , K. Eckel‐Mahan , M. G. Kolonin , Aging Cell 2024, 23, 14138. 10.1111/acel.14138 PMC11296101 38475941

[114] K. Ait‐Aissa , L. E. Norwood‐Toro , J. Terwoord , M. Young , L. A. Paniagua , S. N. Hader , W. E. Hughes , J. C. Hockenberry , J. E. Beare , J. Linn , T. Kohmoto , J. Kim , D. H. Betts , A. J. LeBlanc , D. D. Gutterman , A. M. Beyer , Function. 2022, 1, zqac043. 10.1093/function/zqac043 PMC9508843 36168588

[115] H. Xu , S. Li , Y. S. Liu , Front Cell Dev Biol 2021. 10.3389/fcell.2021.699374 PMC8273304 34262910

[116] A. Nguyen , F. Leblond , M. Mamarbachi , S. Geoffroy , E. Thorin , Oxid. Med. Cell. Longev. 2016, 2016, 8627384. 26989455 10.1155/2016/8627384 PMC4771915

[117] G. A. Challen , D. Sun , M. Jeong , M. Luo , J. Jelinek , A. Vasanthakumar , A. Meissner , J.‐P. Issa , L. Godley , W. Li , M. A. Goodell , Blood 2011, 118, 386.

[118] J. Cardiovasc. Aging 2021, 1, null.

[119] J. Jin , Y. Liu , L. Huang , H. Tan , Rev. Cardiovasc. Med. 2019, 20, 19. 31184092 10.31083/j.rcm.2019.01.3189

[120] J. Lähteenvuo , A. Rosenzweig , D. Sinclair , B. North , Circ. Res. 2012, 110, 1252.22539758

[121] C. Cantó , J. Auwerx , Trends Endocrinol. Metab. TEM 2009, 20, 325. 19713122 10.1016/j.tem.2009.03.008 PMC3627124

[122] A. J. Donato , K. A. Magerko , B. R. Lawson , J. R. Durrant , L. A. Lesniewski , D. R. Seals , J. Physiol. 2011, 589, 4545. 21746786 10.1113/jphysiol.2011.211219 PMC3208223

[123] S. Xu , M. Yin , M. Koroleva , M. A. Mastrangelo , W. Zhang , P. Bai , P. J. Little , Z. G. Jin , Aging 2016, 8, 1064. 27249230 10.18632/aging.100975 PMC4931854

[124] A. Roichman , S. Elhanati , M. A. Aon , I. Abramovich , A. Di Francesco , Y. Shahar , M. Y. Avivi , M. Shurgi , A. Rubinstein , Y. Wiesner , A. Shuchami , Z. Petrover , I. Lebenthal‐Loinger , O. Yaron , A. Lyashkov , C. Ubaida‐Mohien , Y. Kanfi , B. Lerrer , P. J. Fernández‐Marcos , M. Serrano , E. Gottlieb , R. de Cabo , H. Y. Cohen , Nat. Commun. 2021, 12, 3208. 34050173 10.1038/s41467-021-23545-7 PMC8163764

[125] A. Cardus , A. K. Uryga , G. Walters , J. D. Erusalimsky , Cardiovasc. Res. 2013, 97, 571. 23201774 10.1093/cvr/cvs352 PMC3567786

[126] C. Urbich , A. Kuehbacher , S. Dimmeler , Cardiovasc. Res. 2008, 79, 581. 18550634 10.1093/cvr/cvn156

[127] C. de Lucia , K. Komici , G. Borghetti , G. D. Femminella , L. Bencivenga , A. Cannavo , G. Corbi , N. Ferrara , S. R. Houser , W. J. Koch , G. Rengo , Front. Med. 2017, 4. 10.3389/fmed.2017.00074 PMC5466994 28660188

[128] S. Du , H. Ling , Z. Guo , Q. Cao , C. Song , Pharmacol. Res. 2021, 165, 105278. 33166733 10.1016/j.phrs.2020.105278

[129] M. Hackl , S. Brunner , K. Fortschegger , C. Schreiner , L. Micutkova , C. Mück , G. T. Laschober , G. Lepperdinger , N. Sampson , P. Berger , D. Herndler‐Brandstetter , M. Wieser , H. Kühnel , A. Strasser , M. Rinnerthaler , M. Breitenbach , M. Mildner , L. Eckhart , E. Tschachler , A. Trost , J. W. Bauer , C. Papak , Z. Trajanoski , M. Scheideler , R. Grillari‐Voglauer , B. Grubeck‐Loebenstein , P. Jansen‐Dürr , J. Grillari , Aging Cell 2010, 9, 291. 20089119 10.1111/j.1474-9726.2010.00549.x PMC2848978

[130] R. Menghini , V. Casagrande , M. Cardellini , E. Martelli , A. Terrinoni , F. Amati , M. Vasa‐Nicotera , A. Ippoliti , G. Novelli , G. Melino , R. Lauro , M. Federici , Circulation 2009, 120, 1524. 19786632 10.1161/CIRCULATIONAHA.109.864629

[131] G. Mandraffino , E. Imbalzano , M. A. Sardo , A. D'Ascola , F. Mamone , A. Lo Gullo , A. Alibrandi , S. Loddo , E. Mormina , A. David , A. Saitta , J. Hum. Hypertens. 2014, 28, 543. 24553637 10.1038/jhh.2014.7

[132] I. Badi , I. Burba , C. Ruggeri , F. Zeni , M. Bertolotti , A. Scopece , G. Pompilio , A. Raucci , J. Gerontol. A Biol. Sci. Med. Sci. 2015, 70, 1304. 25352462 10.1093/gerona/glu180

[133] C. Rippe , M. Blimline , K. A. Magerko , B. R. Lawson , T. J. LaRocca , A. J. Donato , D. R. Seals , Exp. Gerontol. 2012, 47, 45. 22037549 10.1016/j.exger.2011.10.004 PMC3245334

[134] H. Zhang , H. Yang , C. Zhang , Y. Jing , C. Wang , C. Liu , R. Zhang , J. Wang , J. Zhang , K. Zen , C. Zhang , D. Li , J. Gerontol. – Ser. Biol. Sci. Med. Sci. 2015, 70, 102. 10.1093/gerona/glu145 25165030

[135] B. Poeggeler , S. K. Singh , K. Sambamurti , M. A. Pappolla , Int. J. Mol. Sci. 2023, 24, 14533. 37108272 10.3390/ijms24087112 PMC10138358

[136] R. C. María , L. Sánchez de Miguel , M. García‐Durán , F. González‐Fernández , I. Millás , M. Montón , J. Rodrigo , L. Rico , P. Fernández , T. de Frutos , J. A. Rodríguez‐Feo , J. Guerra , C. Caramelo , S. Casado , A. López‐Farré , Circ. Res. 1998, 83, 279. 9710120 10.1161/01.res.83.3.279

[137] A. M. Pourbagher‐Shahri , T. Farkhondeh , M. Talebi , D. M. Kopustinskiene , S. Samarghandian , J. Bernatoniene , Molecules 2021, 26, 4533. 34361685 10.3390/molecules26154533 PMC8348219

[138] D. M. Dudzinski , T. Michel , Cardiovasc. Res. 2007, 75, 247. 17466957 10.1016/j.cardiores.2007.03.023 PMC2682334

[139] A. Janaszak‐Jasiecka , A. Płoska , J. M. Wierońska , L. W. Dobrucki , L. Kalinowski , Cell. Mol. Biol. Lett. 2023, 28, 21. 36890458 10.1186/s11658-023-00423-2 PMC9996905

[140] A. Aicher , C. Heeschen , C. Mildner‐Rihm , C. Urbich , C. Ihling , K. Technau‐Ihling , A. M. Zeiher , S. Dimmeler , Nat. Med. 2003, 9, 1370. 14556003 10.1038/nm948

[141] A. L. Sverdlov , D. T. M. Ngo , W. P. A. Chan , Y. Y. Chirkov , J. D. Horowitz , J. Am. Heart Assoc. 2014, 3, 000973. 10.1161/JAHA.114.000973 PMC4310385 25134680

[142] C. Izzo , P. Vitillo , P. Di Pietro , V. Visco , A. Strianese , N. Virtuoso , M. Ciccarelli , G. Galasso , A. Carrizzo , C. Vecchione , Life 2021, 11, 60. 33467601 10.3390/life11010060 PMC7829951

[143] H. Qian , N. Luo , Y. Chi , J. Aging. Res. 2012, 2012, 121390. 22500225 10.1155/2012/121390 PMC3303603

[144] W. T. Nicholson , B. Vaa , C. Hesse , J. H. Eisenach , M. J. Joyner , Hypertension 2009, 53, 973. 19414649 10.1161/HYPERTENSIONAHA.108.121483 PMC2756097

[145] E. H. C. Tang , P. M. Vanhoutte , Physiol. Genomics. 2008, 32, 409. 18056786 10.1152/physiolgenomics.00136.2007

[146] D. Tian , J. Meng , L. Li , H. Xue , Q. Geng , Y. Miao , M. Xu , R. Wang , X. Zhang , Y. Wu , Acta Biochim. Biophys. Sin. 2023, 55, 1358. 37587757 10.3724/abbs.2023156 PMC10520484

[147] E. Piragine , M. A. Malanima , E. Lucenteforte , A. Martelli , V. Calderone , Biomolecules 2023, 13, 1023. 37509058 10.3390/biom13071023 PMC10376967

[148] Y. Miao , S. Zhang , Z. Liang , Y. Wang , D. Tian , S. Jin , Q.i Guo , H. Xue , X.u Teng , L. Xiao , Y. Wu , Nitric Oxide 2023, 140–141, 77. 10.1016/j.niox.2023.10.002 37875241

[149] X. Qin , F. Lu , J. Wan , X.u Teng , S. Jin , L. Xiao , H. Xue , Q.i Guo , D. Tian , Y. Wu , J. Mol. Cell. Cardiol. 2025, 203, 10. 40209983 10.1016/j.yjmcc.2025.04.005

[150] A. Das , G. X. Huang , M. S. Bonkowski , A. Longchamp , C. Li , M. B. Schultz , L. J. Kim , B. Osborne , S. Joshi , Y. Lu , J. H. Treviño‑Villarreal , M. J. Kang , T. T. Hung , B. Lee , E. O. Williams , M. Igarashi , J. R. Mitchell , L. E. Wu , N. Turner , Z. Arany , L. Guarente , D. A. Sinclair , Vascular Aging. Cell. 2018, 173, 74.e20. 10.1016/j.cell.2018.02.008 PMC5884172 29570999

[151] W. B. Campbell , I. Fleming , Pflugers Arch. 2010, 459, 881. 20224870 10.1007/s00424-010-0804-6 PMC3373596

[152] S. Jiang , S. Han , D. W. Wang , Front. Pharmacol. 2024, 15, 1358256. 38628644 10.3389/fphar.2024.1358256 PMC11019020

[153] J. Wils , Z. Djerada , F. Roca , T. Duflot , M. Iacob , I. Remy‐Jouet , R. Joannides , J. Bellien , Atherosclerosis 2018, 275, 239. 29980050 10.1016/j.atherosclerosis.2018.06.865

[154] C. M. Davis , W. H. Zhang , T. M. Bah , N. E. Roese , E. M. Allen , P. Leung , S. J. Boutros , T. Marzulla , E. Patel , X. Nie , F. N. Alkayed , J. H. Huang , M. A. Jensen , J. Raber , M. M. Pike , N. J. Alkayed , Npj Aging 2022, 8, 9. 35927273 10.1038/s41514-022-00090-1 PMC9256583

[155] M. E. Widlansky , D. D. Gutterman , Antioxid. Redox Signal 2011, 15, 1517. 21194353 10.1089/ars.2010.3642 PMC3151425

[156] M. J. Socha , S. S. Segal , J. Appl. Physiol. 2018, 125, 1851. 30412030 10.1152/japplphysiol.00113.2018 PMC6737458

[157] H. J. Hwang , N. Kim , A. B. Herman , M. Gorospe , J. S. Lee , Int. J. Mol. Sci. 2022, 23, 10135. 36077539 10.3390/ijms231710135 PMC9456027

[158] Y. Han , S. Y. Kim , Exp. Mol. Med. 2023, 55, 1. 36599934 10.1038/s12276-022-00906-w PMC9898542

[159] C. M. Warboys , A. de Luca , N. Amini , L.e Luong , H. Duckles , S. Hsiao , A. White , S. Biswas , R. Khamis , C. K. Chong , W.‐M. Cheung , S. J. Sherwin , M. R. Bennett , J. Gil , J. C. Mason , D. O. Haskard , P. C. Evans , Arterioscler Thromb. Vasc. Biol. 2014, 34, 985. 24651677 10.1161/ATVBAHA.114.303415

[160] L. Toma , G. M. Sanda , C. S. Stancu , L. S. Niculescu , M. Raileanu , A. V. Sima , Biomolecules 2023, 13, 626. 37189375 10.3390/biom13040626 PMC10135653

[161] M. A. Hussein , V. R. Young , E. Murray , N. S. Scrimshaw , J. Nutr. 1971, 101, 61. 5540526 10.1093/jn/101.1.61

[162] Y. Higashi , Antioxidants 2022, 11, 1958. 36290681 10.3390/antiox11101958 PMC9598825

[163] Y. Sun , X. Wang , T. Liu , X. Zhu , X. Pan , Cell Biosci. 2022, 12, 74. 35642067 10.1186/s13578-022-00815-5 PMC9153125

[164] G. Jia , A. R. Aroor , C. Jia , J. R. Sowers , Biochim. Biophys. Acta BBA – Mol. Basis Dis. 2019, 1865, 1802. 10.1016/j.bbadis.2018.08.008 31109450

[165] F. Rodier , J. Campisi , J. Cell Biol. 2011, 192, 547. 21321098 10.1083/jcb.201009094 PMC3044123

[166] T. Tchkonia , Y. Zhu , J. van Deursen , J. Campisi , J. L. Kirkland , J. Clin. Invest. 2013, 123, 966. 23454759 10.1172/JCI64098 PMC3582125

[167] Z. Ungvari , S. Tarantini , Á. Nyúl‐Tóth , T. Kiss , A. Yabluchanskiy , T. Csipo , P. Balasubramanian , A. Lipecz , Z. Benyo , A. Csiszar , GeroScience 2019, 41, 727. 31655958 10.1007/s11357-019-00107-w PMC6925097

[168] T. Hayashi , H. Matsui‐Hirai , A. Miyazaki‐Akita , A. Fukatsu , J. Funami , Q.‐F. Ding , S. Kamalanathan , Y. Hattori , L. J. Ignarro , A. Iguchi , Proc. Natl. Acad. Sci. U S A. 2006, 103, 17018. 17075048 10.1073/pnas.0607873103 PMC1629003

[169] S. Aldosari , M. Awad , E. O. Harrington , F. W. Sellke , M. R. Abid , Antioxidants 2018, 7, 14. 29337890 10.3390/antiox7010014 PMC5789324

[170] K. Foote , M. Rienks , L. Schmidt , K. Theofilatos , Y. Matiss Ozols , A. Eckersley , A. Shah , N. Figg , A. Finigan , K. O’Shaughnessy , I. Wilkinson , M. Mayr , M. Bennett , Cardiovasc. Res. 2025, 121, 614. 38717632 10.1093/cvr/cvae091 PMC12054627

[171] M. El Assar , J. Angulo , L. Rodríguez‐Mañas , Free Radic. Biol. Med. 2013, 65, 380. 23851032 10.1016/j.freeradbiomed.2013.07.003

[172] Z. Ungvari , S. Tarantini , T. Kiss , J. D. Wren , C. B. Giles , C. T. Griffin , W. L. Murfee , P. Pacher , A. Csiszar , Nat. Rev. Cardiol. 2018, 15, 555. 29795441 10.1038/s41569-018-0030-z PMC6612360

[173] S. B. A. Cau , F. S. Carneiro , R. C. Tostes , Front. Physiol. 2012, 3, 218. 22737132 10.3389/fphys.2012.00218 PMC3382417

[174] T. Hayashi , K. Yano , H. Matsui‐Hirai , H. Yokoo , Y. Hattori , A. Iguchi , Pharmacol. Ther. 2008, 120, 333. 18930078 10.1016/j.pharmthera.2008.09.002

[175] A. Csiszar , M. Wang , E. G. Lakatta , Z. Ungvari , J. Appl. Physiol. 2008, 105, 1333. 18599677 10.1152/japplphysiol.90470.2008 PMC2576023

[176] L. Ferrucci , E. Fabbri , Nat. Rev. Cardiol. 2018, 15, 505. 30065258 10.1038/s41569-018-0064-2 PMC6146930

[177] B. P. Yu , H. Y. Chung , Mech. Ageing Dev. 2006, 127, 436. 16497363 10.1016/j.mad.2006.01.023

[178] I. Mattagajasingh , C.‐S. Kim , A. Naqvi , T. Yamamori , T. A. Hoffman , S.‐B. Jung , J. DeRicco , K. Kasuno , K. Irani , Proc. Natl. Acad. Sci. U S A. 2007, 104, 14855. 17785417 10.1073/pnas.0704329104 PMC1976244

[179] A. Salminen , J. Huuskonen , J. Ojala , A. Kauppinen , K. Kaarniranta , T. Suuronen , Ageing Res. Rev. 2008, 7, 83. 17964225 10.1016/j.arr.2007.09.002

[180] Y. Li , D. Kracun , C. M. Dustin , M. El Massry , S. Yuan , C. J. Goossen , E. R. DeVallance , S. Sahoo , C. St. Hilaire , A. U. Gurkar , T. Finkel , A. C. Straub , E. Cifuentes‐Pagano , P. J. Pagano , Proc. Natl. Acad. Sci. 2021, 118, 2015666118. 10.1073/pnas.2015666118 PMC8594573 34654740

[181] G. Pacinella , A. M. Ciaccio , A. Tuttolomondo , Int. J. Mol. Sci. 2022, 23, 15722. 36555364 10.3390/ijms232415722 PMC9779461

[182] H. Yousef , C. J. Czupalla , D. Lee , M. B. Chen , A. N. Burke , K. A. Zera , J. Zandstra , E. Berber , B. Lehallier , V. Mathur , R. V. Nair , L. N. Bonanno , A. C. Yang , T. Peterson , H. Hadeiba , T. Merkel , J. Körbelin , M. Schwaninger , M. S. Buckwalter , S. R. Quake , E. C. Butcher , T. Wyss‐Coray , Nat. Med. 2019, 25, 988. 31086348 10.1038/s41591-019-0440-4 PMC6642642

[183] W. A. Muller , Vet. Pathol. 2013, 50, 7. 23345459 10.1177/0300985812469883 PMC3628536

[184] Z. Springo , S. Tarantini , P. Toth , Z. Tucsek , A. Koller , W. E. Sonntag , A. Csiszar , Z. Ungvari , J. Gerontol. A Biol. Sci. Med. Sci. 2015, 70, 1355. 25631392 10.1093/gerona/glu244 PMC4612385

[185] Y. J. Li , X. Jin , D. Li , J. Lu , X. N. Zhang , S. J. Yang , Y. X. Zhao , M. Wu , Biomed. Pharmacother. 2022, 156, 113954. 36411638 10.1016/j.biopha.2022.113954

[186] H. Li , L. Shen , P. Hu , R. Huang , Y. Cao , J. Deng , W. Yuan , D. Liu , J. Yang , H. Gu , Y. Bai , Biochim. Biophys. Acta BBA – Mol. Basis Dis. 2017, 1863, 2266. 10.1016/j.bbadis.2017.05.022 28559044

[187] L. Wang , Q. Zhang , K. Yuan , J. Yuan , Dis. Markers 2021, 2021, 7157109. 34795807 10.1155/2021/7157109 PMC8595034

[188] H. Yu , K.e Liao , Y.u Hu , D. Lv , M. Luo , Q. Liu , L. Huang , S. Luo , Aging Dis. 2022, 13, 1901. 36465181 10.14336/AD.2022.0316 PMC9662267

[189] Y. J. Liu , R. L. McIntyre , G. E. Janssens , R. H. Houtkooper , Mech. Ageing Dev. 2020, 186, 111212. 32017944 10.1016/j.mad.2020.111212

[190] H. H. Wang , Y. J. Wu , Y. M. Tseng , C. H. Su , C. L. Hsieh , H. I. Yeh , Angiogenesis 2019, 22, 569. 31482366 10.1007/s10456-019-09680-2

[191] M. Miyao , S. Cicalese , T. Kawai , H. A. Cooper , M. J. Boyer , K. J. Elliott , S. J. Forrester , R. Kuroda , V. Rizzo , T. Hashimoto , R. Scalia , S. Eguchi , Int. J. Mol. Sci. 2020, 21, 3112. 32354103 10.3390/ijms21093112 PMC7247685

[192] J. M. Edelberg , M. J. Reed , Front. Biosci. J. Virtual Libr. 2003, 8, s1199. 10.2741/1166 12957863

[193] A. Rivard , L. Berthou‐Soulie , N. Principe , M. Kearney , C. Curry , D. Branellec , G. L. Semenza , J. M. Isner , J Biol. Chem. 2000, 275, 29643. 10882714 10.1074/jbc.M001029200

[194] O. I. Ndubuizu , J. C. Chavez , J. C. LaManna , Am. J. Physiol. Regul. Integr. Comp. Physiol. 2009, 297, R158. 19420289 10.1152/ajpregu.90829.2008 PMC2711700

[195] M. F. Oellerich , M. Potente , D. Sinclair , B. North , Circ. Res. 2012, 110, 1238.22539757

[196] B. Lehallier , D. Gate , N. Schaum , T. Nanasi , S. E. Lee , H. Yousef , P. Moran Losada , D. Berdnik , A. Keller , J. Verghese , S. Sathyan , C. Franceschi , S. Milman , N. Barzilai , T. Wyss‐Coray , Nat. Med. 2019, 25, 1843. 31806903 10.1038/s41591-019-0673-2 PMC7062043

[197] A. Papapetropoulos , G. García‐Cardeña , J. A. Madri , W. C. Sessa , J. Clin. Invest. 1997, 100, 3131. 9399960 10.1172/JCI119868 PMC508526

[198] H. J. Yoon , S. W. Cho , B. W. Ahn , S. Y. Yang , Mech. Ageing Dev. 2010, 131, 119. 20064546 10.1016/j.mad.2009.12.010

[199] D. Soni , S. C. Regmi , D.‐M. Wang , A. DebRoy , Y.‐Y. Zhao , S. M. Vogel , A. B. Malik , C. Tiruppathi , Am. J. Physiol.‐Lung Cell Mol Physiol 2017, 312, L1003. 28385807 10.1152/ajplung.00008.2017 PMC5495943

[200] M. D. Sweeney , A. P. Sagare , B. V. Zlokovic , Nat. Rev. Neurol. 2018, 14, 133. 29377008 10.1038/nrneurol.2017.188 PMC5829048

[201] I. C. M. Verheggen , J. J. A. de Jong , M. P. J. van Boxtel , E.d H. B. M. Gronenschild , W. M. Palm , A. A. Postma , J. F. A. Jansen , F. R. J. Verhey , W. H. Backes , GeroScience 2020, 42, 1183. 32601792 10.1007/s11357-020-00211-2 PMC7394987

[202] D. Mehta , A. B. Malik , Physiol. Rev. 2006, 86, 279. 16371600 10.1152/physrev.00012.2005

[203] C. N. Duong , D. Vestweber , Front. Physiol. 2020, 11, 519. 32670077 10.3389/fphys.2020.00519 PMC7326147

[204] F. Chang , S. Flavahan , N. A. Flavahan , J Physiol 2017, 595, 5143. 28561330 10.1113/JP274189 PMC5538197

[205] S. I. Graves , D. J. Baker , Basic Clin. Pharmacol. Toxicol. 2020, 127, 102. 32162446 10.1111/bcpt.13403 PMC7384943

[206] E. Fahey , S. L. Doyle , Front Immunol 2019, 10, 1426. 31293586 10.3389/fimmu.2019.01426 PMC6603210

[207] J. P. Coppé , K. Kauser , J. Campisi , C. M. Beauséjour , J. Biol. Chem. 2006, 281, 29568. 16880208 10.1074/jbc.M603307200

[208] C. Ivaldo , M. Passalacqua , A. L. Furfaro , d'C. Abramo , S. Ruiz , P. K. Chatterjee , et al., Sci. Rep. 2023, 13, 597. 36631513 10.1038/s41598-022-27308-2 PMC9834263

[209] D. Predescu , S. Predescu , J. Shimizu , K. Miyawaki‐Shimizu , A. B. Malik , Am. J. Physiol. Lung Cell Mol. Physiol 2005, 289, L371. 16093363 10.1152/ajplung.00175.2004

[210] D. Miyazaki , M. Kandori‐Inoue , Y. Shimizu , F. Ohtani , I. Chono , Y. Inoue , S. Yamagami , Int. J. Mol. Sci. 2021, 22, 5833. 34072468 10.3390/ijms22115833 PMC8199122

[211] Y. Weng , N. Chen , R. Zhang , J. He , X. Ding , G. Cheng , Q. Bi , Y.‐M. Lu , X. Z. Shen , S. Wan , P. Shi , Brain Behav. Immun. 2024, 115, 705. 37992789 10.1016/j.bbi.2023.11.023

[212] H. Valadi , K. Ekström , A. Bossios , M. Sjöstrand , J. J. Lee , J. O. Lötvall , Nat. Cell Biol. 2007, 9, 654. 17486113 10.1038/ncb1596

[213] R. Feng , M. Ullah , K. Chen , Q. Ali , Y. Lin , Z. Sun , J Extracell Vesicles 2020, 9, 1783869. 32939234 10.1080/20013078.2020.1783869 PMC7480600

[214] A. Turchinovich , T. R. Samatov , A. G. Tonevitsky , B. Burwinkel , Front Genet. 2013. 10.3389/fgene.2013.00119 PMC3695387 23825476

[215] Y. Yang , Y. Cai , Y. Zhang , J. Liu , Z. Xu , J Mol Neurosci. MN 2018, 65, 74. 29705934 10.1007/s12031-018-1071-9

[216] M. Ye , Q. Ni , H. Qi , X. Qian , J. Chen , X. Guo , M. Li , Y. Zhao , G. Xue , H. Deng , L. Zhang , Int. J. Biol. Sci. 2019, 15, 158. 30662356 10.7150/ijbs.28392 PMC6329927

[217] M. R. Hoenig , C. Bianchi , A. Rosenzweig , F. W. Sellke , Curr. Mol. Med. 2008, 8, 754. 19075673 10.2174/156652408786733685

[218] P. F. Wong , K. L. Tong , J. Jamal , E. S. Khor , S. L. Lai , M. R. Mustafa , EXCLI J. 2019, 18, 764. 31611757 10.17179/excli2019-1505 PMC6785768

[219] S. Weilner , E. Schraml , M. Wieser , P. Messner , K. Schneider , K. Wassermann , L. Micutkova , K. Fortschegger , A. B. Maier , R. Westendorp , H. Resch , S. Wolbank , H. Redl , P. Jansen‐Dürr , P. Pietschmann , R. Grillari‐Voglauer , J. Grillari , Aging Cell 2016, 15, 744. 27146333 10.1111/acel.12484 PMC4933673

[220] J. Li , J. Tan , Q. Song , X. Yang , X. Zhang , H. Qin , G. Huang , X. Su , J. Li , J. Mol. Histol. 2023, 54, 13. 36409439 10.1007/s10735-022-10107-4 PMC9908644

[221] M. Alique , M. P. Ruíz‐Torres , G. Bodega , M. V. Noci , N. Troyano , L. Bohórquez , C. Luna , R. Luque , A. Carmona , J. Carracedo , R. Ramírez , Aging 2017, 9, 778. 28278131 10.18632/aging.101191 PMC5391231

[222] T. M. Sonninen , G. Goldsteins , N. Laham‐Karam , J. Koistinaho , Š. Lehtonen , Cells 2020, 9, 2183. 32998318 10.3390/cells9102183 PMC7601929

[223] M. Gouveia , K. Xia , W. Colón , S. I. Vieira , F. Ribeiro , Ageing Res. Rev. 2017, 40, 1. 28757291 10.1016/j.arr.2017.07.005

[224] H. V. Hwang , Y. Lin , M. N. Rebuffatti , D. T. Tran , L. Lee , A. V. Gomes , C.‐S. Li , A. A. Knowlton , Am J. Physiol.‐Heart. Circ. Physiol. 2019, 316, H421. 30499713 10.1152/ajpheart.00318.2018

[225] J. H. Chin , M. Okazaki , Z. W. Hu , J. W. Miller , B. B. Hoffman , J. Clin. Invest. 1996, 97, 2316. 8636412 10.1172/JCI118674 PMC507312

[226] C. Lopez‐Otin , M. A. Blasco , L. Partridge , M. Serrano , G. Kroemer , Cell 2023, 186, 243. 36599349 10.1016/j.cell.2022.11.001

[227] T. J. LaRocca , G. D. Henson , A. Thorburn , A. L. Sindler , G. L. Pierce , D. R. Seals , J. Physiol. 2012, 590, 3305. 22570377 10.1113/jphysiol.2012.229690 PMC3459044

[228] C. Schrimpf , O. E. Teebken , M. Wilhelmi , J. S. Duffield , J. Vasc. Res. 2014, 51, 247. 25195856 10.1159/000365149 PMC4476411

[229] A. Armulik , G. Genové , M. Mäe , M. H. Nisancioglu , E. Wallgard , C. Niaudet , L. He , J. Norlin , P. Lindblom , K. Strittmatter , B. R. Johansson , C. Betsholtz , Nature 2010, 468, 557. 20944627 10.1038/nature09522

[230] M. R. Hayden , J. Alzheim. Neurodegenerat. Dis. 2023, 9, 062.

[231] A. Tamiato , L. S. Tombor , A. Fischer , M. Muhly‐Reinholz , L. R. Vanicek , B. S. N. Togru , J. Neitz , S. F. Glaser , M. Merten , D. Rodriguez Morales , J. Kwon , S. Klatt , B. Schumacher , S. Günther , W. T. Abplanalp , D. John , I. Fleming , N. Wettschureck , S. Dimmeler , G. Luxán , Circ. Res. 2024, 134, 1240. 38563133 10.1161/CIRCRESAHA.123.324183 PMC11081481

[232] R. Ding , Y. Hase , M. Burke , V. Foster , W. Stevenson , T. Polvikoski , R. N. Kalaria , Acta Neuropathol. Commun. 2021, 9, 130. 34340718 10.1186/s40478-021-01230-6 PMC8330023

[233] A.‐A. Berthiaume , F. Schmid , S. Stamenkovic , V. Coelho‐Santos , C. D. Nielson , B. Weber , M. W. Majesky , A. Y. Shih , Nat. Commun. 2022, 13, 5912. 36207315 10.1038/s41467-022-33464-w PMC9547063

[234] I. Soto , L. C. Graham , H. J. Richter , S. N. Simeone , J. E. Radell , W. Grabowska , W. K. Funkhouser , M. C. Howell , G. R. Howell , PLoS Biol. 2015, 13, 1002279. 10.1371/journal.pbio.1002279 PMC4626092 26512759

[235] P. A. Stewart , M. Magliocco , K. Hayakawa , C. L. Farrell , R. F. Del Maestro , J. Girvin , J. C. E. Kaufmann , H. V. Vinters , J. Gilbert , Microvasc. Res. 1987, 33, 270. 3587079 10.1016/0026-2862(87)90022-7

[236] G. Liu , J. Wang , Z. Wei , C.‐L. Fang , K.e Shen , C. Qian , C. Qi , T. Li , P. Gao , P. C. Wong , H. Lu , X.u Cao , M. Wan , Adv. Sci. 2023, 10, 2206938. 10.1002/advs.202206938 PMC10369301 37102631

[237] H. D. Kim , E. N. Kim , J.i H. Lim , Y. Kim , T. H. Ban , H. Lee , Y. S.u Kim , C. W. Park , B. S. Choi , Aging Cell 2024, 23, 14075. 10.1111/acel.14075 PMC10928568 38155524

[238] Z. Zhao , Y. Zhang , C. Zhang , J. Zhang , X. Luo , Q. Qiu , D. Luo , J. Zhang , Exp. Mol. Med. 2022, 54, 673. 35624154 10.1038/s12276-022-00778-0 PMC9166792

[239] F. Yao , Y. Luo , Y. C. Liu , Y. H. Chen , Y. T. Li , X. Y. Hu , X. Y. You , S. S. Yu , Z. Y. Li , L. Chen , D. S. Tian , M. G. Zheng , L. Cheng , J. H. Jing , Inflamm Regen. 2022, 42, 44. 36163271 10.1186/s41232-022-00223-9 PMC9511779

[240] A. Zullo , F. P. Mancini , R. Schleip , S. Wearing , K. W. Fibrosis , Wound Repair Regen. 2021, 29, 650. 34077595 10.1111/wrr.12943

[241] N. Park , H. Kang , Int. J. Mol. Sci. 2020, 21, 4764. 32635504 10.3390/ijms21134764 PMC7369869

[242] G. Cao , X. Xuan , J. Hu , R. Zhang , H. Jin , H. Dong , Cell Commun. Signal CCS 2022, 20, 180. 36411459 10.1186/s12964-022-00993-2 PMC9677683

[243] A. Y. Kolyada , K. N. Riley , I. M. Herman , Am. J. Physiol.‐Cell. Physiol. 2003, 285, C1116. 14532019 10.1152/ajpcell.00177.2003

[244] L. Feng , D. Que , Z. Li , X. Zhong , J. Yan , J. Wei , X. Zhang , P. Yang , C. Ou , M. Chen , Clin. Sci. 2021, 135, 2483. 10.1042/CS20210259 34643227

[245] A. J. Pedroza , R. Shad , A. R. Dalal , N. Yokoyama , K. Nakamura , W. Hiesinger , M. P. Fischbein , Hypertension 2022, 79, 86. 10.1161/HYPERTENSIONAHA.121.18640 PMC8916978 35124970

[246] S. Tsai , S. T. Hollenbeck , E. J. Ryer , R. Edlin , D. Yamanouchi , R. Kundi , C. Wang , B.o Liu , K. C. Kent , Am. J. Physiol.‐Heart Circ. Physiol. 2009, 297, H540. 19525370 10.1152/ajpheart.91478.2007 PMC2724222

[247] I. Six , H. Okazaki , P. Gross , J. Cagnard , C. Boudot , J. Maizel , T. B. Drueke , Z. A. Massy , PLoS One 2014, 9, 93423. 10.1371/journal.pone.0093423 PMC3973676 24695641

[248] S. Yu , Y. Chen , S. Chen , N. Ye , Y. Li , Y. Sun , Med. Sci. Monit. 2018, 24, 4851. 30004089 10.12659/MSM.908038 PMC6069467

[249] I. Badi , L. Mancinelli , A. Polizzotto , D. Ferri , F. Zeni , I. Burba , G. Milano , F. Brambilla , C. Saccu , M. E. Bianchi , G. Pompilio , M. C. Capogrossi , A. Raucci , Arterioscler Thromb Vasc. Biol. 2018, 38, 2079. 30026277 10.1161/ATVBAHA.118.311298

[250] J. N. Mansbridge , K. Liu , R. E. Pinney , R. Patch , A. Ratcliffe , G. K. Naughton , Diabetes Obes. Metab. 1999, 1, 265. 11225638 10.1046/j.1463-1326.1999.00032.x

[251] V. S. LeBleu , B. Macdonald , R. Kalluri , Exp. Biol. Med. Maywood NJ 2007, 232, 1121. 10.3181/0703-MR-72 17895520

[252] S. H. Lim , C. Kim , A. R. Aref , R. D. Kamm , M. Raghunath , Integr. Biol. 2013, 5, 1474. 10.1039/c3ib40082d 24190477

[253] N. Walji , S. Kheiri , E. W. K. Young , Adv. Biol. 2021, 5, 2101080. 10.1002/adbi.202101080 34655165

[254] A. Hielscher , K. Ellis , C. Qiu , J. Porterfield , S. Gerecht , PLoS One 2016, 11, 0147600. 10.1371/journal.pone.0147600 PMC4728102 26811931

[255] U. Lendahl , L. Muhl , C. Betsholtz , Nat. Commun. 2022, 13, 3409. 35701396 10.1038/s41467-022-30633-9 PMC9192344

[256] A. Orimo , R. A. Weinberg , Cell Cycle 2006, 5, 1597. 16880743 10.4161/cc.5.15.3112

[257] K. Tominaga‐Yamanaka , K. Abdelmohsen , J. L. Martindale , X. Yang , D. D. Taub , M. Gorospe , Aging 2012, 4, 695. 23117626 10.18632/aging.100497 PMC3517940

[258] J. Wolf , B. Weinberger , C. R. Arnold , A. B. Maier , R. G. J. Westendorp , Exp. Gerontol. 2012, 47, 749. 22790019 10.1016/j.exger.2012.07.001 PMC3427851

[259] Y. Ogata , T. Yamada , S. Hasegawa , K. Sugiura , H. Akamatsu , Exp. Dermatol. 2023, 32, 1159. 37087745 10.1111/exd.14818

[260] J. Zhang , H. Yu , M. Q. Man , L. Hu , Aging Cell 2024, 23, 14054. 10.1111/acel.14054 PMC10861215 38040661

[261] Y. H. Kim , Y.‐K. Lee , S. S. Park , S. H. Park , S. Y. Eom , Y.‐S. Lee , W. J. Lee , J. Jang , D. Seo , H. Y. Kang , J. C. Kim , S. B. Lim , G. Yoon , H. S. Kim , J.‐H. Kim , T. J. Park , Nat. Commun. 2023, 14, 7619. 37993434 10.1038/s41467-023-43491-w PMC10665435

[262] W. Lupa , F. Kalfalah , K. Safferling , P. Boukamp , G. Poschmann , E. Volpi , C. Götz‑Rösch , F. Bernerd , L. Haag , U. Huebenthal , E. Fritsche , F. Boege , N. Grabe , J. Tigges , K. Stühler , J. Krutmann , J. Invest. Dermatol. 2015, 135, 1954. 25815425 10.1038/jid.2015.120

[263] K. T. Weber , Y. Sun , S. C. Tyagi , J. P. M. Cleutjens , J. Mol. Cell. Cardiol. 1994, 26, 279. 8028011 10.1006/jmcc.1994.1036

[264] P. K. Aujla , Z. Kassiri , Cell Signal 2021, 78, 109869. 33278559 10.1016/j.cellsig.2020.109869

[265] S. Bersini , R. Arrojo e Drigo , L. Huang , M. N. Shokhirev , M. W. Hetzer , Adv. Biosyst. 2020, 4, 2000044. 10.1002/adbi.202000044 32402127

[266] S. Wang , T. Huo , M. Lu , Y. Zhao , J. Zhang , W. He , H. Chen , Cells 2025, 14, 499. 40214453 10.3390/cells14070499 PMC11987807

[267] Z. Liu , Q. Liang , Y. Ren , C. Guo , X. Ge , L. Wang , Q. Cheng , P. Luo , Y. Zhang , X. Han , Signal Transduct. Target Ther. 2023, 8, 200. 37179335 10.1038/s41392-023-01451-2 PMC10182360

[268] H. Winkels , C. Weber , E. Lutgens , N. Gerdes , Curr. Opin. Lipidol. 2014, 25, 408. 25186203 10.1097/MOL.0000000000000120

[269] W. Chen , W. Xiao , X. Liu , P. Yuan , S. Zhang , Y. Wang , W. Wu , Bioact. Mater. 2021, 11, 283. 34977432 10.1016/j.bioactmat.2021.09.027 PMC8668428

[270] H. Greigert , C. Genet , A. Ramon , B. Bonnotte , M. Samson , J. Clin. Med. 2022, 11, 2905. 35629030 10.3390/jcm11102905 PMC9143803

[271] Y. P. Fang , Y. Zhao , J. Y. Huang , X. Yang , Y. Liu , X. L. Zhang , Front. Endocrinol. 2024, 15. 10.3389/fendo.2024.1330942 PMC10839002 38318291

[272] X. Song , Y. Song , Q. Ma , K. Fang , X. Chang , J. Inflamm. Res. 2023, 16, 3019. 37489150 10.2147/JIR.S413358 PMC10363393

[273] H. M. Abdolmaleky , J. R. Zhou , Nutrients 2023, 15, 3456.37571393

[274] M. Djurišić , Biosci. Rep. 2024, 44, BSR20222267. 38299364 10.1042/BSR20222267 PMC10866841

[275] N. E. Propson , E. R. Roy , A. Litvinchuk , J. Köhl , H. Zheng , J. Clin. Invest. 2021, 131, e140966. 10.1172/JCI140966 PMC7773352 32990682

[276] M. Wang , X. Yin , S. Zhang , C. Mao , N. Cao , X. Yang , J. Bian , W. Hao , Q. Fan , H. Liu , Aging Dis. 2019, 10, 1012. 31595199 10.14336/AD.2018.0919 PMC6764731

[277] A. Kallai , A. Ungvari , D. Csaban , Z. Orfi , A. Lehoczki , J. Harasztdombi , A. Yabluchanskiy , Z. Benyó , Á. Szappanos , S. Tarantini , F. Sorond , P. Sótonyi , H. Andrikovics , Z. Ungvari , GeroScience. 2025, 47, 2739. 40214958 10.1007/s11357-025-01654-1 PMC12181173

[278] N. Schaum , B. Lehallier , O. Hahn , R. Pálovics , S. Hosseinzadeh , S. E. Lee , R. Sit , D. P. Lee , P. M. Losada , M. E. Zardeneta , T. Fehlmann , J. T. Webber , A. McGeever , K. Calcuttawala , H. Zhang , D. Berdnik , V. Mathur , W. Tan , A. Zee , M. Tan , N. Almanzar , J. Antony , A. S. Baghel , I. Bakerman , I. Bansal , B. A. Barres , P. A. Beachy , D. Berdnik , B. Bilen , D. Brownfield , et al., Nat 2020, 583, 596.

[279] S. Bersini , C. Arrigoni , G. Talò , C. Candrian , M. Moretti , iScience 2024, 27, 109199. 38433912 10.1016/j.isci.2024.109199 PMC10904982

[280] S. Holtze , E. Gorshkova , S. Braude , A. Cellerino , P. Dammann , T. B. Hildebrandt , A. Hoeflich , S. Hoffmann , P. Koch , E. Terzibasi Tozzini , M. Skulachev , V. P. Skulachev , A. Sahm , Front. Mol. Biosci. 2021, 8, 660959. 34079817 10.3389/fmolb.2021.660959 PMC8166319

[281] D. A. Sinclair , Mech. Ageing Dev. 2005, 126, 987. 15893363 10.1016/j.mad.2005.03.019

[282] J. A. Baur , K. J. Pearson , N. L. Price , H. A. Jamieson , C. Lerin , A. Kalra , V. V. Prabhu , J. S. Allard , G. Lopez‐Lluch , K. Lewis , P. J. Pistell , S. Poosala , K. G. Becker , O. Boss , D. Gwinn , M. Wang , S. Ramaswamy , K. W. Fishbein , R. G. Spencer , E. G. Lakatta , D. Le Couteur , R. J. Shaw , P. Navas , P. Puigserver , D. K. Ingram , R. de Cabo , D. A. Sinclair , Nature 2006, 444, 337. 17086191 10.1038/nature05354 PMC4990206

[283] S. J. Mitchell , M. Scheibye‐Knudsen , D. L. Longo , R. de Cabo , Annu. Rev. Anim. Biosci. 2015, 3, 283. 25689319 10.1146/annurev-animal-022114-110829

[284] G. Vitale , G. Pellegrino , M. Vollery , L. J. Hofland , Front. Endocrinol. 2019, 10, 27. 10.3389/fendo.2019.00027 PMC6367275 30774624

[285] R. K. Junnila , E. O. List , D. E. Berryman , J. W. Murrey , J. J. Kopchick , Nat. Rev. Endocrinol. 2013, 9, 366. 23591370 10.1038/nrendo.2013.67 PMC4074016

[286] R. Pálovics , A. Keller , N. Schaum , W. Tan , T. Fehlmann , M. Borja , F. Kern , L. Bonanno , K. Calcuttawala , J. Webber , A. McGeever , N. Almanzar , J. Antony , A. S. Baghel , I. Bakerman , I. Bansal , B. A. Barres , P. A. Beachy , D. Berdnik , B. Bilen , D. Brownfield , C. Cain , C. K. F. Chan , M. B. Chen , M. F. Clarke , S. D. Conley , A. Demers , K. Demir , A. de Morree , T. Divita , et al., Nature 2022, 603, 309.35236985

[287] A. Eggel , T. Wyss‐Coray , Swiss. Med. Wkly 2014, 144, w13914. 24496774 10.4414/smw.2014.13914 PMC4082987

[288] I. M. Conboy , M. J. Conboy , A. J. Wagers , E. R. Girma , I. L. Weissman , T. A. Rando , Nature 2005, 433, 760. 15716955 10.1038/nature03260

[289] S. A. Villeda , J. Luo , K. I. Mosher , B. Zou , M. Britschgi , G. Bieri , T. M. Stan , N. Fainberg , Z. Ding , A. Eggel , K. M. Lucin , E. Czirr , J.‐S. Park , S. Couillard‐Després , L. Aigner , G. Li , E. R. Peskind , J. A. Kaye , J. F. Quinn , D. R. Galasko , X. S. Xie , T. A. Rando , T. Wyss‐Coray , Nature 2011, 477, 90. 21886162 10.1038/nature10357 PMC3170097

[290] J. S. Kang , Y. R. Yang , Aging 2020, 12, 23394. 33197235 10.18632/aging.103933 PMC7746393

[291] L. Katsimpardi , N. K. Litterman , P. A. Schein , C. M. Miller , F. S. Loffredo , G. R. Wojtkiewicz , J. W. Chen , R. T. Lee , A. J. Wagers , L. L. Rubin , Science 2014, 344, 630. 24797482 10.1126/science.1251141 PMC4123747

[292] M. Ximerakis , K. M. Holton , R. M. Giadone , C. Ozek , M. Saxena , S. Santiago , X. Adiconis , D. Dionne , L. Nguyen , K. M. Shah , J. M. Goldstein , C. Gasperini , I. A. Gampierakis , S. L. Lipnick , S. K. Simmons , S. M. Buchanan , A. J. Wagers , A. Regev , J. Z. Levin , L. L. Rubin , Nat. Aging 2023, 3, 327. 37118429 10.1038/s43587-023-00373-6 PMC10154248

[293] R. Gulej , Á. Nyúl‐Tóth , B. Csik , R. Patai , B. Petersen , S. Negri , S. S. Chandragiri , S. Shanmugarama , P. Mukli , A. Yabluchanskiy , S. Conley , D. Huffman , S. Tarantini , A. Csiszar , Z. Ungvari , GeroScience. 2024, 46, 4415. 38727872 10.1007/s11357-024-01154-8 PMC11336025

[294] R. Gulej , Á. Nyúl‐Tóth , B. Csik , B. Petersen , J. Faakye , S. Negri , S. S. Chandragiri , P. Mukli , A. Yabluchanskiy , S. Conley , D. M. Huffman , A. Csiszar , S. Tarantini , Z. Ungvari , GeroScience. 2024, 46, 327. 38123890 10.1007/s11357-023-01039-2 PMC10828280

[295] A. R. Folgueras , S. Freitas‐Rodríguez , G. Velasco , C. López‐Otín , Circ. Res. 2018, 123, 905. 30355076 10.1161/CIRCRESAHA.118.312204

[296] G. Weeda , I. Donker , J. de Wit , H. Morreau , R. Janssens , C. J. Vissers , A. Nigg , H. van Steeg , D. Bootsma , J. H. J. Hoeijmakers , Curr. Biol. 1997, 7, 427. 9197240 10.1016/s0960-9822(06)00190-4

[297] J. van der Linden , S. J. M. Stefens , J. M. Heredia‐Genestar , Y. Ridwan , R. M. C. Brandt , N. van Vliet , I. de Beer , B. S. van Thiel , H. Steen , C. Cheng , A. J. M. Roks , A. H. J. Danser , J. Essers , I. van der Pluijm , Aging Cell 2024, 23, 14126. 10.1111/acel.14126 PMC11113264 38451018

[298] P. K. Bautista‐Niño , E. Portilla‐Fernandez , E. Rubio‐Beltrán , J. J. van der Linden , R. de Vries , R. van Veghel , M. de Boer , M. Durik , Y. Ridwan , R. Brandt , J. Essers , R. I. Menzies , R. Thomas , A. de Bruin , D. J. Duncker , H. M. M. van Beusekom , M. Ghanbari , J. H. J. Hoeijmakers , R. Sedlacek , R. M. Touyz , A. C. Montezano , I. van der Pluijm , A. H. J. Danser , K. A. Haanes , A. J. M. Roks , Clin. Sci. 2020, 134, 727. 10.1042/CS20190124 32202295

[299] C. E. Hansen , D. Vacondio , L. van der Molen , A. A. Jüttner , W. K. Fung , M. Karsten , B. van het Hof , R. D. Fontijn , G. Kooij , M. E. Witte , A. J. M. Roks , H. E. de Vries , I. Mulder , N. M. de Wit , Cell Death Dis. 2025, 16, 1. 39753531 10.1038/s41419-024-07306-0 PMC11698980

[300] L. Rolas , M. Stein , A. Barkaway , N. Reglero‐Real , E. Sciacca , M. Yaseen , H. Wang , L. Vazquez‐Martinez , M. Golding , I. A. Blacksell , M. J. Giblin , E. Jaworska , C. L. Bishop , M.‐B. Voisin , C. Gaston‐Massuet , L. Fossati‐Jimack , C. Pitzalis , D. Cooper , T. D. Nightingale , C. Lopez‐Otin , M. J. Lewis , S. Nourshargh , EMBO Rep. 2024, 25, 3842. 38918502 10.1038/s44319-024-00182-x PMC11387759

[301] V. Vanhooren , C. Libert , Ageing Res. Rev. 2013, 12, 8. 22543101 10.1016/j.arr.2012.03.010

[302] J. M. Zahn , S. Poosala , A. B. Owen , D. K. Ingram , A. Lustig , A. Carter , A. T. Weeraratna , D. D. Taub , M. Gorospe , K. Mazan‐Mamczarz , E. G. Lakatta , K. R. Boheler , X. Xu , M. P. Mattson , G. Falco , M. S. H. Ko , D. Schlessinger , J. Firman , S. K. Kummerfeld , W. H. Wood , A. B. Zonderman , S. K. Kim , K. G. Becker , PLoS Genet. 2007, 3, 201. 10.1371/journal.pgen.0030201 PMC2098796 18081424

[303] J. Yang , T. Huang , F. Petralia , Q. Long , B. Zhang , C. Argmann , Y. Zhao , C. V. Mobbs , E. E. Schadt , J. Zhu , Z. Tu , K. G. Ardlie , D. S. Deluca , A. V. Segrè , T. J. Sullivan , T. R. Young , E. T. Gelfand , C. A. Trowbridge , J. B. Maller , T. Tukiainen , M. Lek , L. D. Ward , P. Kheradpour , B. Iriarte , Y. Meng , C. D. Palmer , W. Winckler , J. Hirschhorn , M. Kellis , D. G. MacArthur , et al., Sci. Rep. 2015, 5, 15145.26477495

[304] E. M. Mercken , M. Capri , B. A. Carboneau , M. Conte , J. Heidler , A. Santoro , A. Martin‐Montalvo , M. Gonzalez‐Freire , H. Khraiwesh , J. A. González‐Reyes , R. Moaddel , Y. Zhang , K. G. Becker , J. M. Villalba , J. A. Mattison , I. Wittig , C. Franceschi , R. de Cabo , Npj Aging Mech. Dis. 2017, 3, 8353. 10.1038/s41514-017-0009-8 PMC5460213 28649426

[305] J. L. Nielsen , D. Bakula , M. Scheibye‐Knudsen , Clin. Trials Target. Aging. Front Aging 2022. 10.3389/fragi.2022.820215 PMC9261384 35821843

[306] A. Rocha , S. Magalhães , A. Nunes , Curr. Metabolomics Syst. Biol. Discontin. 2021, 8, 1.

[307] L. Boisen , K. R. Drasbek , A. S. Pedersen , P. Kristensen , Exp. Gerontol. 2010, 45, 779. 20600781 10.1016/j.exger.2010.06.003

[308] M.‐K. Drekolia , S. Talyan , R. Cordellini Emídio , R. A. Boon , S. Guenther , M. Looso , G. Dumbovic , S.‐I. Bibli , Front. Genet. 2022, 13, 1035380. 36338971 10.3389/fgene.2022.1035380 PMC9634578

[309] J. van der Linden , L. Trap , C. V. Scherer , A. J. M. Roks , A. H. J. Danser , I. van der Pluijm , C. Cheng , Int. J. Mol. Sci. 2023, 24, 15379. 37895059 10.3390/ijms242015379 PMC10607365

[310] Y.u Yamazaki , D. J. Baker , M. Tachibana , C.‐C. Liu , J. M. van Deursen , T. G. Brott , G. Bu , T. Kanekiyo , Stroke 2016, 47, 1068. 26883501 10.1161/STROKEAHA.115.010835 PMC4811685

[311] E. E. Salmon , J. J. Breithaupt , G. A. Truskey , Cells 2020, 9, 1292. 32455928 10.3390/cells9051292 PMC7290800

[312] B. S. Fleenor , K. D. Marshall , C. Rippe , D. R. Seals , J. Vasc. Res. 2011, 49, 59. 21985896 10.1159/000329681 PMC3214888

[313] L. Atchison , H. Zhang , K. Cao , G. A. Truskey , Sci. Rep. 2017, 7, 8168. 28811655 10.1038/s41598-017-08632-4 PMC5557922

[314] D. Papadopoli , K. Boulay , L. Kazak , M. Pollak , F. A. Mallette , I. Topisirovic , L. Hulea , F1000Research 2019. 10.12688/f1000research.17196.1 PMC6611156 31316753

[315] J. M. Flynn , M. N. O'Leary , C. A. Zambataro , E. C. Academia , M. P. Presley , B. J. Garrett , A. Zykovich , S. D. Mooney , R. Strong , C. J. Rosen , P. Kapahi , M. D. Nelson , B. K. Kennedy , S. Melov , Aging Cell 2013, 12, 851. 23734717 10.1111/acel.12109 PMC4098908

[316] Y. Hattori , K. Suzuki , S. Hattori , K. Kasai , Hypertens Dallas Tex 2006, 47, 1183. 10.1161/01.HYP.0000221429.94591.72 16636195

[317] S. Chen , D. Gan , S. Lin , Y. Zhong , M. Chen , X. Zou , Z. Shao , G. Xiao , Theranostics 2022, 12, 2722. 35401820 10.7150/thno.71360 PMC8965502

[318] I. Y. Abdelgawad , K. Agostinucci , B. Sadaf , M. K. O. Grant , B. N. Zordoky , Front Aging 2023, 4, 1170434. 37168843 10.3389/fragi.2023.1170434 PMC10164964

[319] Y. Wang , M. Wang , Y. Wang , Aging Dis. 2023, 6, 2491. 10.14336/AD.2023.1112 PMC11567262 38029393

[320] Y. Zhang , Q. Mu , Z. Zhou , H. Song , Y. Zhang , F. Wu , M. Jiang , F. Wang , W. Zhang , L. Li , L. Shao , X. Wang , S. Li , L. Yang , Q. Wu , M. Zhang , D. Tang , PLoS One 2016, 11, 0158038. 10.1371/journal.pone.0158038 PMC4927070 27355581

[321] A. K. Palmer , M. Xu , Y. Zhu , T. Pirtskhalava , M. M. Weivoda , C. M. Hachfeld , L. G. Prata , T. H. van Dijk , E. Verkade , G. Casaclang‐Verzosa , K. O. Johnson , H. Cubro , E. J. Doornebal , M. Ogrodnik , D. Jurk , M. D. Jensen , E. N. Chini , J. D. Miller , A. Matveyenko , M. B. Stout , M. J. Schafer , T. A. White , L. J. Hickson , M. Demaria , V. Garovic , J. Grande , E. A. Arriaga , F. Kuipers , T. von Zglinicki , N. K. LeBrasseur , et al., Aging Cell 2019, 18, 12950. 10.1111/acel.12950 PMC6516193 30907060

[322] C. M. Roos , B. Zhang , A. K. Palmer , M. B. Ogrodnik , T. Pirtskhalava , N. M. Thalji , M. Hagler , D. Jurk , L. A. Smith , G. Casaclang‐Verzosa , Y.i Zhu , M. J. Schafer , T. Tchkonia , J. L. Kirkland , J. D. Miller , Aging Cell 2016, 15, 973. 26864908 10.1111/acel.12458 PMC5013022

[323] Z. S. Clayton , M. J. Rossman , S. A. Mahoney , R. Venkatasubramanian , G. S. Maurer , D. A. Hutton , N. S. VanDongen , N. T. Greenberg , A. G. Longtine , K. R. Ludwig , V. E. Brunt , T. J. LaRocca , J. Campisi , S. Melov , D. R. Seals , Hypertension 2023, 80, 2072. 37593877 10.1161/HYPERTENSIONAHA.123.21392 PMC10530538

[324] R. Gulej , Á. Nyúl‐Tóth , C. Ahire , J. DelFavero , P. Balasubramanian , T. Kiss , T. Kiss , S. Tarantini , Z. Benyo , P. Pacher , B. Csik , A. Yabluchanskiy , P. Mukli , A. Kuan‑Celarier , I. A. Krizbai , J. Campisi , W. E. Sonntag , A. Csiszar , Z. Ungvari , GeroScience. 2023, 45, 2983. 37642933 10.1007/s11357-023-00870-x PMC10643778

[325] A. E. Boe , M. Eren , S. B. Murphy , C. E. Kamide , A. Ichimura , D. Terry , D. McAnally , L. H. Smith , T. Miyata , D. E. Vaughan , Circulation 2013, 128, 2318. 24092817 10.1161/CIRCULATIONAHA.113.003192 PMC3933362

[326] V. Garcia , E. J. Park , M. Siragusa , F. Frohlich , M. Mahfuzul Haque , J. V. Pascale , K. R. Heberlein , B. E. Isakson , D. J. Stuehr , W. C. Sessa , Proc. Natl. Acad. Sci. 2020, 117, 9497. 32300005 10.1073/pnas.1918761117 PMC7196906

[327] F. C. Enevoldsen , J. Sahana , M. Wehland , D. Grimm , M. Infanger , M. Krüger , J. Clin. Med. 2020, 9, 824. 32197449 10.3390/jcm9030824 PMC7141375

[328] S. Nahar , S. Kanda , U. Chatha , V. A. Odoma , A. Pitliya , E. M. AlEdani , J. K. Bhangu , K. Javed , P. K. Manshahia , A. K. Yu , Cureus 2023, 15, 42748. 10.7759/cureus.42748 PMC10467640 37654961

[329] Y. Abdul , E. Karakaya , R. Chandran , S. Jamil , A. Ergul , Can. J. Physiol. Pharmacol. 2022, 100, 1087. 36384316 10.1139/cjpp-2022-0071 PMC10052805

[330] J. Deng , Q. Wu , Y. Liao , D. Huo , Z. Yang , Nephrology 2012, 17, 545. 22429568 10.1111/j.1440-1797.2012.01597.x

[331] R. Pisoni , J. Barker‐Finkel , M. Allo , Clin. J. Am. Soc. Nephrol. 2010, 5, 1447. 20507962 10.2215/CJN.02740310 PMC2924415

[332] C. Lymperopoulou , S. A. Kandarakis , I. Tzanaki , I. Mylona , T. Xanthos , A. P. Agouridis , Pharmaceuticals 2023, 16, 711. 37242493 10.3390/ph16050711 PMC10222124

[333] S. M. Lloyd , D. J. Stott , A. J. M. de Craen , P. M. Kearney , N. Sattar , I. Perry , C. J. Packard , A. Briggs , L. Marchbank , H. Comber , J. W. Jukema , R. G. J. Westendorp , S. Trompet , B. M. Buckley , I. Ford , PLoS One 2013, 8, 72642. 10.1371/journal.pone.0072642 PMC3759378 24023757

[334] J. Wang , J. Xu , C. Zhou , Y. Zhang , D.i Xu , Y. Guo , Z. Yang , J. Clin. Hypertens. 2012, 14, 245. 10.1111/j.1751-7176.2012.00600.x PMC8108932 22458746

[335] J. Etulain , H. A. Mena , R. P. Meiss , G. Frechtel , S. Gutt , S. Negrotto , M. Schattner , Sci. Rep. 2018, 8, 1513. 29367608 10.1038/s41598-018-19419-6 PMC5784112

[336] L. Padilla , R. Argüero‐Sánchez , J. M. Rodríguez‐Trejo , P. H. Carranza‐Castro , J. A. Suárez‐Cuenca , J. Polaco‐Castillo , M. DiSilvio‐López , J. López‐Gutiérrez , H. Olguín‐Juárez , A. Hernández‐Patricio , E. Vera‐Gómez , A. D.e J. Gómez‐Calderón , M. A. Téllez‐González , P. Mondragón‐Terán , J. Tissue Eng. Regen. Med. 2020, 14, 600. 32068332 10.1002/term.3024

[337] K. M. San Sebastian , I. Lobato , I. Hernández , N. Burgos‐Alonso , M. C. Gomez‐Fernandez , J. L. López , B. Rodríguez , A. Giné March , G. Grandes , I. Andia , B. Rodríguez , A. Giné March , G. Grandes , I. Andia , BMC Fam. Pract. 2014, 15, 211. 25547983 10.1186/s12875-014-0211-8 PMC4311495

[338] J.u Tian , J. Chen , X. Lai , J. Ding , J. Sun , D. Shi , X. He , X. Chen , Rev. Cardiovasc. Med. 2025, 26, 39383. 40776966 10.31083/RCM39383 PMC12326410

[339] R. A. Stein , C. Rockman , Y. Guo , M. A. Adelman , T. S. Riles , W. R. Hiatt , J. S. Berger , Arterioscler Thromb. Vasc. Biol. 2015, 35, 206. 25359858 10.1161/ATVBAHA.114.304161 PMC4518860

[340] W. Xia , J. Li , C. Su , Z. Yang , L. Chen , F. Wu , Y.‐Y. Zhang , B.‐B. Yu , Y.‐X. Qiu , S.‐M. Wang , J. Tao , Aging Cell 2012, 11, 111. 22018014 10.1111/j.1474-9726.2011.00758.x

[341] S. Endes , E. Schaffner , S. Caviezel , J. Dratva , C. S. Autenrieth , M. Wanner , B. Martin , D. Stolz , M. Pons , A. Turk , R. Bettschart , C. Schindler , N. Künzli , N. Probst‐Hensch , A. Schmidt‐Trucksäss , Age Ageing 2016, 45, 110. 26764400 10.1093/ageing/afv172

[342] I. Garcia‐Lunar , H. P. van der Ploeg , J. M. Fernández Alvira , F. van Nassau , J. M. Castellano Vázquez , A. J. van der Beek , X. Rossello , A. Fernández‐Ortiz , J. Coffeng , J. M. van Dongen , J. M. Mendiguren , W. van Mechelen , V. Fuster , Eur. Heart J. 20227, 43, 3732. 35869885 10.1093/eurheartj/ehac378 PMC9553098

[343] Y. Gando , K. Yamamoto , H. Murakami , Y. Ohmori , R. Kawakami , K. Sanada , M. Higuchi , I. Tabata , M. Miyachi , Hypertension 2010, 540. 20606102 10.1161/HYPERTENSIONAHA.110.156331

[344] U. R. Mikkelsen , C. Couppé , A. Karlsen , J. F. Grosset , P. Schjerling , A. L. Mackey , H. H. Klausen , S. P. Magnusson , M. Kjær , Mech. Ageing Dev. 2013, 531. 24287006 10.1016/j.mad.2013.11.004

[345] D. J. Green , C. E. Marsh , H. J. Thomas , L. Lester , K. J. Scurrah , A. Haynes , L. H. Naylor , Hypertension 2023, 80, 1343. 37073736 10.1161/HYPERTENSIONAHA.123.21090

[346] G. R. Lester , F. S. Abiusi , M. E. Bodner , P. M. Mittermaier , A. T. Cote , J. Vasc. Res. 2021, 59, 16. 34571505 10.1159/000518985

[347] T. Tarumi , M. M. Gonzales , B. Fallow , N. Nualnim , M. Pyron , H. Tanaka , A. P. Haley , J. Hypertens. 2013, 31, 2400.24220591 10.1097/HJH.0b013e328364decc

[348] A. Jennings , A. M. Berendsen , L. C. P. G. M. de Groot , E. J. M. Feskens , A. Brzozowska , E. Sicinska , B. Pietruszka , N. Meunier , E. Caumon , C. Malpuech‐Brugère , A. Santoro , R. Ostan , C. Franceschi , R. Gillings , C. M. O' Neill , S. J. Fairweather‐Tait , A.‐M. Minihane , A. Cassidy , Hypertens Dallas Tex. 2019, 73, 578. 10.1161/HYPERTENSIONAHA.118.12259 PMC6380440 30636547

[349] C. Marin , J. Delgado‐Lista , R. Ramirez , J. Carracedo , J. Caballero , P. Perez‐Martinez , F. M. Gutierrez‐Mariscal , A. Garcia‐Rios , N. Delgado‐Casado , C. Cruz‐Teno , E. M. Yubero‐Serrano , F. Tinahones , M. D. M. Malagon , F. Perez‐Jimenez , J. Lopez‐Miranda , Age 2012, 34, 1309. 21894446 10.1007/s11357-011-9305-6 PMC3528364

[350] E. M. Yubero‐Serrano , C. Fernandez‐Gandara , A. Garcia‐Rios , O. A. Rangel‐Zuñiga , F. M. Gutierrez‐Mariscal , J. D. Torres‐Peña , C. Marin , J. Lopez‐Moreno , J. P. Castaño , J. Delgado‐Lista , J. M. Ordovas , P. Perez‐Martinez , J. Lopez‐Miranda , PLoS Med. 2020, 17, 1003282. 10.1371/journal.pmed.1003282 PMC7480872 32903262

[351] M. Abdellatif , S. Sedej , D. Carmona‐Gutierrez , F. Madeo , G. Kroemer , Circ. Res. 2018, 123, 803. 30355077 10.1161/CIRCRESAHA.118.312208

[352] R. M. Broxterman , D. T. La Salle , J. Zhao , V. R. Reese , R. S. Richardson , J. D. Trinity , J. Appl. Physiol. Bethesda Md 2019, 127, 1085. 10.1152/japplphysiol.00371.2019 PMC6850980 31414959

[353] S. M. Bode‐Böger , J. Muke , A. Surdacki , G. Brabant , R. H. Böger , J. C. Frölich , Vasc. Med. Lond. Engl. 2003, 8, 77. 10.1191/1358863x03vm474oa 14518608

[354] S. Varadharaj , O. J. Kelly , R. N. Khayat , P. S. Kumar , N. Ahmed , J. L. Zweier , Front Cardiovasc Med. 2017, 4, 64. 29164133 10.3389/fcvm.2017.00064 PMC5671956

[355] Y. Cao , L. Cui , S. Tuo , H. Liu , S. Cui , Eur. J. Histochem. EJH 2024, 68, 4004. 38656259 10.4081/ejh.2024.4004 PMC11064893

[356] N. Omidifar , M. Moghadami , S. M. Mousavi , S. A. Hashemi , A. Gholami , M. Shokripour , Z. Sohrabi , Oxid Med. Cell Longev. 2021, 2021, 7501424. 34306314 10.1155/2021/7501424 PMC8270688

[357] M. Waldron , S. D. Patterson , J. Tallent , O. Jeffries , Curr. Hypertens. Rep. 2018, 20, 81. 30006901 10.1007/s11906-018-0881-z

[358] G. Santulli , U. Kansakar , F. Varzideh , P. Mone , S. S. Jankauskas , A. Lombardi , Nutrients 2023, 15, 4236. 37836520 10.3390/nu15194236 PMC10574552

[359] S. G. Ra , Y. Choi , N. Akazawa , K. Kawanaka , H. Ohmori , S. Maeda , Adv. Exp. Med. Biol. 2019, 1155, 407. 31468418 10.1007/978-981-13-8023-5_38

[360] D. M. Guizoni , J. F. Vettorazzi , E. M. Carneiro , A. P. Davel , Nitric Oxide 2020, 94, 48. 31669041 10.1016/j.niox.2019.10.008

[361] T. Kiss , C. B. Giles , S. Tarantini , A. Yabluchanskiy , P. Balasubramanian , T. Gautam , T. Csipo , Á. Nyúl‐Tóth , A. Lipecz , C. Szabo , E. Farkas , J. D. Wren , A. Csiszar , Z. Ungvari , GeroScience 2019, 41, 419. 31463647 10.1007/s11357-019-00095-x PMC6815288

[362] Y. Qiu , S. Xu , X. Chen , X. Wu , Z. Zhou , J. Zhang , S. Liu , C. Su , H. Huang , W. Xia , J. Tao , Signal Transduct Target Ther. 2023, 18, 353. 10.1038/s41392-023-01577-3 PMC10505611 37718359

[363] T. Kiss , Á. Nyúl‐Tóth , P. Balasubramanian , S. Tarantini , C. Ahire , A. Yabluchanskiy , T. Csipo , E. Farkas , J. D. Wren , L. Garman , A. Csiszar , Z. Ungvari , GeroScience 2020, 42, 527. 32056076 10.1007/s11357-020-00165-5 PMC7206476

[364] T. J. Sarkar , M. Quarta , S. Mukherjee , A. Colville , P. Paine , L. Doan , C. M. Tran , C. R. Chu , S. Horvath , L. S. Qi , N. Bhutani , T. A. Rando , V. Sebastiano , Nat. Commun. 2020, 11, 1545. 32210226 10.1038/s41467-020-15174-3 PMC7093390

[365] S. Bekaert , T. D. Meyer , P. V. Oostveldt , Anticancer Res. 2005, 25, 3011‐21. 16080560

[366] M. Cesari , B. W. J. H. Penninx , M. Pahor , F. Lauretani , A. M. Corsi , G. R. Williams , J. M. Guralnik , L. Ferrucci , J. Gerontol. Ser. A 2004, 59, M242. 10.1093/gerona/59.3.m242 15031308

[367] J.‐S. Paquette , C. Rhéaume , P. Cordeau , J.‐A. Moulin , E. Audet‐Walsh , V. Blanchette , J.‐P. Drouin‐Chartier , A.‐K. Toi , A. Tremblay , Metabolites 2023, 13, 1157. 37999253 10.3390/metabo13111157 PMC10673288

[368] A. E. Tchalla , G. A. Wellenius , T. G. Travison , M. Gagnon , I. Iloputaife , T. Dantoine , F. A. Sorond , L. A. Lipsitz , Hypertension 2015, 66, 340. 26056332 10.1161/HYPERTENSIONAHA.115.05180 PMC4807019

[369] L. Zhang , Y. Zhang , S. Xue , H. Ding , Y.u Wang , H. Qi , Y. Wang , W. Zhu , P. Li , J. Cell. Mol. Med. 2020, 24, 1146. 31709737 10.1111/jcmm.14802 PMC6933363

[370] V. Martínez‐Sales , I. Sánchez‐Lázaro , V. Vila , L. Almenar , T. Contreras , E. Reganon , Dis Markers 2011, 31, 757840. 10.3233/DMA-2011-0801 PMC3826486 21897001

[371] D. Poz , E. D. Falco , C. Pisano , R. Madonna , P. Ferdinándy , C. R. Balistreri , Rejuvenation Res. 2019, 22, 146. 30132390 10.1089/rej.2018.2094

[372] K. R. Gopcevic , E. Gkaliagkousi , J. Nemcsik , Ö. Acet , M. R. Bernal‐Lopez , R. M. Bruno , R. E. Climie , N. Fountoulakis , E. Fraenkel , A. Lazaridis , P. Navickas , K. D. Rochfort , A. Satrauskiene , J. Zupkauskiene , D. Terentes‐Printzios , Front Physiol. 2021, 12, 789690. 34970157 10.3389/fphys.2021.789690 PMC8712891

[373] A. Laucyte‐Cibulskiene , L. J. Ward , T. Ebert , G. Tosti , C. Tucci , L. Hernandez , A. Kautzky‐Willer , M.‐T. Herrero , C. M. Norris , L. Pilote , M. Söderberg , T. B. Brismar , J. Ripsweden , P. Stenvinkel , V. Raparelli , K. Kublickiene , Biol Sex Differ. 2021, 12, 50. 34526107 10.1186/s13293-021-00393-0 PMC8444580

[374] E. N. Dudinskaya , O. N. Tkacheva , M. V. Shestakova , N. V. Brailova , I. D. Strazhesko , D. U. Akasheva , O. Y. Isaykina , N. V. Sharashkina , D. A. Kashtanova , S. A. Boytsov , Endocr Connect 2015,4, 136. 26034119 10.1530/EC-15-0041 PMC4479064

[375] M. E. McNamara , N. Loyfer , A. J. Kiliti , M. O. Schmidt , S. Shabi‐Porat , S. S. Jain , S. Martinez Roth , A. P. McDeed , N. Shahrour , E. Ballew , Y.‐T. Lin , H.‐H. Li , A. Deslattes Mays , S. Rudra , A. T. Riegel , K. Unger , T. Kaplan , A. Wellstein , JCI Insight 2023, 8, 156529. 10.1172/jci.insight.156529 PMC10443812 37318863

[376] R. Dhingra , J. C. Nwanaji‐Enwerem , M. Samet , W.‐C. CK , Curr Environ Health Rep. 2018, 5, 317. 30047075 10.1007/s40572-018-0203-2 PMC6173330

[377] M. Moqri , C. Herzog , J. R. Poganik , K. Ying , J. N. Justice , D. W. Belsky , A. T. Higgins‐Chen , B. H. Chen , A. A. Cohen , G. Fuellen , S. Hägg , R. E. Marioni , M. Widschwendter , K. Fortney , P. O. Fedichev , A. Zhavoronkov , N. Barzilai , J. Lasky‐Su , D. P. Kiel , B. K. Kennedy , S. Cummings , P. E. Slagboom , E. Verdin , A. B. Maier , V. Sebastiano , M. P. Snyder , V. N. Gladyshev , S. Horvath , L. Ferrucci , Nat. Med. 2024, 30, 360. 38355974 10.1038/s41591-023-02784-9 PMC11090477

[378] A. M. Kulminski , I. Culminskaya , K. G. Arbeev , S. V. Ukraintseva , E. Stallard , L. Arbeeva , A. I. Yashin , Aging Cell 2013, 12, 237. 23320904 10.1111/acel.12046 PMC3602307

[379] S. R. Langley , K. Willeit , A. Didangelos , L. P. Matic , P. Skroblin , J. Barallobre‐Barreiro , M. Lengquist , G. Rungger , A. Kapustin , L. Kedenko , C. Molenaar , R. Lu , T. Barwari , G. Suna , X. Yin , B. Iglseder , B. Paulweber , P. Willeit , J. Shalhoub , G. Pasterkamp , A. H. Davies , C. Monaco , U. Hedin , C. M. Shanahan , J. Willeit , S. Kiechl , M. Mayr , J. Clin. Invest. 2017, 127, 1546. 28319050 10.1172/JCI86924 PMC5373893

[380] H. S.‐H. Oh , J. Rutledge , D. Nachun , R. Pálovics , O. Abiose , P. Moran‐Losada , D. Channappa , D. Y. Urey , K. Kim , Y. J. Sung , L. Wang , J. Timsina , D. Western , M. Liu , P. Kohlfeld , J. Budde , E. N. Wilson , Y. Guen , T. M. Maurer , M. Haney , A. C. Yang , Z. He , M. D. Greicius , K. I. Andreasson , S. Sathyan , E. F. Weiss , S. Milman , N. Barzilai , C. Cruchaga , A. D. Wagner , et al., Nature 2023, 624, 164. 38057571 10.1038/s41586-023-06802-1 PMC10700136

[381] G. Istas , K. Declerck , M. Pudenz , S. K. S. vel , V. Lendinez‐Tortajada , M. León‐Latre , K. Heyninck , G. Haegeman , J. A. Casasnovas , M. Tellez‑Plaza , C. Gerhauser , C. Heiss , A. Rodriguez‑Mateos , W. Vanden Berghe , Sci Rep. 2017, 7, 5120. 28698603 10.1038/s41598-017-03434-0 PMC5506022

[382] Z. Ahmed , S. Wan , F. Zhang , W. Zhong , BMC Methods 2024, 1, 4.

[383] F. Galkin , P. Mamoshina , K. Kochetov , D. Sidorenko , A. Zhavoronkov , Aging Dis. 2021, 12, 1252. 34341706 10.14336/AD.2020.1202 PMC8279523

[384] M. Arabfard , M. Ohadi , V. Rezaei Tabar , A. Delbari , K. Kavousi , BMC Genomics 2019, 20, 832. 31706268 10.1186/s12864-019-6140-0 PMC6842548

[385] G. Ghislat , T. Rahman , P. J. Ballester , Curr. Opin. Chem. Biol. 2021, 65, 28. 34052776 10.1016/j.cbpa.2021.04.009

[386] N. Sayed , Y. Huang , K. Nguyen , Z. Krejciova‐Rajaniemi , A. P. Grawe , T. Gao , R. Tibshirani , T. Hastie , A. Alpert , L. Cui , T. Kuznetsova , Y. Rosenberg‐Hasson , R. Ostan , D. Monti , B. Lehallier , S. S. Shen‐Orr , H. T. Maecker , C. L. Dekker , T. Wyss‐Coray , C. Franceschi , V. Jojic , F. Haddad , J. G. Montoya , J. C. Wu , M. M. Davis , D. Furman , Nat. Aging 2021, 1, 598. 34888528 10.1038/s43587-021-00082-y PMC8654267

[387] Y. Yu , L. Wang , W. Hou , Y. Xue , X. Liu , Y. Li , Front Immunol. 2024, 15, 1367235. 38686376 10.3389/fimmu.2024.1367235 PMC11056574

[388] J. W. Wills , J. R. Verma , B. J. Rees , D. S. G. Harte , Q. Haxhiraj , C. M. Barnes , R. Barnes , M. A. Rodrigues , M. Doan , A. Filby , R. E. Hewitt , C. A. Thornton , J. G. Cronin , J. D. Kenny , R. Buckley , A. M. Lynch , A. E. Carpenter , H. D. Summers , G. E. Johnson , P. Rees , Arch. Toxicol. 2021, 95, 3101. 34245348 10.1007/s00204-021-03113-0 PMC8380241

[389] T. Ba , S. Li , Y. Wei , Measurement 2021, 167, 108383.

[390] Aging Atlas Consortium . Nucleic Acids Res. 2021, 49, D825. 33119753 10.1093/nar/gkaa894 PMC7779027

[391] A. Zhavoronkov , E. Bischof , K. F. Lee , Nat Aging 2021, 1, 5. 37118000 10.1038/s43587-020-00020-4

[392] Z. Hu , J. Tang , Z. Wang , K. Zhang , L. Zhang , Q. Sun , Pattern Recognit. 2018, 83, 134.

[393] Y. J. Oh , J. Zhang , M. L. Fang , Y. Fukuoka , Int. J. Behav. Nutr. Phys. Act. 2021, 18, 160. 34895247 10.1186/s12966-021-01224-6 PMC8665320

[394] V. Smer‐Barreto , A. Quintanilla , R. J. R. Elliott , J. C. Dawson , J. Sun , V. M. Campa , Á. Lorente‐Macías , A. Unciti‐Broceta , N. O. Carragher , J. C. Acosta , D. A. Oyarzún , Nat. Commun. 2023, 14, 3445. 37301862 10.1038/s41467-023-39120-1 PMC10257182

[395] J. W. Wu , A. Yaqub , Y. Ma , W. Koudstaal , A. Hofman , M. A. Ikram , M. Ghanbari , J. Goudsmit , Sci. Rep. 2021, 11, 15929. 34354164 10.1038/s41598-021-95425-5 PMC8342513

[396] S. L. R. Bonnes , T. Strauss , A. K. Palmer , R. T. Hurt , L. Island , A. Goshen , L. Y. T. Wang , J. L. Kirkland , E. Bischof , A. B. Maier , GeroScience 2024, 46, 4217. 38512582 10.1007/s11357-024-01132-0 PMC11336016

[397] F. Galkin , O. Kovalchuk , D. Koldasbayeva , A. Zhavoronkov , E. Bischof , Ageing Res. Rev. 2023, 88, 101956. 37211319 10.1016/j.arr.2023.101956

[398] J. D. Faul , J. K. Kim , M. E. Levine , B. Thyagarajan , D. R. Weir , E. M. Crimmins , Proc. Natl. Acad. Sci. U S A. 2023, 120, 2215840120. 10.1073/pnas.2215840120 PMC9992763 36802439

[399] N. Hwangbo , X. Zhang , D. Raftery , H. Gu , S.‐C. Hu , T. J. Montine , J. F. Quinn , K. A. Chung , A. L. Hiller , D. Wang , Q. Fei , L. Bettcher , C. P. Zabetian , E. Peskind , G. Li , D. E. L. Promislow , A. Franks , J. Gerontol. A Biol. Sci. Med. Sci. 2021, 77, 744. 10.1093/gerona/glab212 PMC8974344 34382643

[400] M. N. Shokhirev , A. A. Johnson , Aging Cell 2021, 20, 13280. 10.1111/acel.13280 PMC7811842 33336875

[401] A. A. Johnson , M. N. Shokhirev , T. Wyss‐Coray , B. Lehallier , Ageing Res. Rev. 2020, 60, 101070. 32311500 10.1016/j.arr.2020.101070

[402] J. H. Cole , R. P. K. Poudel , D. Tsagkrasoulis , M. W. A. Caan , C. Steves , T. D. Spector , G. Montana , Neuroimage 2017, 115, 163. 10.1016/j.neuroimage.2017.07.059 28765056

[403] S. Fong , K. Pabis , D. Latumalea , N. Dugersuren , M. Unfried , N. Tolwinski , B. Kennedy , J. Gruber , Nat Aging 2024, 4, 1137. 38898237 10.1038/s43587-024-00646-8 PMC11333290

[404] A. T. Lu , A. Quach , J. G. Wilson , A. P. Reiner , A. Aviv , K. Raj , L. Hou , A. A. Baccarelli , Y. Li , J. D. Stewart , E. A. Whitsel , T. L. Assimes , L. Ferrucci , S. Horvath , Aging 2019, 11, 303. 30669119 10.18632/aging.101684 PMC6366976

[405] M. E. Levine , A. T. Lu , A. Quach , B. H. Chen , T. L. Assimes , S. Bandinelli , L. Hou , A. A. Baccarelli , J. D. Stewart , Y. Li , E. A. Whitsel , J. G. Wilson , A. P. Reiner , A. Aviv , K. Lohman , Y. Liu , L. Ferrucci , S. Horvath , Aging 2018, 10, 573. 29676998 10.18632/aging.101414 PMC5940111

[406] D. W. Belsky , A. Caspi , D. L. Corcoran , K. Sugden , R. Poulton , L. Arseneault , A. Baccarelli , K. Chamarti , X. Gao , E. Hannon , H. L. Harrington , R. Houts , M. Kothari , D. Kwon , J. Mill , J. Schwartz , P. Vokonas , C. Wang , B. S. Williams , T. E. Moffitt , eLife. 2022, 11, 73420. 10.7554/eLife.73420 PMC8853656 35029144

[407] J. Rutledge , H. Oh , T. Wyss‐Coray , Nat. Rev. Genet. 2022, 23, 715. 35715611 10.1038/s41576-022-00511-7 PMC10048602

[408] Z. Huang , J. Zhang , H. Shan , IEEE Trans Pattern Anal. Mach. Intell. 2023, 45, 7917. 36306297 10.1109/TPAMI.2022.3217882

[409] Y. Pan , V. Nallanthighal , D. Blackburn , H. Christensen , A. Härmä , Academic 2021, 7258.

[410] I. Subramanian , S. Verma , S. Kumar , A. Jere , K. Anamika , Bioinforma Biol Insights 2020, 14, 1177932219899051. 10.1177/1177932219899051 PMC7003173 32076369

[411] L. Wu , X. Xie , T. Liang , J. Ma , L. Yang , J. Yang , L. Li , Y. Xi , H. Li , J. Zhang , X. Chen , Y. Ding , Q. Wu , Biomolecules 2021, 12, 39. 35053186 10.3390/biom12010039 PMC8773837

